# Cardiomyocyte-specific PCSK9 deficiency compromises mitochondrial bioenergetics and heart function

**DOI:** 10.1093/cvr/cvad041

**Published:** 2023-03-06

**Authors:** Marion Laudette, Malin Lindbom, Muhammad Arif, Mathieu Cinato, Mario Ruiz, Stephen Doran, Azra Miljanovic, Mikael Rutberg, Linda Andersson, Martina Klevstig, Marcus Henricsson, Per-Olof Bergh, Entela Bollano, Nay Aung, J Gustav Smith, Marc Pilon, Tuulia Hyötyläinen, Matej Orešič, Rosie Perkins, Adil Mardinoglu, Malin C Levin, Jan Borén

**Affiliations:** Department of Molecular and Clinical Medicine/Wallenberg Laboratory, Institute of Medicine, The Sahlgrenska Academy, University of Gothenburg, Gothenburg 40530, Sweden; Department of Molecular and Clinical Medicine/Wallenberg Laboratory, Institute of Medicine, The Sahlgrenska Academy, University of Gothenburg, Gothenburg 40530, Sweden; Science for Life Laboratory, Royal Institute of Technology, Stockholm 17165, Sweden; Department of Molecular and Clinical Medicine/Wallenberg Laboratory, Institute of Medicine, The Sahlgrenska Academy, University of Gothenburg, Gothenburg 40530, Sweden; Department of Chemistry and Molecular Biology, University of Gothenburg, Gothenburg 40530, Sweden; Faculty of Dentistry, Oral & Craniofacial Sciences, Centre for Host-Microbiome Interactions, King’s College London, London WC2R 2L2, UK; Department of Molecular and Clinical Medicine/Wallenberg Laboratory, Institute of Medicine, The Sahlgrenska Academy, University of Gothenburg, Gothenburg 40530, Sweden; Department of Molecular and Clinical Medicine/Wallenberg Laboratory, Institute of Medicine, The Sahlgrenska Academy, University of Gothenburg, Gothenburg 40530, Sweden; Department of Molecular and Clinical Medicine/Wallenberg Laboratory, Institute of Medicine, The Sahlgrenska Academy, University of Gothenburg, Gothenburg 40530, Sweden; Department of Molecular and Clinical Medicine/Wallenberg Laboratory, Institute of Medicine, The Sahlgrenska Academy, University of Gothenburg, Gothenburg 40530, Sweden; Department of Molecular and Clinical Medicine/Wallenberg Laboratory, Institute of Medicine, The Sahlgrenska Academy, University of Gothenburg, Gothenburg 40530, Sweden; Department of Molecular and Clinical Medicine/Wallenberg Laboratory, Institute of Medicine, The Sahlgrenska Academy, University of Gothenburg, Gothenburg 40530, Sweden; Department of Cardiology, Sahlgrenska University Hospital, Gothenburg 41345, Sweden; William Harvey Research Institute, Barts and The London School of Medicine and Dentistry, Queen Mary University of London, London E1 4NS, UK; National Institute for Health Research, Barts Cardiovascular Biomedical Research Centre, Queen Mary University of London, London SW15 5PN, UK; Barts Heart Centre, St Bartholomew’s Hospital, Barts Health National Health Service Trust, West Smithfield EC1A 7BE, UK; Department of Molecular and Clinical Medicine/Wallenberg Laboratory, Institute of Medicine, The Sahlgrenska Academy, University of Gothenburg, Gothenburg 40530, Sweden; Department of Cardiology, Sahlgrenska University Hospital, Gothenburg 41345, Sweden; Department of Chemistry and Molecular Biology, University of Gothenburg, Gothenburg 40530, Sweden; School of Natural Sciences and Technology, Örebro University, Örebro 70182, Sweden; School of Medical Sciences, Örebro University, Örebro 70182, Sweden; Turku Bioscience Centre, University of Turku, Turku 20520, Finland; Department of Molecular and Clinical Medicine/Wallenberg Laboratory, Institute of Medicine, The Sahlgrenska Academy, University of Gothenburg, Gothenburg 40530, Sweden; Science for Life Laboratory, Royal Institute of Technology, Stockholm 17165, Sweden; Faculty of Dentistry, Oral & Craniofacial Sciences, Centre for Host-Microbiome Interactions, King’s College London, London WC2R 2L2, UK; Department of Molecular and Clinical Medicine/Wallenberg Laboratory, Institute of Medicine, The Sahlgrenska Academy, University of Gothenburg, Gothenburg 40530, Sweden; Department of Molecular and Clinical Medicine/Wallenberg Laboratory, Institute of Medicine, The Sahlgrenska Academy, University of Gothenburg, Gothenburg 40530, Sweden; Sahlgrenska University Hospital, Gothenburg 41345, Sweden

**Keywords:** Pro-protein convertase subtilisin-kexin type 9 (PCSK9), Cardiomyocyte, Mitochondria, Cardiac dysfunction, Metabolic inflexibility

## Abstract

**Aims:**

Pro-protein convertase subtilisin-kexin type 9 (PCSK9), which is expressed mainly in the liver and at low levels in the heart, regulates cholesterol levels by directing low-density lipoprotein receptors to degradation. Studies to determine the role of PCSK9 in the heart are complicated by the close link between cardiac function and systemic lipid metabolism. Here, we sought to elucidate the function of PCSK9 specifically in the heart by generating and analysing mice with cardiomyocyte-specific *Pcsk9* deficiency (CM-*Pcsk9*^−/−^ mice) and by silencing *Pcsk9* acutely in a cell culture model of adult cardiomyocyte-like cells.

**Methods and results:**

Mice with cardiomyocyte-specific deletion of *Pcsk9* had reduced contractile capacity, impaired cardiac function, and left ventricular dilatation at 28 weeks of age and died prematurely. Transcriptomic analyses revealed alterations of signalling pathways linked to cardiomyopathy and energy metabolism in hearts from CM*-Pcsk9*^−/−^ mice vs. wild-type littermates. In agreement, levels of genes and proteins involved in mitochondrial metabolism were reduced in CM*-Pcsk9*^−/−^ hearts. By using a Seahorse flux analyser, we showed that mitochondrial but not glycolytic function was impaired in cardiomyocytes from CM*-Pcsk9*^−/−^ mice. We further showed that assembly and activity of electron transport chain (ETC) complexes were altered in isolated mitochondria from CM*-Pcsk9*^−/−^ mice. Circulating lipid levels were unchanged in CM*-Pcsk9*^−/−^ mice, but the lipid composition of mitochondrial membranes was altered. In addition, cardiomyocytes from CM*-Pcsk9*^−/−^ mice had an increased number of mitochondria–endoplasmic reticulum contacts and alterations in the morphology of cristae, the physical location of the ETC complexes. We also showed that acute *Pcsk9* silencing in adult cardiomyocyte-like cells reduced the activity of ETC complexes and impaired mitochondrial metabolism.

**Conclusion:**

PCSK9, despite its low expression in cardiomyocytes, contributes to cardiac metabolic function, and PCSK9 deficiency in cardiomyocytes is linked to cardiomyopathy, impaired heart function, and compromised energy production.

## Graphical Abstract

**Graphical Abstract:**
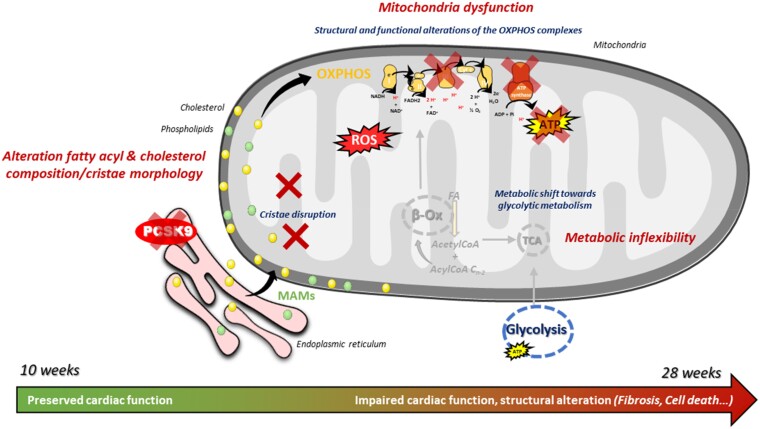
Model of the effects of Pcsk9 deletion in the heart. We propose that cardiac PCSK9 maintains the normal composition of cholesterol and phospholipids in the mitochondrial membrane by modulating their transport through mitochondria-associated ER membrane contacts (MAMs). Reduced cardiac Pcsk9 expression alters the lipid composition of mitochondrial membranes and cristae morphology, leading to impaired assembly and activity of mitochondrial respiratory complexes (especially ATP synthase), and thereby decreased OXPHOS and ATP production, and increased ROS production. These impairments indicate metabolic inflexibility characterized by reduced mitochondrial oxidative metabolism and insufficient compensatory energy production by glycolytic pathways, making the heart less efficient in generating energy to support demand. PCSK9 cardiac deficiency leads to progressive DCM, impaired heart function/structure, and compromised energy production. TCA, tricarboxylic acid cycle

**Time of primary review: 46 days**

**Translational perspective**

Pro-protein convertase subtilisin-kexin type 9 (PCSK9) is mainly present in the circulation where it regulates plasma cholesterol levels. Here, we show that PCSK9 mediates intracellular functions that differ from its extracellular functions. We further show that intracellular PCSK9 in cardiomyocytes, despite low expression levels, is important for maintaining physiological cardiac metabolism and function.

## 1. Introduction

Cholesterol homeostasis is vital for cellular and systemic functions. Less than 20 years ago, the first case of a novel gain-of-function mutation linked to autosomal dominant hypercholesterolaemia was described in *PCSK9*, the gene encoding pro-protein convertase subtilisin-kexin type 9 (PCSK9).^1,2^ Studies revealed that PCSK9 targets the low-density lipoprotein (LDL) receptor (LDLR) for lysosomal degradation in the liver, and PCSK9 is now recognized as a critical regulator of cholesterol homeostasis.^3^ PCSK9 has been exploited as a drug target for hypercholesterolaemia, and anti-PCSK9 monoclonal antibodies have been shown to markedly reduce plasma LDL-cholesterol levels.^4^ However, PCSK9 monoclonal antibodies are expensive to produce, and require once- or twice-monthly subcutaneous injections. Therefore, non-antibody treatments to inhibit PCSK9 function are being developed. These include gene-silencing or editing technologies, such as small interfering RNA.^5^ Importantly, monoclonal antibodies target extracellular PCSK9 only, whereas gene-silencing technologies reduce both extracellular and intracellular PCSK9 levels.

PCSK9 is expressed in many tissues in addition to the liver, albeit more weakly,^6^ supporting extra-hepatic functions beyond LDLR degradation.^7–9^ A recent study showed that whole-body *Pcsk9* deficiency in mice impairs cardiac lipid metabolism in an LDLR-independent manner and promotes heart failure.^10^ However, this finding contrasts with earlier reports showing that lowering PCSK9 levels has a beneficial effect on cardiac responses after a myocardial infarction.^11–13^ Identifying the specific function of PCSK9 in the heart is complicated by the fact that cardiac function is closely linked to systemic lipid metabolism.^14,15^ Given that whole-body deficiency of *Pcsk9* in mice has a profound effect on lipoprotein metabolism and cardiac uptake of lipids, heart-selective knockout models are required to determine the specific role of PCSK9 in the heart.

In this study, we sought to elucidate the function of PCSK9 specifically in the heart by generating and analysing mice with cardiomyocyte-specific *Pcsk9* deficiency (CM*-Pcsk9*^−/−^ mice) and cell culture models. We demonstrate that cardiomyocyte expression of *Pcsk9* contributes to normal mitochondrial function and that CM*-Pcsk9*^−/−^ is linked to heart failure.

## 2. Methods

In addition to the methods described below, a detailed description of echocardiographic analyses, isolation of primary cardiomyocytes, measurement of lactate dehydrogenase (LDH) release, culture and differentiation of H9c2 cells, lipidomics, immunohistochemistry and immunofluorescence, transcriptomics, gene expression analysis, immunoblotting, isolation and analysis of mitochondria from mouse hearts, transmission electron microscopy (TEM) of mouse hearts and mitochondria, metabolic profiling of primary cardiomyocytes, mitochondria and H9c2 cardiomyocytes (using the Seahorse analyser), analysis of mitochondrial complexes and supercomplexes (SCs) and in-gel activity, and measurement of mitochondrial membrane potential and reactive oxygen species is described in the Supplementary Material.

### 2.1 Mice

All procedures in mice were approved by the local Animal Ethics Committee in Gothenburg (breeding ethics approval 1124-2017) and conform to the guidelines in Directive 2010/63/EU of the European Parliament on the protection of animals used for scientific purposes. Mice were killed by cervical dislocation after an overdose of isoflurane (Forene, AbbVie) (dose 5%).

Mice were kept under temperature-controlled conditions with free access to water and standard rodent chow (12% of calories from protein, 12% from fat, and 66% from carbohydrates) and housed in a pathogen-free barrier facility with a 12 h light, 12 h dark cycle. Mice were anaesthetized with continuous artificially ventilated isoflurane (Forene, AbbVie) (dose 1.2%) through nose inhalation for the complete period of all interventions.

*Pcsk9^LoxP/LoxP^* mice, in which exons 2 and 3 were flanked with loxP sites, were generated and characterized as described^16^ and crossbred with α-myosin heavy chain (α-MHC)-Cre mice [B6.FVB-Tg(Myh6-cre)2182Mds/J, Jackson Laboratory] to generate cardiomyocyte-specific *Pcsk9* knockdown mice (CM*-Pcsk9*^−/−^; *Pcsk9^LoxP/LoxP^*/α-MHC-Cre) and non-transgenic littermate controls (CM*-Pcsk9*^+/+^; *Pcsk9^LoxP/LoxP^*). Expression analysis revealed that the *Pcsk9* gene deletion did not affect expression of genes located close to the *psck9* locus in CM*-Pcsk9*^−/−^ mice (see Supplementary material online, *Table S1*). Plasma, hearts, and other tissues were taken after a 4 h fast.

### 2.2 Transfection of H9c2 cardiomyocytes

H9c2 cells (CRL-1446, America Tissue Type Collection) were differentiated into H9c2 cardiomyocytes as described in the Supplementary Material. For *Pcsk9* silencing experiments, H9c2 cardiomyocytes were transfected with rat PCSK9-−specific siRNA and control siRNA (20 µmol/L, Horizon Discovery) in distilled water and Lipofectamine RNAiMax transfection reagent (Invitrogen). For *Pcsk9* overexpression experiments, H9c2 cardiomyocytes were transfected with PCSK9-GFP cDNA (1 µg) or empty control CT-GFP (Sinobiological) in Optimem Reduced Serum and Lipofectamine LTX and Plus Reagent (Invitrogen).

### 2.3 Quantification and statistical analysis

Values are reported as mean ± SEM unless otherwise indicated. Details of the statistical analysis, including numbers of mice, are indicated in the figure legends. Unpaired two-tailed *t*-tests were used to compare two groups; two-way ANOVA followed by Sidak’s multiple comparisons test was used to compare more than two groups. Survival was assessed with the log-rank test. *P* < 0.05 was considered statistically significant. GraphPad Prism was used for all statistical analyses and graphics.

## 3. Results

### 3.1 CM*-Pcsk9^−/−^* mice have heart failure and die prematurely

**Figure 1:**
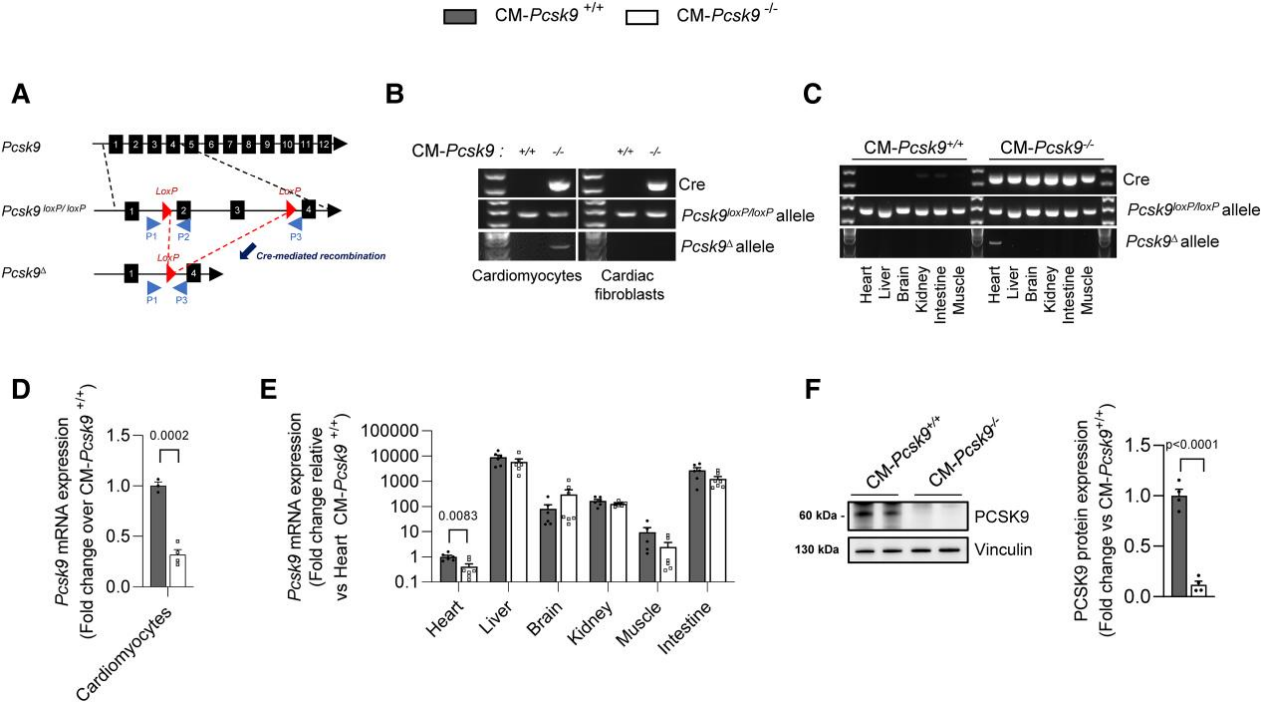
Characterization of CM-*Pcsk9*^−/−^ mice. (*A*) Schematic of conditional knockout of Pcsk9. Exons 2 and 3 were flanked with loxP sites. Cre-mediated recombination led to a Pcsk9Δ allele. Half arrows indicate the P1–P3 primers used for genotyping and model validation. (*B* and *C*) Analysis of genomic DNA from (*B*) isolated primary cardiomyocytes and cardiac fibroblasts and (*C*) the indicated tissues from CM-*Pcsk9*^+/+^ and CM-*Pcsk9*^−/−^ mice with PCR primers targeting the *Pcsk9*loxP allele, the *Pcsk9*Δ allele, and αMHC-Cre. (*D* and *E*) Quantification of *Pcsk9* mRNA expression in (*D*) primary cardiomyocytes (*n* = 4) and (*E*) the indicated tissues (*n* = 6–7) from CM-*Pcsk9*^+/+^ and CM-*Pcsk9*^−/−^ mice, determined with a probe covering exons 3 and 4. (*F*) Representative immunoblots and quantification of PCSK9 protein levels in isolated primary cardiomyocytes from CM-*Pcsk9*^+/+^ and CM-*Pcsk9*^−/−^ mice hearts (*n* = 4). Vinculin was the loading control. Values are mean ± SEM. *P* values are shown in the figure vs. CM-*Pcsk9*^+/+^ by two-tailed *t*-test.

**Figure 2:**
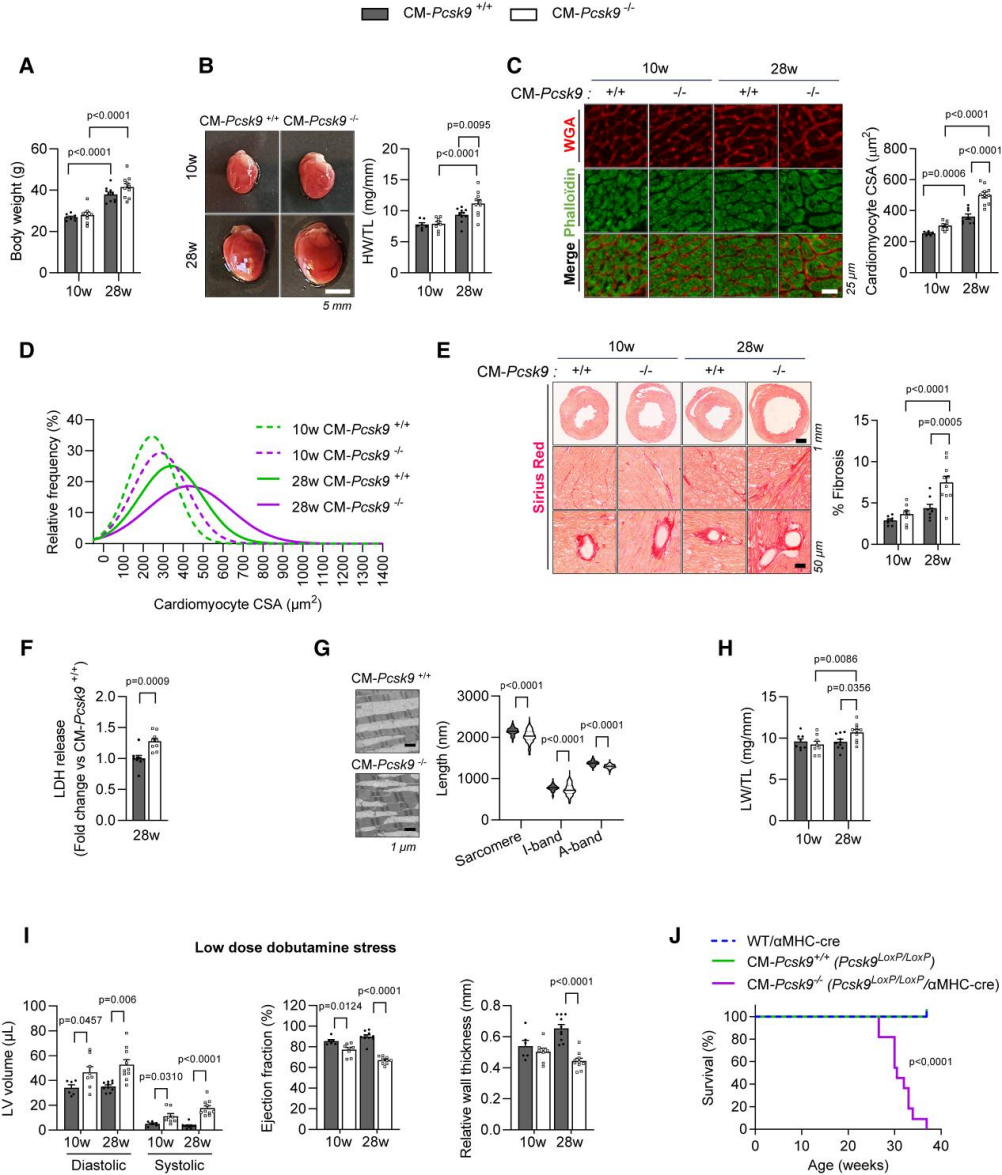
CM*-Pcsk9*^−/−^ mice have heart failure and die prematurely. (*A*) Body weight and (*B*) photographs of hearts (left) and heart weight/tibia length (HW/TL) (right) of 10- and 28-week-old CM*-Pcsk9*^+/+^ and CM*-Pcsk9*^−/−^ mice (*n* = 7–10). Scale, 5 mm. (*C*) Representative images of cardiomyocytes stained with fluorescent wheat germ agglutinin (WGA) (red) and phalloidin (green) (left) and quantification (right) of cross-sectional area (CSA) of cardiomyocytes from 10- and 28-week-old CM*-Pcsk9*^+/+^ and CM*-Pcsk9*^−/−^ mice (*n* = 7–10). Scale, 25 µm. (*D*) Relative frequency of cross-sectional area of cardiomyocytes from 10- and 28-week-old CM*-Pcsk9*^+/+^ and CM*-Pcsk9*^−/−^ mice (≥1600 cells; *n* = 7–10). (*E*) Representative Sirius Red-stained heart sections from 10- and 28-week-old CM*-Pcsk9*^+/+^ and CM*-Pcsk9*^−/−^ mice (left) and quantification of fibrosis (right) (*n* = 8–10). Scale, 1 mm or 25 µm. (*F*) Lactate dehydrogenase (LDH) release from cardiomyocytes isolated from 28-week-old CM*-Pcsk9*^+/+^ and CM*-Pcsk9*^−/−^ mice (*n* = 9). (*G*) Representative transmission electron microscope images (left) and quantification (right) of sarcomere, I-band and A-band length from LV tissue of 28-week-old CM*-Pcsk9*^+/+^ and CM*-Pcsk9*^−/−^ mice (≥472 sarcomeres; *n* = 3). Scale, 1 µm. (*H*) Lung weight/tibia length (LW/TL) of 10- and 28-week-old CM*-Pcsk9*^+/+^ and CM*-Pcsk9*^−/−^ mice (*n* = 7–10). (*I*) Cardiac function during dobutamine stress in 10- and 28-week-old CM*-Pcsk9*^+/+^ and CM*-Pcsk9*^−/−^ mice (*n* = 10; see also Supplementary material online, *Table S2*). (*J*) Kaplan–Meier survival curve of wild-type mice expressing αMHC-Cre (WT/αMHC-Cre), CM-*Pcsk9*^+/+^ and CM-*Pcsk9*^−/−^ mice (*n* = 7–11). Values are mean ± SEM. *P* values are shown vs. CM*-Pcsk9*^+/+^ by two-tailed *t*-test (*F*, *G*, *I*, *J*) or two-way ANOVA (*A*, *B*, *C*, *E*, *H*).

To determine whether PCSK9 has a selective role in the heart, we generated mice in which exons 2 and 3 of *Pcsk9* were flanked by loxP sites and bred them with αMHC-Cre mice to allow cardiomyocyte-specific deletion of *Pcsk9* (*Figure 1A*). PCR analyses showed reduced expression of *Pcsk9* in heart (by 53%) and cardiomyocytes (by 62%), but not in liver, kidney, skeletal muscle, brain, small intestine, or cardiac fibroblasts of CM*-Pcsk9^−/−^* mice (*Figure 1B–E*). Cardiomyocyte-specific deletion of *Pcsk9* was confirmed at protein levels in isolated cardiomyocytes (*Figure 1F*). Body weight (*Figure 2A*), circulating levels of lipids and PCSK9, and lipid and ceramide levels in heart and liver (see Supplementary material online, *Figure S1A* and *B*) were mostly similar in CM*-Pcsk9^−/−^* and CM*-Pcsk9^+/+^* mice, suggesting little effect of *Pcsk9* deficiency on systemic lipid homeostasis.

Heart weight (per tibia length) and cardiomyocyte cross-sectional area of CM*-Pcsk9^−/−^* mice were similar to those of CM*-Pcsk9^+/+^* mice at 10 weeks, but greater in CM*-Pcsk9^−/−^* than CM*-Pcsk9^+/+^* mice at 28 weeks (*Figure 2B–D*), indicating cardiac hypertrophy. CM*-Pcsk9^−/−^* mice at 28 weeks also had higher levels of cardiac interstitial fibrosis (*Figure 2E*), left ventricular (LV) dilatation (see Supplementary material online, *Table S2*), cardiomyocyte death (*Figure 2F*), and distorted, misaligned sarcomeres and myocardial filaments (*Figure 2G*), consistent with dilated cardiomyopathy. The lung weight (per tibia length) (*Figure 2H*) and LV diameter and volume (see Supplementary material online, *Table S2*) were also greater in CM*-Pcsk9^−/−^* mice at 28 weeks, indicating heart failure and pulmonary congestion. In response to dobutamine-induced myocardial stress, CM*-Pcsk9^−^*^/−^ mice had reduced contractile capacity and cardiac reserve at both 10 and 28 weeks and a pronounced reduction in relative wall thickness at 28 weeks (*Figure 2I* and Supplementary material online, *Table S2*). Furthermore, CM*-Pcsk9^−/−^* mice died suddenly beginning at 28 weeks of age and had markedly shortened lifespans (<37 weeks, *Figure 2J*). Of note, expression of the αMHC-Cre transgene alone did not promote cardiac hypertrophy in 28-week-old mice (see Supplementary material online, *Figure S1C*) or contribute to the onset of heart failure (*Figure 2J*).

These results are agreement with the recent report in whole-body *Pcsk9^−^*^/−^ mice showing that *Pcsk9* deficiency drives heart failure,^10^ and importantly confirm that *Pcsk9* has a selective role in the heart.

### 3.2 Cardiac mitochondrial metabolism is impaired in CM*-Pcsk9^−/−^* mice

**Figure 3:**
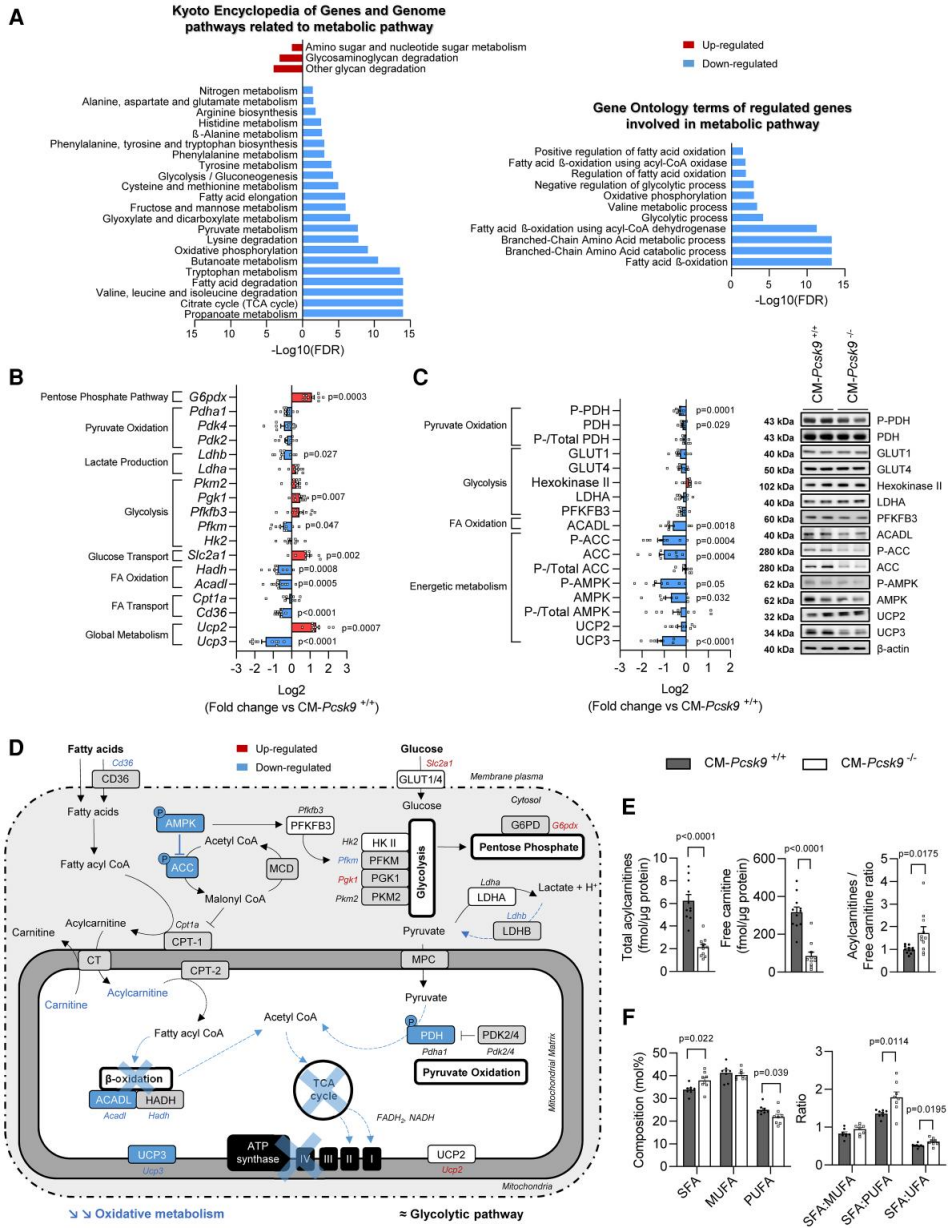
Cardiac mitochondrial metabolism is impaired in CM*-Pcsk9*^−/−^ mice. (*A*) Significantly up- and down-regulated Gene Ontology biological processes and Kyoto Encyclopedia of Genes and Genome pathways related to metabolic pathways in RNA-seq data from hearts from 28-week-old CM*-Pcsk9*^−/−^ vs. CM*-Pcsk9*^+/+^ mice (*n* = 5). RNA-seq statistics were retrieved from gene set statistics from PIANO analysis. FDR, false-discovery rate. (*B*) Variation plots of genes (mRNA) related to cardiac metabolism in hearts of 28-week-old CM*-Pcsk9*^−/−^ vs. CM*-Pcsk9*^+/+^ mice (*n* = 8–10). (*C*) Left: Variation plots of proteins related to cardiac metabolism in cardiomyocytes from 28-week-old CM*-Pcsk9*^−/−^ vs. CM*-Pcsk9*^+/+^ mice (*n* = 12). Right: representative immunoblots. β-actin was the loading control. (*D*) Schematic metabolic network showing alterations in genes and proteins in 28-week-old CM*-Pcsk9*^−/−^ vs. CM*-Pcsk9*^+/+^ mouse hearts. Proteins (in boxes) and mRNA (in italics) that were significantly modulated are coloured. Unexplored proteins are indicated in grey. (*E*) Total acyl- and free carnitine content and their ratio in cardiac mitochondria from 28-week-old CM*-Pcsk9*^+/+^ and CM*-Pcsk9*^−/−^ mice (*n* = 11). (*F*) Saturated fatty acids (SFA), monounsaturated fatty acids (MUFA), polyunsaturated fatty acids (PUFA), and their ratio in isolated cardiomyocytes from 28-week-old CM*-Pcsk9*^+/+^ and CM*-Pcsk9*^−/−^ mice (*n* = 8). UFA, total unsaturated fatty acids. Data are mean ± SEM. *P* values are shown vs. CM*-Pcsk9^+/+^* by two-tailed *t*-test.

To further understand the onset of heart failure in CM*-Pcsk9^−^*^/−^ mice, we performed RNA sequencing (RNA-seq) on hearts from 10- and 28-week-old mice. Expression of 58 and 1798 genes differed between CM*-Pcsk9*^−/−^ and CM*-Pcsk9*^+/+^ mice at 10 and 28 weeks, respectively (false-discovery rate < 0.05). Gene Ontology and Kyoto Encyclopedia of Genes and Genomes pathway analyses showed downregulation of genes involved in cardiac muscle contraction and dilated cardiomyopathy in CM*-Pcsk9^−^*^/−^ mice (see Supplementary material online, *Figure S2A* and *B*, *Table S3*); these results were confirmed by targeted RNA analysis (see Supplementary material online, *Figure S2C* and *D*). Pathway analyses also revealed strong downregulation of pathways involved in mitochondrial metabolism in CM*-Pcsk9^−^*^/−^ mice, including the tricarboxylic acid (TCA) cycle, fatty acid β-oxidation, degradation of branched-chain amino acids, oxidative phosphorylation (OXPHOS), and pyruvate metabolism (*Figure 3A*).

Impairment of mitochondrial metabolism in hearts from CM*-Pcsk9^−^*^/−^ mice at 28 weeks was further shown by reductions in: (i) expression of *Cd36*, which encodes a membrane protein involved in fatty acid uptake; (ii) expression of *Acadl* and *Hadh*, encoding acyl-CoA dehydrogenase long chain (ACADL) and hydroxyacyl-CoA dehydrogenase (HADH), key enzymes in mitochondrial β-oxidation, (iii) protein levels of ACADL; (iv) total and phosphorylated protein levels of adenosine monophosphate-activated protein kinase (AMPK; a master regulator of energetic metabolism and mitochondrial homeostasis^17^) and its effector acetyl-CoA carboxylase (regulator of carnitine palmitoyltransferase 1, the rate-limiting enzyme in β-oxidation); (v) gene and protein levels of uncoupling protein 3, which is known to correlate with markers of mitochondrial β-oxidation;^18^ (vi) total and phosphorylated protein levels of pyruvate dehydrogenase, a key mitochondrial enzyme that converts pyruvate to acetyl-CoA and thereby fuels the TCA cycle; and (vii) expression of *Ldhb*, encoding lactate dehydrogenase B (LDHB), a key glycolytic enzyme that converts lactate to pyruvate (*Figure 3B–D*). In contrast, *Pcsk9* deficiency did not reduce the expression of genes and levels of proteins involved in glucose uptake and glycolysis in mouse hearts (with the exception of a slight reduction in *Pfkm*) (*Figure 3B–D*). CM*-Pcsk9^−^*^/−^ mice had higher expression of *G6pdx*, which encodes glucose-6-phosphate 1-dehydrogenase X, the rate-limiting enzyme in the pentose phosphate pathway (a glucose metabolic pathway parallel to glycolysis) (*Figures 2D* and *3B*). In addition, CM*-Pcsk9^−^*^/−^ mice had higher expression of *Ucp2*, which is known to reflect a glycolytic phenotype^18^; however, there was no change in UCP2 protein levels (*Figure 3B–D*). In summary, these results show that *Pcsk9* deficiency in mouse hearts results in downregulation of mitochondrial fatty acid metabolism and an increased dependence on glycolytic pathways in an attempt to meet the energy demands of cells.

We also showed that mitochondria from CM*-Pcsk9^−^*^/−^ mouse hearts had marked decreases in free carnitine and acylcarnitine species (*Figure 3E* and see Supplementary material online, *Figure S2E*), which are involved in mitochondrial fatty acid transport and metabolism,^19^ and a high acylcarnitine to free carnitine ratio (*Figure 3E*), indicating a low capacity for energy production. Furthermore, despite similar lipid levels in cardiomyocytes (see Supplementary material online, *Figure S2F*), the ratio of saturated to unsaturated fatty acids was higher in cardiomyocytes from 28-week-old CM*-Pcsk9*^−/−^ vs. CM*-Pcsk9*^+/+^ mice (*Figure 3F*), consistent with altered membrane and cellular functions such as mitochondrial β-oxidation.^20^ However, we did not observe any alterations in cardiomyocyte membrane fluidity either under basal conditions or when exposed to palmitate challenge (see Supplementary material online, *Figure S2G*).

### 3.3 Impairment of mitochondrial but not glycolytic function in CM*-Pcsk9*^−/−^ cardiomyocytes

To assess the bioenergetic profile of CM*-Pcsk9*^−/−^ cardiomyocytes, we used a Seahorse flux analyser to measure the mitochondrial oxygen consumption rate (OCR) and the extracellular acidification rate (ECAR), indicators of mitochondrial OXPHOS and glycolytic function, respectively.

**Figure 4:**
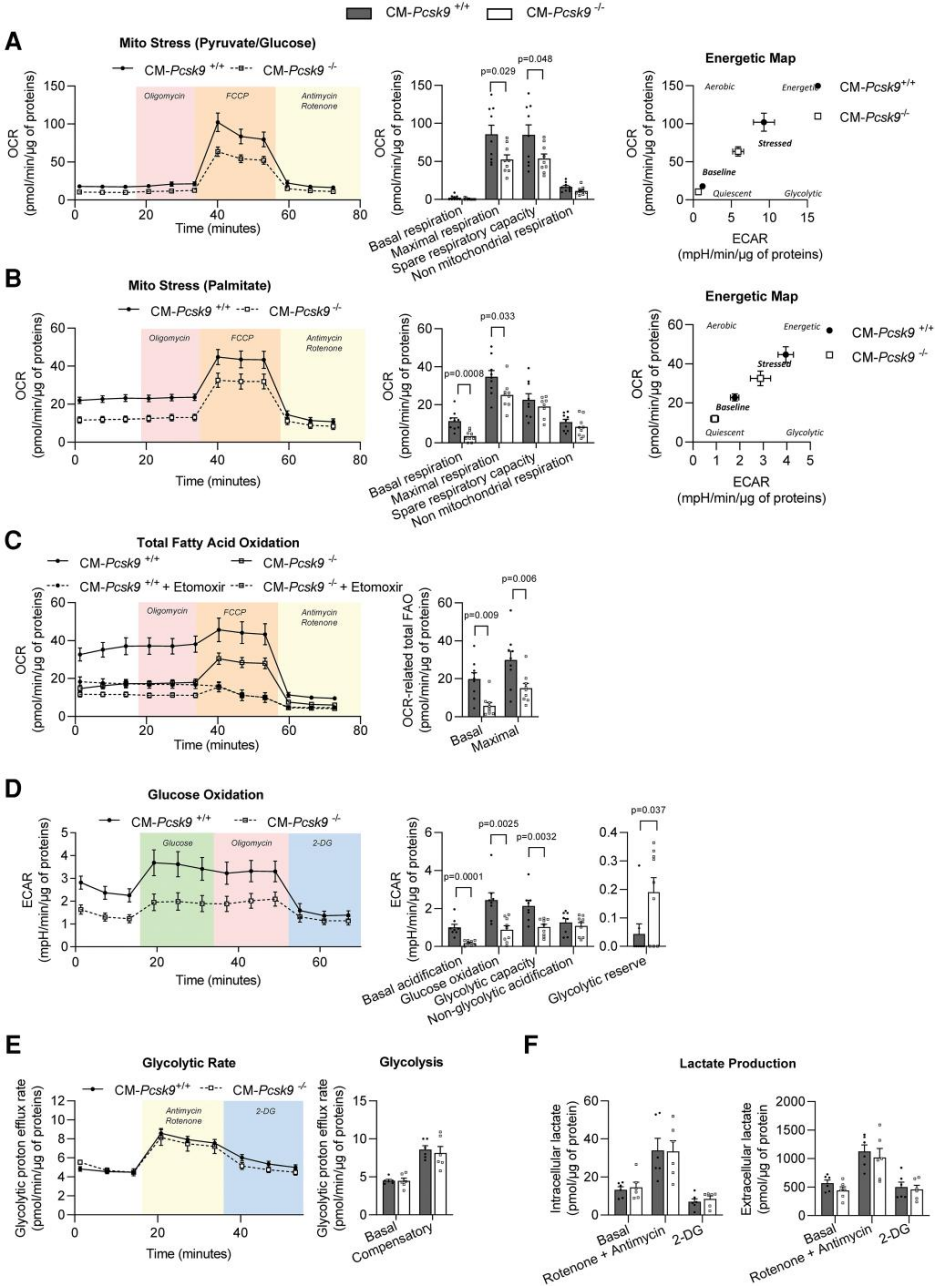
Impairment of mitochondrial but not glycolytic function in CM*-Pcsk9*^−/−^ cardiomyocytes. OCR and ECAR were determined using the Seahorse flux analyser in primary cardiomyocytes isolated from 28-week-old CM*-Pcsk9*^+/+^ and CM*-Pcsk9*^−/−^ mice and normalized to total cellular protein content for each well (12 wells from *n* = 9). (*A* and *B*) OCR (left), quantification of respiratory parameters (middle), and energetic maps obtained by plotting ECAR and OCR values at baseline and under FCCP-induced stress (right) of CM*-Pcsk9*^+/+^ and CM*-Pcsk9*^−/−^ cardiomyocytes using pyruvate/glucose (*A*) or palmitate (*B*) as the substrate. OCR was measured under basal conditions and after addition of oligomycin (1 µM) to inhibit ATP synthase, FCCP (1 µM) to uncouple oxidative phosphorylation, and antimycin A (2 µM) and rotenone (2 µM) to gauge non-mitochondrial respiration. (*C*) Left: measurement of OCR-related fatty acid oxidation (FAO) in cardiomyocytes pre-treated with etomoxir (CPT1B inhibitor, 100 μM, 15 min) before palmitate addition (150 μM) at time 0. Right: quantification of total (endogenous + exogenous) fatty acid oxidation at baseline and under FCCP-induced stress (maximal). See Supplementary material online, *Figure S3A*. (*D*) ECAR (left) and quantification of glycolytic parameters (right) in glucose-deprived cardiomyocytes after addition of glucose (10 mM) to fuel glycolysis and OXPHOS, oligomycin (2 μM) to inhibit ATP synthase, and 2-deoxyglucose (2-DG) (100 mM) to inhibit glucose catabolism. (*E*) Glycolytic proton efflux rate and quantification of glycolysis in cardiomyocytes after addition of 0.5 μM rotenone/antimycin A and 2-deoxyglucose (2-DG) (100 mM). (*F*) Intracellular and extracellular lactate levels in primary cardiomyocytes isolated from 28-week-old CM*-Pcsk9*^+/+^ and CM*-Pcsk9*^−/−^ mice (*n* = 6). Lactate concentrations were measured under basal conditions and after addition of 2 µM rotenone/antimycin and 2-deoxyglucose (2-DG) (100 mM), and normalized to total protein content. Data are mean ± SEM. *P* values are shown vs. CM*-Pcsk9*^+/+^ by two-tailed *t*-test (*A–E*) and two-way ANOVA (*F*).

We first used the Mito Stress test to assess mitochondrial function under basal and stress conditions in cardiomyocytes isolated from 28-week-old mice. Of note, addition of the ATP synthase inhibitor oligomycin had no effect on the OCR, which is likely due to the quiescent state (and thus low energy demand) of adult primary cardiomyocytes in culture.^21^ Therefore, proton leak and ATP production coupled to respiration could not be determined in this experiment. Expression of the αMHC-Cre transgene alone did not affect mitochondrial function or the bioenergetic profile under stress (see Supplementary material online, *Figure S3A*). We did not observe a significant reduction in basal OCR in CM*-Pcsk9*^−/−^ cardiomyocytes when glucose/pyruvate was the substrate (*Figure 4A*), indicating that mitochondrial function was maintained under these conditions. However, basal OCR was lower in CM*-Pcsk9*^−/−^ cardiomyocytes when palmitate was the substrate (*Figure 4B*), consistent with the downregulation of genes and proteins related to fatty acid metabolism (*Figure 4B–D*). Maximal OCR (following addition of carbonyl cyanide 4-(trifluoromethoxy)phenylhydrazone [FCCP] to increase energy demand, i.e. in stress conditions) was reduced in CM*-Pcsk9*^−/−^ cardiomyocytes when either palmitate or pyruvate/glucose was substrate (*Figure4A* and *B*). In addition, basal and maximal OCR-related endogenous and exogenous fatty acid oxidation (FAO) was reduced in CM*-Pcsk9*^−/−^ cardiomyocytes (*Figure 4C* and see Supplementary material online, *Figure S3B*). Importantly, with glucose/pyruvate or palmitate as substrate, CM*-Pcsk9*^−/−^ cardiomyocytes had a lower oxidative (OCR) and glycolytic (ECAR) bioenergetic profile under stress (*Figure4A* and *B*), indicating energetic impairmentCM*-Pcsk9*. Similar results were observed (with glucose/pyruvate as substrate) in cardiomyocytes from 10-week-old CM*-Pcsk9*^−/−^ mice (see Supplementary material online, *Figure S3C*), confirming an early onset of mitochondrial dysfunction induced by *Pcsk9* deletion.

By measuring ECAR in glucose-starved cells to which saturating amounts of glucose were added (to fuel glycolysis and OXPHOS), followed by addition of oligomycin (to inhibit mitochondrial ATP synthesis) and 2-deoxyglucose (to inhibit glucose catabolism), we showed that CM*-Pcsk9^−/−^* cardiomyocytes had lower levels of basal acidification, glucose oxidation and glycolytic capacity but a higher glycolytic reserve (as shown by a slightly higher ECAR after oligomycin addition) (*Figure 4D*). Given that mitochondrial activity (and not only glycolysis) contributes to extracellular acidification in cardiomyocytes, we also calculated the glycolytic proton efflux rate (by subtracting the contribution of mitochondrial CO_2_ from the total proton efflux rate), which correlates directly with lactate production and is a more specific measure of glycolysis than ECAR alone. We showed that basal and compensatory glycolysis rates (measured after mitochondrial inhibition) were unchanged by cardiac *Pcsk9* deficiency (*Figure 4E*), indicating the inability of cells to switch to glycolysis to meet energy demands when mitochondrial respiration is blocked. Consistently, we also showed that extracellular and intracellular lactate levels were not altered by *Pcsk9* deficiency in cardiomyocytes either under basal conditions or after mitochondrial inhibition (*Figure 4F*).

Together these results provide further evidence for impairments in mitochondrial but not glycolytic function in CM*-Pcsk9*^−/−^ cardiomyocytes.

### 3.4 Mitochondrial ETC complexes are altered in CM*-Pcsk9^−/−^* mice

**Figure 5:**
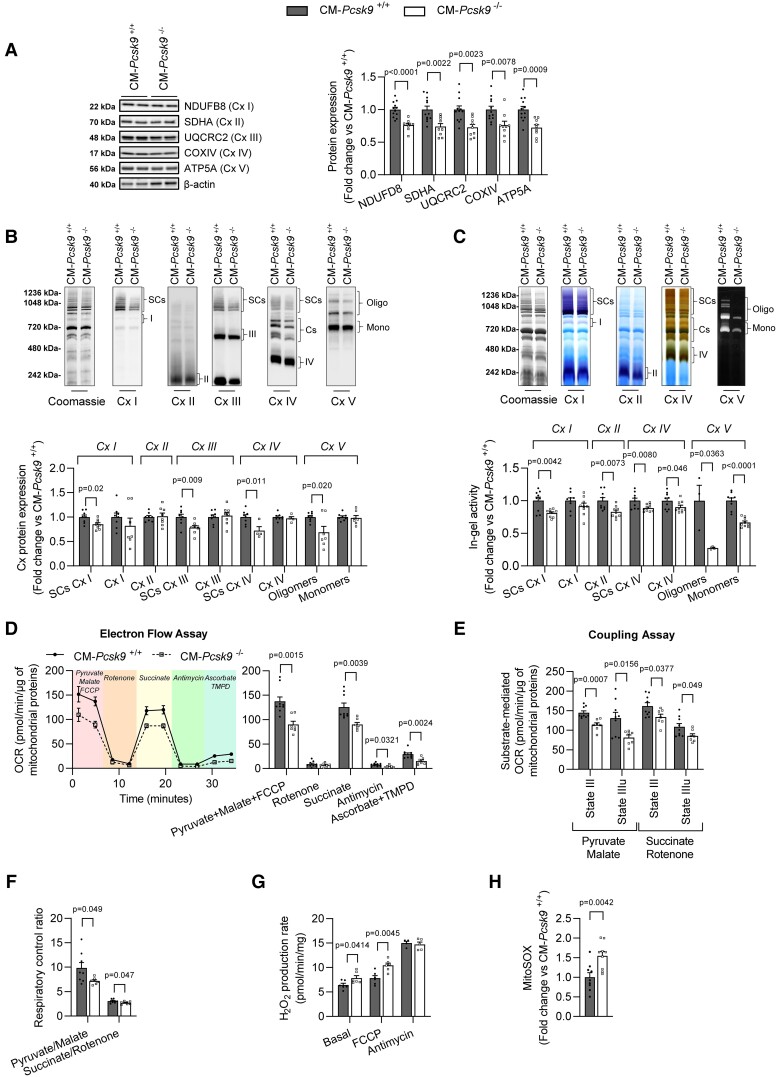
Mitochondrial OXPHOS complexes are altered in CM*-Pcsk9*^−/−^ mice. (*A*) Representative immunoblots (after SDS electrophoresis) with antibodies against subunits of mitochondrial ETC complexes: NDUFB8 [complex (Cx) *I*], SDHA (Cx II), UQCRC2 (Cx III), COXIV (Cx IV) and ATP5A (Cx V) (left) and quantification (right) in 28-week-old CM*-Pcsk9*^+/+^ and CM*-Pcsk9*^−/−^ cardiomyocytes (*n* = 12). β-actin served as loading control. (*B*) Representative immunoblots (after blue native polyacrylamide gel electrophoresis) (top) and quantification (bottom) of supercomplexes (SCs), individual ETC complexes, and Complex V oligomers/monomers in cardiac mitochondria from 28-week-old CM*-Pcsk9*^+/+^ and CM*-Pcsk9*^−/−^ mice (*n* = 8). (*C*) Representative images (top) and quantification (bottom) of in-gel activity of SCs, individual ETC complexes and Complex V oligomers/monomers in cardiac mitochondria from 28-week-old CM*-Pcsk9*^+/+^ and CM*-Pcsk9*^−/−^ mice (*n* = 9). Activity was normalized to total mitochondrial protein (Coomassie gel) and to that of CM*-Pcsk9*^+/+^. (*D–F*) OCR was determined using the Seahorse flux analyser in mitochondria isolated from 28-week-old CM*-Pcsk9*^+/+^ and CM*-Pcsk9*^−/−^ mice hearts and normalized to total mitochondrial protein content for each well (12 wells from *n* = 9). (*D*) Measurement (left) and quantification (right) of OCR determined by electron flow assay using pyruvate/malate/FCCP (10 mM/5 mM/4 µM) to drive Complex I-dependent respiration in an uncoupled state, rotenone (2 µM) to inhibit Complex I, succinate (5 mM) to drive Complex II-dependent respiration, antimycin A (4 µM) to inhibit Complex III, and ascorbate/TMPD (10 mM/0.1 mM) to drive Complex IV-dependent respiration in cardiac mitochondria from 28-week-old CM*-Pcsk9*^+/+^ and CM*-Pcsk9*^−/−^ mice. (*E*) Quantification of State III and State IIIu respiration and (*F*) respiratory control ratio (State III/State IV) determined in a coupling assay in cardiac mitochondria from 28-week-old CM*-Pcsk9*^+/+^ and CM*-Pcsk9*^−/−^ mice. Pyruvate/malate (10 mM/5 mM) and succinate/rotenone (5 mM/2 µM) were used to drive Complex I- and Complex II-dependent respiration, respectively. Respiration was initiated by adding ADP (5 mM, State III) and stopped by adding oligomycin (2.5 µM, State IV). FCCP (4 µM) dissipated mitochondrial membrane potential and initiated respiration (State IIIu). See Supplementary material online, *Figure S4B* and *C*. (*G*) Mitochondrial levels of reactive oxygen species in cardiac mitochondria from 28-week-old CM*-Pcsk9*^+/+^ and CM*-Pcsk9*^−/−^ mice (*n* = 6), determined with Amplex Red assay at baseline or under stress induced by FCCP (4 µM). Antimycin A (4 µM) was used as a positive control of massive ROS production. (*H*) Mitochondrial levels of reactive oxygen species in 28-week-old CM*-Pcsk9*^+/+^ and CM*-Pcsk9*^−/−^ cardiomyocytes (*n* = 9), determined with MitoSOX Red probe. Values are mean ± SEM. *P* values are shown vs. CM*-Pcsk9*^+/+^ by two-tailed *t*-test.

Given that bioenergetics analyses showed impaired mitochondrial function in CM*-Pcsk9*^−/−^ cardiomyocytes, we investigated the effect of *Pcsk9* deficiency on the mitochondrial electron transport chain (ETC) complexes that drive OXPHOS. Immunoblot analysis using antibodies against NDUFB8 (Complex I), SDHA (Complex II), UQCRC2 (Complex III) and COXIV (Complex IV), and ATP5A (Complex V) showed that levels of these proteins were lower in CM*-Pcsk9*^−/−^ cardiomyocytes (*Figure 5A*). We next investigated these structures in intact isolated cardiac mitochondria and showed that: (i) Complexes I, III, and IV, but not Complex II, were assembled into SCs, and Complex V (ATP synthase) formed oligomers; (ii) *Pcsk9* deficiency reduced the protein levels of the SCs and the Complex V oligomers; and (iii) *Pcsk9* deficiency did not reduce the abundance of individual complexes, but reduced the percentages of SCs containing Complexes III and IV (*Figure 5B* and see Supplementary material online*Figure S4A*). In-gel activity analysis (in the presence of substrates for each respective complex) revealed reduced activity of Complex I- and Complex VI-containing SCs and Complexes II and IV and strongly reduced activity of monomeric and oligomeric Complex V in CM*-Pcsk9^−/−^* mitochondria (*Figure 5C*).

We also assessed ETC efficiency of isolated mitochondria using the Seahorse flux analyser. First, we evaluated the sequential electron flow through the complexes and showed that isolated CM*-Pcsk9^−/−^* mitochondria exhibited lower rates of Complex I-, II-, and IV-dependent mitochondrial respiration (*Figure 5D*), indicating a lower efficiency of ETC at multiple sites. Secondly, we evaluated the degree of coupling between the ETC and the OXPHOS machinery. State III respiration was initiated by ADP and stopped by oligomycin (State IV). FCCP was used to depolarize the mitochondria membrane potential and restart the respiration (State IIIu) (see Supplementary material online, *Figure S4B* and *C*). In the presence of pyruvate/malate or succinate/rotenone (Complex I and II substrates, respectively), OCRs after addition of ADP (State III) or FCCP (State IIIu) and the respiratory control ratios (State III/State IV) were significantly lower in CM*-Pcsk9^−/−^* mitochondria (*Figure5E* and *F*, see Supplementary material online, *Figure S4B* and *C*), again indicating a lower efficiency of mitochondrial energy metabolism. Consistent with a defect in the ETC, H_2_O_2_ production rate was higher in CM*-Pcsk9*^−/−^ mitochondria both at baseline and following addition of FCCP to increase energy demand (*Figure 5G*). In agreement, *Pcsk9* deficiency also increased mitochondrial production of reactive oxygen species in isolated cardiomyocytes (*Figure 5H*).

Taken together, these data suggest that cardiac *Pcsk9* deficiency promotes structural and functional alterations of the ETC complexes, resulting in reduced capacity of the mitochondria and impaired energy metabolism.

### 3.5 Lipid composition, proximity with ER, and morphology of mitochondria are altered in CM*-Pcsk9^−/−^* mice

**Figure 6:**
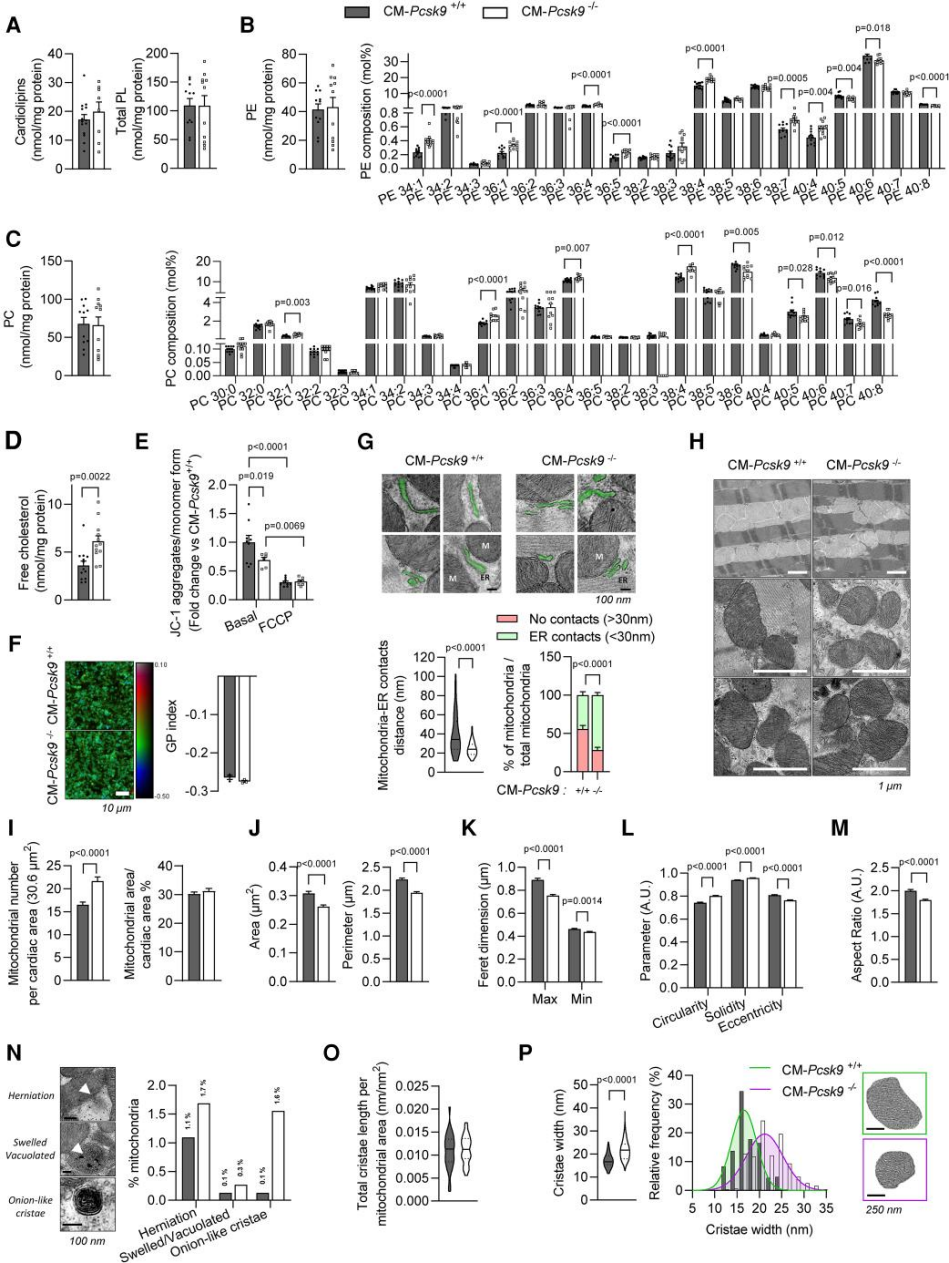
Lipid composition, proximity with ER and morphology of mitochondria are altered in CM*-Pcsk9*^−/−^ mice. (*A*) Cardiolipin and total phospholipid levels, (*B*) PE levels and composition, (*C*) PC levels and composition, and (*D*) free cholesterol levels in membranes of cardiac mitochondria from 28-week-old CM*-Pcsk9*^+/+^ and CM*-Pcsk9*^−/−^ mice (*n* = 11). (*E*) Quantification of mitochondrial membrane potential (ΔΨm) at baseline or under stress induced by FCCP (1 µM; 10 min; positive control of ΔΨm disruption) in isolated cardiac mitochondria from 28-week-old CM*-Pcsk9*^+/+^ and CM*-Pcsk9*^−/−^ mice (*n* = 7–9). (*F*) Pseudocolour images showing the Laurdan dye generalized polarization (GP) index at each pixel position and average GP index from 10 to 15 images in isolated cardiac mitochondria from 28-week-old CM*-Pcsk9*^+/+^ and CM*-Pcsk9*^−/−^ mice (*n* = 3). Red: rigid; blue: fluid. Scale, 10 µm. (*G*) Representative TEM images (top) and quantification of distance between mitochondria and ER (bottom, left panel) and of the percentage of mitochondria having contact (<30 nm) with the ER (bottom, right panel) in 28-week-old CM*-Pcsk9*^−/−^ vs. CM*-Pcsk9*^+/+^ hearts (≥100 junctions from ≥12 images; *n* = 3). Scale, 100 nm. (*H*) Representative TEM images of mitochondrial ultrastructure and quantification of (*I*) mitochondrial number and mitochondrial area per cardiac area (≥1480 mitochondria for >67 images) and (*J–M*) individual mitochondrial area/perimeter and mitochondrial shape descriptors (≥782 mitochondria for >67 images) in LV tissue from 28-week-old CM*-Pcsk9*^+/+^ and CM*-Pcsk9*^−/−^ mice (*n* = 3). AU, arbitrary units. Scale, 1 µm. (*N*) Representative TEM images and percentage of indicated mitochondrial features (≥1480 mitochondria from >67 images) in LV tissue from 28-week-old CM*-Pcsk9*^+/+^ and CM*-Pcsk9*^−/−^ mice (*n* = 3). Scale, 100 nm. (*O*) Cristae density (length per mitochondrial area) (30 mitochondria) and (*P*) quantification (left) and distribution (right) of cristae width (≥300 cristae) in LV tissue from 28-week-old CM*-Pcsk9*^+/+^ and CM*-Pcsk9*^−/−^ mice (*n* = 3). Scale, 250 nm. Data are mean ± SEM. *P* values are shown in the figure vs. CM*-Pcsk9*^+/+^ by two-tailed *t*-test (*A–D*, *F*, *G*, *I–M*, *O*, *P*) or two-way ANOVA (*E*).

Since PCSK9 is a master regulator of lipid homeostasis, we hypothesized that alterations in the lipid content of mitochondria could contribute to the impaired mitochondrial function in CM*-Pcsk9^−/−^* mice. We observed that *Pcsk9* deficiency did not affect levels of cardiolipin, total phospholipids, phosphatidylethanolamine (PE), and phosphatidylcholine (PC), but promoted alterations in the fatty acyl chains of PC and PE and an increase in free cholesterol (*Figure 6A–D*). As expected, given the altered lipid composition, the mitochondrial membrane potential was lower in CM*-Pcsk9^−/−^* mitochondria (*Figure 6E*). However, the mitochondrial fluidity was not altered by *Pcsk9* deficiency (*Figure 6F*).

Most of the lipids for mitochondrial membranes are produced in the endoplasmic reticulum (ER), prompting us to investigate whether *Pcsk9* deficiency alters the proximity between mitochondrial membranes and the ER. TEM showed that 28-week-old CM*-Pcsk9*^−/−^ mouse hearts had a higher percentage of mitochondria <30 nm from the ER (*Figure 6G*). TEM also showed that mitochondria from 28-week-old CM*-Pcsk9*^−/−^ mouse hearts had cytoarchitectural aberrations such as mitochondrial fragmentation (higher mitochondrial number/cardiac area but no change in mitochondrial area/cardiac area) (*Figure 6H* and *I*). CM*-Pcsk9*^−/−^ mitochondria were smaller than CM*-Pcsk9^+/+^* mitochondria (*Figure 6J*) and their maximum and minimum Feret’s diameters (indicating distance between two points) were lower (*Figure 6K*), indicating changes in mitochondrial shape. CM*-Pcsk9*^−/−^ mitochondria were less branched and more circular (*Figure 6L*) and had a low aspect ratio (indicating elongation) (*Figure 6M*), suggesting altered fusion/fission processes. Further, *Pcsk9* deletion increased the percentage of swollen, vacuolated, and herniated mitochondria (*Figure 6N*). We also noted that the cristae density of CM*-Pcsk9*^−/−^ mitochondria was unchanged (*Figure 6O*), but most CM*-Pcsk9*^−/−^ cristae were wider, highly folded (Z-like), and disorganized (*Figure 6P*), and some had an aberrant onion-like structure (*Figure 6N*).

Results from the transcriptomic analysis indicated that several processes involved in mitochondrial DNA transcription/translation/replication, protein import, and quality control were affected in CM*-Pcsk9*^−/−^ mouse hearts, consistent with defective mitochondrial biogenesis (see Supplementary material online, *Figure S5A*). Similarly, we observed decreased expression of genes related to mitochondrial function (*Gfm2*, *Ndufv1*, *Nubpl*, and *Tmlhe*) in CM*-Pcsk9*^−/−^ hearts (see Supplementary material online, *Figure S5B*) and decreased protein levels of markers of mitochondrial biogenesis (PGC1α), fission (DRP1), and fusion (MFN2) in cardiomyocytes isolated from CM*-Pcsk9*^−/−^ hearts (see Supplementary material online, *Figure S5C*).

### 3.6 *Pcsk9* silencing causes a shift to glycolytic metabolism, compromises the ability to meet increased energy demand and promotes a hypertrophic phenotype in cardiomyocytes

To investigate the effect of acute *Pcsk9* silencing in a cardiomyocyte cell line model, we differentiated H9c2 cardiomyoblasts into adult cardiomyocyte-like cells by treatment with retinoic acid. The cardiac-like phenotype was confirmed by higher levels of transcripts and proteins involved in calcium handling (see Supplementary material online, *Figure S6A* and *B*) and increased sensitivity to hypoxia-induced cell death (see Supplementary material online, *Figure S6C*).^22^ PCSK9 levels were twice as high in H9c2 differentiated into adult cardiomyocyte-like cells than in H9c2 cardiomyoblasts but, as expected, lower than in human HepG2 liver cells (see Supplementary material online, *Figure S6D–F*). Transfection with siRNA was highly efficient at reducing mRNA and protein levels in both H9c2 cardiomyoblasts and cardiomyocytes (see Supplementary material online, *Figure S6D* and *E*).

**Figure 7:**
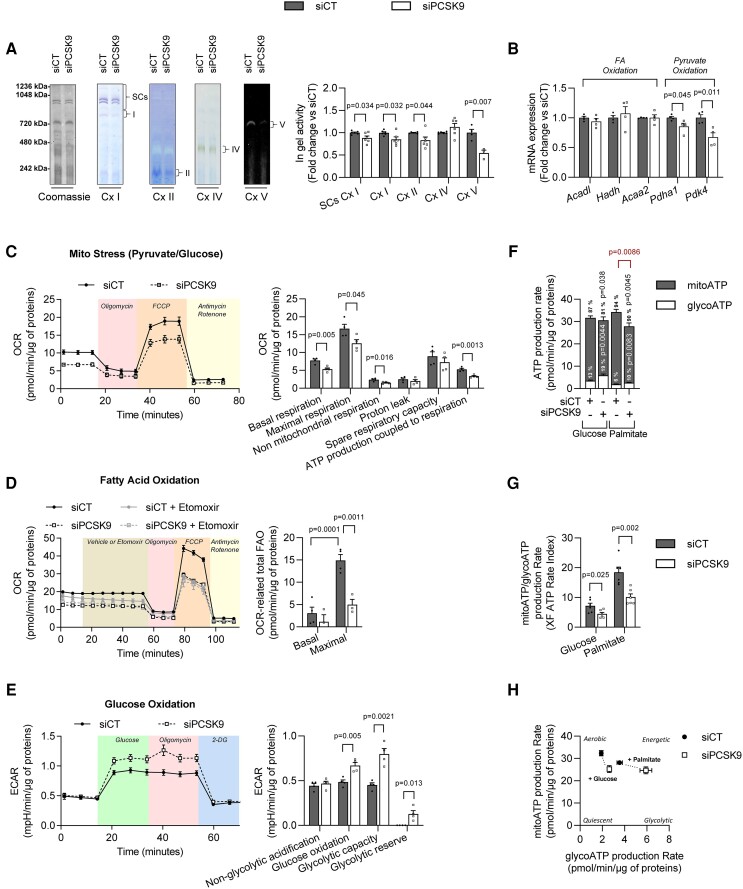
Acute *Pcsk9* silencing causes a shift to glycolytic metabolism in cardiomyocytes and compromises ability to meet increased energy demand. H9c2 cardiomyocytes treated with siRNA against *Pcsk9* (siPCSK9) or scrambled control (siCT) for 24 h were used for all experiments. (*A*) Representative gels (left) and quantification (right) of in-gel activity of ETC complexes in mitochondria from *Pcsk9*-deficient H9c2 cardiomyocytes (*n* = 4–6). Activity was normalized to total mitochondria protein (Coomassie gel) and to cells treated with scrambled control. (*B*) mRNA expression of *Acadl, Hadh, Acaa2, Pdha1*, and *Pdk4* in *Pcsk9*-deficient H9c2 cardiomyocytes and in cells treated with scrambled control (*n* = 4). (*C–H*) OCRs and ECARs were determined using the Seahorse flux analyser and ATP production was determined using the Seahorse XF real-time ATP rate assay in H9c2 cardiomyocytes treated with siPCSK9 or scrambled control; values were normalized to total cellular protein content for each well (12 wells from 4 to 6 independent experiments). (*C*) OCRs and quantification of respiratory and ATP production parameters in H9c2 cardiomyocytes using pyruvate/glucose as substrate, oligomycin (1 µM) to inhibit ATP synthase, FCCP (5 µM) to uncouple oxidative phosphorylation, and antimycin A (1 µM) and rotenone (1 µM) to gauge non-mitochondrial respiration. (*D*) Left: OCR-related fatty acid oxidation (FAO) in H9c2 cardiomyocytes pre-treated with etomoxir (40 μM, 15 min) before addition of palmitate (150 μM) at time 0. Right: Quantification of total (endogenous + exogenous) fatty acid oxidation at baseline and under FCCP-induced stress. (*E*) ECARs and quantification of glycolytic parameters in glucose-deprived H9c2 cardiomyocytes after addition of glucose (10 mM) to fuel glycolysis and OXPHOS, oligomycin (2 μM) to inhibit ATP synthase, and 2-deoxyglucose (2-DG) (50 mM) to inhibit glucose catabolism. (*F*) Metabolic flux analysis showing quantification of mitochondrial (mitoATP) and glycolytic (glycoATP) ATP production in H9c2 cardiomyocytes. (*G*) ATP rate index indicating the changes in metabolic phenotype, calculated from data in (*F*). An increase in this index indicates a more oxidative/less glycolytic phenotype. (*H*) Energetic map of mitoATP vs. glycoATP of H9c2 cardiomyocytes using carbohydrates (glucose) or fatty acids (palmitate) as substrate [from (*F*)]. Values are mean ± SEM. *P* values are shown vs. CM*-Pcsk9*^+/+^ by *t*-test (*A–C*, *E–G*) and two-way ANOVA (*D*).

In-gel activity in the presence of substrates for the respective complexes showed reduced activity of individual complexes (for I, II, and V) and Complex I-containing SCs in mitochondria from *Pcsk9*-deficient H9c2 cardiomyocytes (*Figure 7A*), supporting impaired mitochondrial function in response to acute *Pcsk9* silencing. Acute *Pcsk9* silencing did not reduce expression of genes related to FAO but reduced the expression of *Pdha1* and *Pdk4*, genes involved in pyruvate oxidation (*Figure 7B*). By using the Seahorse Mito Stress test with glucose/pyruvate as substrate to assess mitochondrial function, we showed that *Pcsk9* silencing reduced basal and maximal respiration and ATP production coupled to respiration (*Figure 7C*). Maximal but not basal OCR-related total FAO was lower in *Pcsk9*-deficient H9c2 cardiomyocytes (*Figure 7D*), indicating that FAO is only reduced under stress conditions. We also showed that glucose oxidation, glycolytic capacity and glycolytic reserve were higher in *Pcsk9*-deficient H9c2 cardiomyocytes (*Figure 7E*), consistent with more glycolytic energy metabolism in these cells.

By simultaneously quantifying glycolytic and mitochondrial ATP production rates with the Seahorse ATP real-time rate assay (*Figure 7F–H* and see Supplementary material online, *Figure S6G*), we showed that *Pcsk9*-deficient H9c2 cardiomyocytes had a higher proportion of ATP generated by glycolysis and a smaller proportion of ATP generated by OXPHOS (with either glucose or palmitate as substrate) (*Figure 7F*). In addition, the total ATP production rate was significantly lower in *Pcsk9*-deficient H9c2 cardiomyocytes when cultured in palmitate but not in glucose (*Figure 7F*). The overall energetic rate was lower in *Pcsk9*-deficient cells with either substrate (*Figure 7G*), indicating a less oxidative and more glycolytic phenotype (*Figure 7H*).

We also investigated the impact of *Pcsk9* silencing on the expression of hypertrophy-associated transcripts in H9c2 cardiomyocytes at two timepoints (after 24 h, as used in all other experiments, and 72 h of siRNA transfection). *Pcsk9* silencing did not affect *Nppb* expression at either timepoint but resulted in a small decrease in *Nppa* expression at 24 h and a large increase at 72 h (see Supplementary material online, *Figure S6H*). Furthermore, *Pcsk9* silencing increased the *Myh7/Myh6* ratio at 24 h and to a greater extent at 72 h (see Supplementary material online, *Figure S6I*). These results are consistent with a progressive onset of cardiomyocyte hypertrophy and reinforce the causal link between metabolic changes and structural remodelling observed in CM*-Pcsk9*^−/−^ mice.

Taken together, these results show that acute *Pcsk9* silencing impairs the production of OXPHOS-derived ATP, causing cardiomyocytes to rely on glycolytic pathways to maintain their energy and to have an overall lower rate of metabolic flux, and promotes a hypertrophic phenotype.

### 3.7 *Pcsk9* overexpression promotes a more oxidative phenotype in cardiomyocytes

To confirm the importance of cardiac PCSK9 in mitochondrial function, we transfected mature H9c2 cardiomyocytes with PCSK9-GFP cDNA to overexpress *Pcsk9*. By using the Seahorse Mito Stress test with glucose/pyruvate as substrate, we showed that *Pcsk9* overexpression increased basal and maximal respiration and ATP production coupled to respiration (see Supplementary material online, *Figure S7A*). We also showed that *Pcsk9* overexpression increased FAO (see Supplementary material online, *Figure S7B* and *C*) without modification of glycolytic metabolism (see Supplementary material online, *Figure S7D*). By using the Seahorse ATP real-time rate assay, we showed that *Pcsk9* overexpression resulted in a higher proportion of ATP generated by OXPHOS with glucose as substrate (see Supplementary material online, *Figure S7E*). In addition, the overall energetic rate was higher in *Pcsk9*-overexpressing H9c2 cardiomyocytes with palmitate and unchanged with glucose (see Supplementary material online, *Figure S7F*), indicating that *Pcsk9* overexpression promotes a more oxidative phenotype (see Supplementary material online, *Figure S7G*).

### 3.8 Cardiomyocyte-secreted PCSK9 does not reverse mitochondrial dysfunction induced by *Pcsk9* silencing

**Figure 8:**
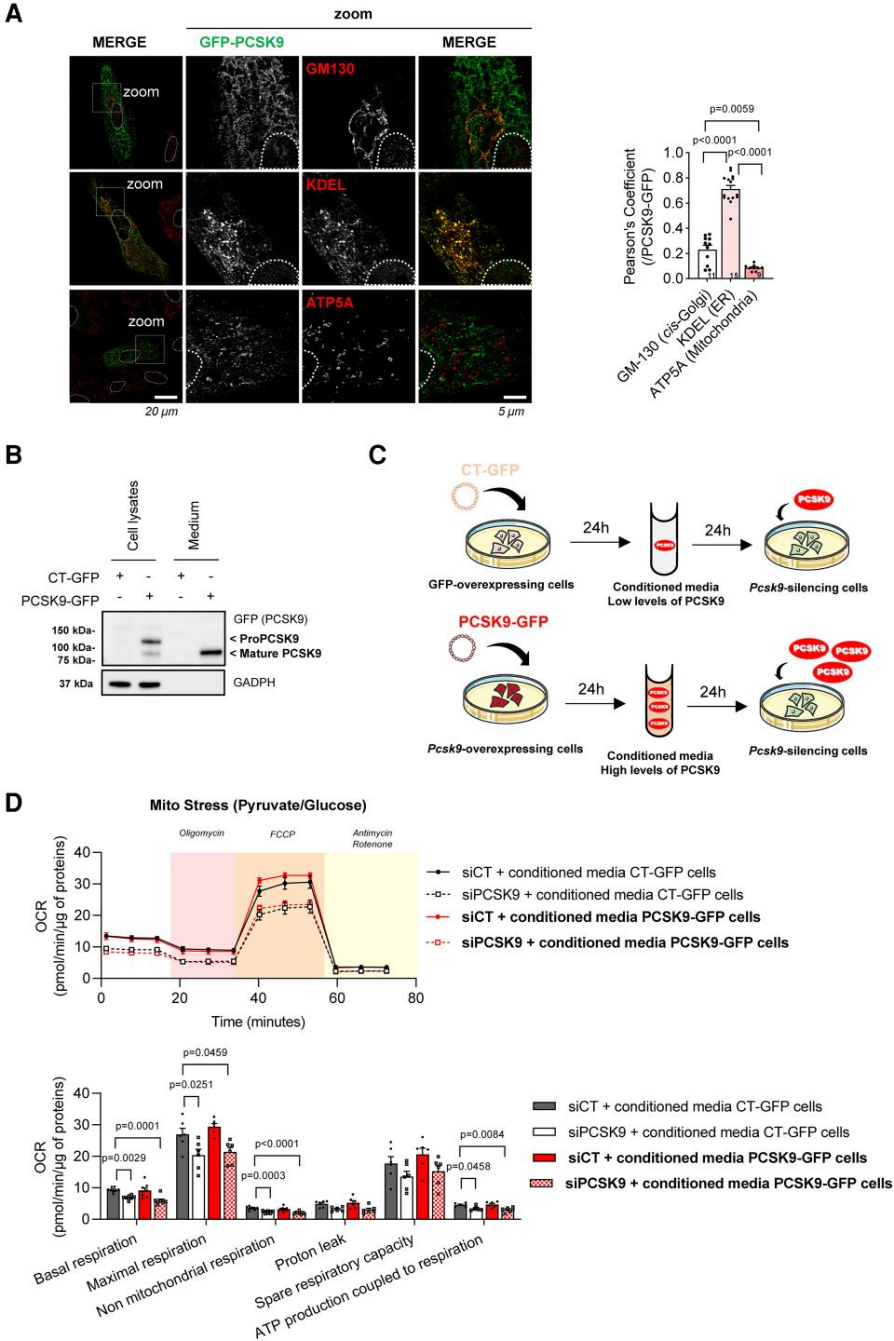
Cardiomyocyte-secreted PCSK9 does not reverse mitochondrial dysfunction induced by *Pcsk9* silencing. (*A*) Representative confocal images (left) and quantification (right) of the subcellular localization of PCSK9-GFP (green) and markers for Golgi apparatus (Gm130), endoplasmic reticulum (KDEL) and mitochondria (ATP5A) (all red) in H9c2 cardiomyocytes transfected with PCSK9-GFP or empty control (CT-GFP) for 24 h. Co-localization was analysed on a minimum of 5 fields of view (6710 μm^2^) each containing approximately 9–15 cells from 3 independent experiments. Scale, 20 and 5 µm. (*B*) Immunoblot of PCSK9 in H9c2 cardiomyocytes transfected with PCSK9-GFP or CT-GFP for 24 h. Intracellular and secreted PCSK9 was detected using anti-GFP in cell lysates and media (at a concentration proportional to the amount of cell lysate deposit). GAPDH was used as a loading and cells control (*n* = 4). (*C* and *D*) Conditioned media from H9c2 cardiomyocytes transfected with PCSK9-GFP or CT-GFP for 24 h was used to treat H9c2 cardiomyocytes previously transfected with siRNA against *Pcsk9* (siPCSK9) or scrambled control (siCT) for 24 h. After 24 h incubation, OCRs were determined using the Seahorse flux analyser in the media-treated H9c2 cardiomyocytes. Values were normalized to total cellular protein content for each well (12 wells from 6 to 8 independent experiments). (*C*) Experimental scheme. (*D*) OCRs and quantification of respiratory and ATP production parameters in H9c2 cardiomyocytes using pyruvate/glucose as substrate, oligomycin (1 µM) to inhibit ATP synthase, FCCP (5 µM) to uncouple oxidative phosphorylation, and antimycin A (1 µM) and rotenone (1 µM) to gauge non-mitochondrial respiration. Values are mean ± SEM. *P* values are shown vs. indicated condition by one-way ANOVA followed by Tukey’s post-test.

To determine the subcellular localization of PCSK9 in cardiomyocytes, we investigated whether PCSK9 co-localized with markers for mitochondria (ATP5A), endoplasmic reticulum (KDEL), and Golgi apparatus (Gm130) in mature H9c2 cardiomyocytes transfected with PCSK9-GFP cDNA. We showed that the majority of the PCSK9 was located in the ER, a small part was localized in the Golgi apparatus, and none was detected in the mitochondria (*Figure 8A*). These results suggest that cardiac PCSK9 is located in the ER and a fraction is transported to the Golgi apparatus before being secreted. In agreement, we detected both forms of PCSK9-GFP (pro and mature) in cell lysates and only the mature (secreted) form in corresponding media (*Figure 8B*).

To determine whether cardiomyocyte-secreted PCSK9 could reverse the mitochondrial dysfunction induced by *Pcsk9* silencing, we treated *Pcsk9-*deficient H9c2 cardiomyocytes with conditioned media from H9c2 cardiomyocytes transfected with PCSK9-GFP or empty control (CT-GFP) (i.e. with high and low levels of secreted PCSK9, respectively) for 24 h (*Figure8B* and *C*). By using the Seahorse Mito Stress test with glucose/pyruvate as substrate to assess mitochondrial function, we showed that secreted PCSK9 did not prevent the decrease of basal and maximal respiration and ATP production coupled to respiration induced by *Pcsk9* silencing (*Figure 8D*), suggesting that those effects were not mediated by secreted PCSK9.

## 4. Discussion

In this study, we showed that cardiomyocyte-specific deletion of *Pcsk9* in mice caused a progressive dilated cardiomyopathy-like phenotype characterized by cardiomyocyte hypertrophy, interstitial fibrosis, and contractile dysfunction, culminating in heart failure and premature death. We further showed that *Pcsk9* deficiency in mouse hearts resulted in impaired mitochondrial metabolism, reduced activity of mitochondrial ETC complexes, and a shift towards increased dependence on glycolytic pathways that was insufficient to meet energy demand under stress conditions. Circulating levels of lipids were unchanged in CM*-Pcsk9*^−/−^ mice, but the lipid composition of mitochondrial membranes was altered. In addition, cardiomyocytes from CM*-Pcsk9*^−/−^ mice had an increased number of mitochondria–ER contacts and alterations in the morphology of mitochondrial cristae, with potential implications for the assembly and activity of the mitochondrial ETC complexes. Importantly, we also showed that acute *Pcsk9* silencing reduced the activity of mitochondrial ETC complexes and impaired mitochondrial metabolism, whereas *Pcsk9* overexpression increased mitochondrial function in a cell line model of adult cardiomyocyte-like cells. Finally, we showed that the majority of PCSK9 was localized to the ER and that cardiomyocyte-secreted PCSK9 did not reverse the mitochondrial dysfunction induced by *Pcsk9* silencing.

The absolute quantity of proteins in a cell ranges widely, from one copy to ten million copies.^23^ Because of technical limitations, many low-abundant proteins cannot be detected by standard technologies. This is unfortunate as emerging data indicate that low-abundant proteins may have important biological functions.^23^ Previous studies have shown that PCSK9 protein is present, albeit at very low levels, in rat^6,24^ and mouse primary cardiomyocytes.^11^ Here, we confirmed expression of this low-abundant protein in mouse primary cardiomyocytes (*Figure 1F*) and showed that PCSK9 is also expressed in human primary cardiomyocytes (see Supplementary material online, *Figure S8*).

Da Dalt *et al.*^10^ recently reported using whole-body *Pcsk9*-deficient mice that *Pcsk9* deficiency results in heart failure and impairs cardiac lipid metabolism. We chose instead to develop cardiomyocyte-specific *Pcsk9*-deficient mice given that global *Pcsk9* knockout, which reduces both hepatic and non-hepatic *Pcsk9* expression, has several limitations. First, whole-body *Pcsk9* deficiency promotes systemic effects, including markedly reduced levels of plasma triglycerides and cholesterol.^10^ Secondly, hepatic-derived PCSK9 targets not only the LDLR but also the very low-density lipoprotein receptor (VLDLR); given that cardiac uptake of lipoproteins via the VLDLR promotes cardiac lipotoxicity in failing hearts,^14^ the absence of physiological regulation of VLDLR in combination with markedly altered plasma lipids is a severe confounding factor. Furthermore, the heart is composed of many cell types, the most abundant being cardiomyocytes, fibroblasts, endothelial cells, and peri-vascular cells.^25^ Both cardiomyocytes and non-cardiomyocytes respond to physiological and pathological stress, and maladaptive changes in non-cardiomyocytes participate in the pathogenesis of heart failure.^25^ As PCSK9 is expressed in many cell types, cardiomyocyte-specific *Pcsk9*-deficient mice are needed to clarify the specific role of PCSK9 for cardiac function. Finally, findings in whole-body *Pcsk9*-deficient mice have recently been challenged by results from tissue-specific knockout models. For example, a recent β-cell-specific *Pcsk9* knockout mouse model showed that β-cell function and glucose homeostasis are not impaired by *Pcsk9* deficiency,^26^ in contrast to findings from an earlier study using whole-body *Pcsk9*-deficient mice.^8^

Here, we used several approaches that consistently showed impaired metabolic flexibility and mitochondrial metabolism in *Pcsk9-*deficient cardiomyocytes. Under normal circumstances, fatty acids are the predominant substrate used for ATP production in the heart through mitochondrial oxidative metabolism. However, the cardiac metabolic network is highly flexible, and the heart can shift to using glycolysis as the primary energy source depending on, for example, substrate and oxygen availability and energy demand.^27^ Transcriptomics and targeted gene analysis of CM*-Pcsk9^−/−^* mouse hearts showed progressive downregulation of genes related to mitochondrial metabolism and increases or no changes in genes involved in non-mitochondrial metabolism. In agreement, we observed reduced levels of key proteins involved in FAO in CM*-Pcsk9^−/−^* hearts and a high acylcarnitine to free carnitine ratio in CM*-Pcsk9^−/−^* mitochondria, consistent with a low capacity for energy production.^27,28^ Bioenergetic analyses showed that mitochondrial but not glycolytic function was impaired in CM*-Pcsk9^−/−^* cardiomyocytes. We also observed structural and functional alterations of the ETC complexes and increased ROS production in mitochondria isolated from CM*-Pcsk9^−/−^* mouse hearts. Finally, analysis of adult cardiomyocyte-like cells confirmed that acute *Pcsk9* silencing reduced the activity of ETC complexes and impaired mitochondrial ATP production, causing cells to rely on glycolysis, whereas *Pcsk9* overexpression increased mitochondrial function. Taken together, our results extend those reported in whole-body *Pcsk9*-deficient mice^10^ by showing that lower levels of PCSK9 in cardiomyocytes may contribute to heart failure by reducing mitochondrial metabolism, which is not adequately compensated for by an increase in glycolysis.

The heart accounts for only ∼0.5% of body weight, but is responsible for roughly 8% of ATP consumption. Thus, the heart has a very high energy demand and is particularly sensitive to factors that affect the activity of the ETC complexes (I–IV) and ATP synthase (Complex V), key players in mitochondrial energy production. An earlier study using heart homogenates from whole-body *Pcsk9*-deficient mice reported that *Pcsk9* deficiency resulted in reduced abundance of Complexes I, II, and III but reduced activity only of Complex II.^10^ In contrast, we observed reduced abundance of integral proteins for Complexes I–IV and ATP synthase in CM*-Pcsk9^−/−^* cardiomyocytes. Recent evidence indicates that the ETC complexes are organized into SCs, which increase efficiency of the ETC and reduce oxidative damage.^29,30^ Impaired assembly of SCs is associated with heart failure.^31^ Here, we observed reduced assembly of SCs, reduced activity of all the complexes (or their SCs), and increased ROS production in mitochondria isolated from CM*-Pcsk9^−/−^* mouse hearts. By using a sequential electron flow assay, we confirmed that ETC efficiency was reduced at multiple sites. We also showed that *Pcsk9* deficiency reduced oligomerization and activity of ATP synthase. Although monomers of ATP synthase are capable of ATP synthesis, oligomers of ATP synthase have been proven to increase ATP synthesis.^32–34^ Importantly, by using a coupling assay, we showed that the respiratory control ratio was reduced in CM*-Pcsk9^−/−^* mitochondria, confirming a reduction of energy production via ATP synthase-linked respiration.

The assembly and activity of ETC complexes, which are located in the inner mitochondrial membrane, are affected by the membrane composition.^35,36^ The lipid composition of mitochondrial membranes is similar to that of plasma membranes, with PC and PE as the major phospholipids, but with high levels of cardiolipin and low levels of cholesterol.^36^ Most phospholipids are synthesized in the ER and transported into the mitochondria, but the molecular mechanisms and regulation of this transport are still unclear.^36,37^ We did not observe any changes in levels of total phospholipids, PC, PE, or cardiolipin in CM*-Pcsk9^−/−^* mitochondria. However, the fatty acyl chains of PC and PE were altered, potentially reflecting disturbances in the synthesis, degradation, trafficking, or remodelling of these lipids.^36^ We also showed that free cholesterol levels were increased in CM*-Pcsk9^−/−^* mitochondria. As cholesterol-poor organelles, mitochondria are particularly sensitive to changes in cholesterol content, which can have a large impact on the biophysical and functional characteristics of their membranes.^38–40^ Indeed, cholesterol accumulation in liver mitochondria has been shown to disrupt ETC complex assembly, leading to failure of mitochondrial bioenergetics.^41^ The higher cholesterol levels in CM*-Pcsk9^−/−^* mitochondria were not paralleled by changes in cholesterol synthesis pathways in CM*-Pcsk9^−/−^* mouse hearts. However, the increased number of mitochondrial–ER contacts observed in CM*-Pcsk9^−/−^* cardiomyocytes could potentially contribute to the higher cholesterol levels, as these regions are known to be highly enriched in cholesterol.^36^ In addition, the major ER localization of PCSK9 in *Pcsk9*-overexpressing cells supports our hypothesis that ER-retained cardiac PCSK9 could maintain the normal composition of cholesterol and phospholipids in the mitochondrial membrane by modulating their transport through mitochondria-associated ER membrane contacts.

Our electron microscopy studies also revealed striking changes in CM*-Pcsk9^−/−^* mitochondria. Compared with mitochondria from CM*-Pcsk9^+/+^* mice, CM*-Pcsk9^−/−^* mitochondria were rounder, smaller, more often swollen, and had cristae (invaginations of the inner membrane) that were wider and more likely to have an onion-like shape. Mitochondrial matrix swelling often associates with disturbance in the homeostasis of mitochondrial membrane permeability,^42^ but the mitochondrial membrane fluidity was not altered in *Pcsk9*-deficient cardiomyocytes. However, previous studies have shown that the shape of cristae, where most of the ETC complexes are embedded or associated, regulates the ETC and respiratory efficiency.^43^ Cristae biogenesis, shape, and function are regulated by several complexes,^44^ including dimers of ATP synthase (Complex V), which have been shown to maintain cristae curvature.^45^ As noted above, we observed reduced protein levels of ATPase synthase oligomers in CM*-Pcsk9^−/−^* mitochondria. Furthermore, the presence of onion-like cristae structures has been shown to associate with impaired ATP synthase function and dimerization.^46,47^ Further studies are required to determine whether the altered morphology of cristae in CM*-Pcsk9^−/−^* mitochondria directly leads to impaired ETC efficiency or is a consequence of the altered oligomerization of ATP synthase. However, our results indicate that cardiac *Pcsk9* expression is required for mitochondrial energetics, potentially through maintaining homeostasis of mitochondrial membranes and cristae morphology and, subsequently, for preserving normal heart function.

What is the relevance of these studies for human pathophysiology? There are many differences between mouse and human hearts, and numerous studies have shown that studies in mice cannot be directly translated to human pathophysiology. For example, heterozygous deletion of the calcium channel SERCA2a leads to contractile dysfunction in mice, whereas there is no apparent phenotype in humans.^48^ In an attempt to assess the human relevance of results from whole-body *Pcsk9*-deficient mice, Da Dalt *et al*.^10^ evaluated the impact of a low-frequency loss of function (LOF) *PCSK9* variant (R46L) on echocardiographic-based markers of cardiac functionality by analysing data from 12 heterozygous 46L carriers and 516 wild-type humans. The authors reported an association between higher LV mass and the *PCSK9* LOF variant in this small cohort.^10^ However, when we explored this association in a larger sample, leveraging magnetic resonance imaging data from the UK Biobank,^49^ we did not observe any difference in LV mass (beta −0.20, *P* = 0.70) or ejection fraction (beta −0.07, *P* = 0.74) between 578 carriers of 46L and 16 345 wild-type humans. We obtained similar results when we tested these associations for three additional *PCSK9* variants with low allele frequency that have previously been used as genetic instruments for *PCSK9* modulation.^50^ In line, dilated cardiomyopathy has not been reported in *PCSK9*-deficient humans. A confounding factor when assessing the effect of *PCSK9* deficiency in humans is that *PCSK9* variants promote lower LDL-cholesterol. This is an important issue given that Mendelian randomization analyses indicate a potentially causal link between LDL-cholesterol and LV mass.^51^ Our study indicates that PCSK9 mediates intracellular functions that differ from its extracellular functions in mice, but further studies are required to determine the relevance of this finding in humans.

The Cre/lox system is a potent technology to control gene expression in mouse tissues. However, cardiac-specific Cre recombinase expression under the Myh6 promoter can lead to an age-dependent dilated cardiomyopathy-like phenotype.^52–54^ To control this concern, we performed a series of experiments and showed that (i) mice expressing the αMHC-Cre transgene alone had a mean heart weight (per tibia length) that was lower than that of CM-*Pcsk9^−^*^/−^ mice and similar to that of CM-*Pcsk9^+/+^* mice at 28 weeks, (ii) expression of the transgene did not reduce lifespan of the mice, and (iii) expression of the transgene had no effect on mitochondrial function and energetic impairment in cardiomyocytes from 28-week-old mice. Although these results indicate that the transgene *per se* does not promote cardiac hypertrophy or lifespan at the timepoints analysed, we cannot fully exclude cardiotoxic off-target effects of Cre recombinase under the Myh6 promoter. This is a weakness of the study.

In summary, here, we show that PCSK9 in cardiomyocytes, despite its low expression levels, affects cardiac metabolism and function in mice. Future studies are needed to further clarify the underlying mechanisms and the relevance of this finding to the effects of anti-PCSK9 therapies clinically available.

## Supplementary material

Supplementary material is available at *Cardiovascular Research* online.

## Authors’ Contributions

M.La., M.P., M.C.L., and J.B. contributed to study conception and design; M.La., M.Li., M.C., M.R., A.M., M.K., M.R., L.A., M.H., P.O.B., N.A., J.G.S., M.P., T.H., and M.O. contributed to data acquisition or analysis; and M.La., M.A., and S.D. contributed to the interpretation of data. M.La. and R.P. drafted the original and revised manuscripts. All authors approved the final version of the manuscript.

## Supplementary Material

cvad041_Supplementary_Data

## Data Availability

Mice generated in this study are available to any researcher upon request. Original immunoblots have been deposited at Mendeley https://data.mendeley.com/datasets/wnpdm3bnzz/1. Raw RNA-seq data were deposited at NCBI Gene Expression Omnibus and are accessible through GEO series accession number GSE178389. Please direct requests for further information, resources, and reagents to the Lead Contact, Jan Borén (jan.boren@wlab.gu.se).
