# Supplementary material for: Cardiomyocyte-specific PCSK9 deficiency compromises mitochondrial bioenergetics and heart function
Source: Cardiovasc Res. 2023 Mar 6;119(7):1537–52. doi: 10.1093/cvr/cvad041 (PMC10318396; doi:10.1093/cvr/cvad041)

SUPPLEMENTARY MATERIAL

Cardiomyocyte-specific PCSK9 deficiency compromises mitochondrial bioenergetics and heart function

Marion Laudette,[1] Malin Lindbom,[1] Muhammad Arif,[2] Mathieu Cinato,[1] Mario Ruiz,[3] Stephen Doran,[4] Azra Miljanovic,[1] Mikael Rutberg,[1] Linda Andersson,[1] Martina Klevstig,[1] Marcus Henricsson,[1] Per-Olof Bergh,[1] Entela Bollano,[5] Nay Aung[6,7,8], J. Gustav Smith[1,5], Marc Pilon,[3] Tuulia Hyötyläinen,[9] Matej Orešič,[10,11], Rosie Perkins[1], Adil Mardinoglu,[2,4] Malin C. Levin[1] and Jan Borén[1,12]

[1]Department of Molecular and Clinical Medicine/Wallenberg Laboratory, Institute of Medicine, the Sahlgrenska Academy at University of Gothenburg, Gothenburg, Sweden; [2]Science for Life Laboratory, Royal Institute of Technology, Stockholm, Sweden; [3]Department of Chemistry and Molecular Biology, University of Gothenburg, Gothenburg, Sweden; [4]Centre for Host-Microbiome Interactions, Faculty of Dentistry, Oral & Craniofacial Sciences, King's College London, London, United Kingdom; [5]Department of Cardiology, Sahlgrenska University Hospital, Gothenburg, Sweden; [6]William Harvey Research Institute, Barts and The London School of Medicine and Dentistry, Queen Mary University of London, United Kingdom; [7]National Institute for Health Research, Barts Cardiovascular Biomedical Research Centre, Queen Mary University of London, United Kingdom; [8]Barts Heart Centre, St Bartholomew's Hospital, Barts Health National Health Service Trust, West Smithfield, London, United Kingdom; [9]School of Natural Sciences and Technology, Örebro University, Örebro, Sweden; [10]School of Medical Sciences, Örebro University, Örebro, Sweden; [11]Turku Bioscience Centre, University of Turku, Turku, Finland; [12]Sahlgrenska University Hospital, Gothenburg, Sweden

SUPPLEMENTARY TABLES

Table S1: Transcriptomics results of genes located close to the *Pcsk9* locus from RNA-seq data performed on 10- and 28-week-old CM-*Pcsk9*$^{+/+}$ and CM-*Pcsk9*$^{-/-}$ mouse heart. Log2 of Fold Changes (L2FC), P-Value, and adjusted P-Value (P-ADJ) were retrieved from the differential expression analyses using DESeq2.

	DEG 10 weeks			DEG 28 weeks		
**Gene**	**L2FC**	**P-VALUE**	**P-ADJ**	**L2FC**	**P-VALUE**	**P-ADJ**
Usp24	-0.148	0.228	0.985	-0.157	0.2084	0.485
Bsnd	not detected (expression = 0) in our data, in line with the data sources HPA RNA-seq normal tissues, GTEX Analysis Human database and single cell snRNA-seq pilot					
Dhcr24	-0.032	0.904	0.998	0.064	0.832	0.937
Pars2	-0.259	0.285	0.998	-0.278	0.305	0.594
Acot11	-0.343	0.093	0.879	-0.417	0.046	0.193
Ssbp3	-0.107	0.561	0.998	0.091	0.643	0.843
Tmem61	not detected (expression = 0) in our data, in line with the data sources HPA RNA-seq normal tissues, GTEX Analysis Human database and single cell snRNA-seq pilot					
Lexm	not detected (expression = 0) in our data, in line with the data sources HPA RNA-seq normal tissues, GTEX Analysis Human database and single cell snRNA-seq pilot					
Ttc4	-0.0184	0.901	0.998	0.145	0.383	0.667

Table S2: Echocardiographic analysis of cardiac function from 10- and 28-week-old CM-*Pcsk9*$^{-/-}$ mice. Values are mean ± SEM. *p<0.05, **p<0.01, ***p<0.001, ****p<0.0001 vs CM-*Pcsk9*$^{+/+}$ by two-tailed t test.

**10 weeks**	**Baseline (10w)**		**Dobutamine stress (10w)**		**Δ(Dobutamine-Baseline) (10w)**	
	CM-*Pcsk9*$^{+/+}$ (n=6)	CM-*Pcsk9*$^{-/-}$ (n=8)	CM-*Pcsk9*$^{+/+}$ (n=6)	CM-*Pcsk9*$^{-/-}$ (n=8)	CM-*Pcsk9*$^{+/+}$ (n=6)	CM-*Pcsk9*$^{-/-}$ (n=8)
**Simpson**						
LV diastolic volume (μL)	48.34 ± 5.76	**63.26 ± 6.32** ***	34.08 ± 5.89	**46.55 ± 12.61** *	-14.26 ± 4.61	-16.71 ± 7.80
LV systolic volume (μL)	13.40 ± 3.45	**21.83 ± 4.77** **	5.03 ± 1.82	**11.15 ± 5.88** *	-8.36 ± 3.41	-10.69 ± 5.20
Ejection Fraction (%)	72.41 ± 5.71	**65.82 ± 5.05** *	85.49 ± 3.74	**77.20 ± 6.06** *	13.08 ± 6.49	11.38 ± 6.87
Stroke Volume (μL)	34.95 ± 4.50	**41.43 ± 2.82** **	29.05 ± 4.52	35.40 ± 7.41	-5.90 ± 2.81	-6.03 ± 5.73
Cardiac Output (mL/min)	15.82 ± 2.18	18.85 ± 3.01	14.25 ± 2.03	17.54 ± 3.34	-1.57 ± 1.86	-1.31 ± 4.26
Cardiac Index (μL/g)	0.53 ± 0.09	0.61 ± 0.11	0.47 ± 0.08	0.57 ± 0.11	-0.05 ± 0.06	-0.04 ± 0.14
**Long Axis View**						
LV diastolic length	8.08 ± 0.57	8.08 ± 0.36	6.84 ± 0.82	7.40 ± 0.94	-1.24 ± 0.66	-0.68 ± 0.66
LV systolic length	6.82 ± 0.62	6.82 ± 0.62	5.77 ± 0.82	6.19 ± 0.92	-1.05 ± 0.71	-0.63 ± 0.56
**Short Axis B-mode**						
LV Mass (mg)	107.99 ± 8.11	120.55 ± 15.42	91.12 ± 9.39	**112.14 ± 11.26** **	-16.87 ± 8.96	-8.41 ± 15.15
Average Wall Thickness (mm)	0.93 ± 0.05	0.94 ± 0.09	0.92 ± 0.08	0.98 ± 0.09	-0.01 ± 0.09	0.04 ± 0.11
**Short Axis View M-mode**						
Heart Rate (bpm)	454.77 ± 53.61	454.61 ± 61.14	492.30 ± 32.24	497.68 ± 25.35	37.54 ± 29.95	43.07 ± 62.84
LV anterior wall diastolic dimension (mm)	0.85 ± 0.12	0.96 ± 0.14	0.95 ± 0.10	1.00 ± 0.15	0.11 ± 0.16	0.04 ± 0.18
LV posterior wall diastolic dimension (mm)	0.69 ± 0.09	0.70 ± 0.13	0.77 ± 0.13	0.74 ± 0.06	0.08 ± 0.18	0.04 ± 0.13
LV end-diastolic dimension (mm)	3.39 ± 0.26	3.68 ± 0.25	3.21 ± 0.18	**3.47 ± 0.23** *	-0.18 ± 0.21	-0.21 ± 0.24
LV end-systolic dimension (mm)	1.90 ± 0.28	**2.33 ± 0.32** *	1.46 ± 0.26	**1.79 ± 0.29** *	-0.45 ± 0.16	-0.54 ± 0.32
Fractional Shortening (%)	43.97 ± 5.68	**36.81 ± 5.19** *	54.83 ± 5.99	48.64 ± 5.86	10.85 ± 5.82	11.83 ± 5.96
Ejection Fraction (%)	75.94 ± 6.06	**67.2 ± 7.04** *	86.16 ± 4.76	80.56 ± 5.80	10.23 ± 5.14	13.36 ± 6.55
LV diastolic volume (μL)	47.52 ± 8.67	57.69 ± 9.09	41.37 ± 5.50	**50.24 ± 7.69** *	-6.15 ± 6.84	-7.44 ± 8.90
LV systolic volume (μL)	11.59 ± 4.04	**19.30 ± 6.58** *	5.91 ± 2.69	9.99 ± 4.27	-5.69 ± 2.45	-9.32 ± 5.89
Relative Wall Thickness	0.46 ± 0.07	0.46 ± 0.08	0.54 ± 0.09	0.50 ± 0.06	3.31 ± 7.89	0.05 ± 0.08

**28 weeks**	**Baseline (28w)**		**Dobutamine stress (28w)**		**Δ(Dobutamine-Baseline) (28w)**	
	CM-*Pcsk9*$^{+/+}$ (n=10)	CM-*Pcsk9*$^{-/-}$ (n=10)	CM-*Pcsk9*$^{+/+}$ (n=10)	CM-*Pcsk9*$^{-/-}$ (n=10)	CM-*Pcsk9*$^{+/+}$ (n=10)	CM-*Pcsk9*$^{-/-}$ (n=10)
**Simpson**						
LV diastolic volume (μL)	56.44 ± 9.08	67.56 ± 14.42	35.12 ± 3.77	**52.72 ± 13.03** ***	-21.33 ± 10.50	-14.84 ± 7.62
LV systolic volume (μL)	21.81 ± 9.93	28.63 ± 9.98	3.52 ± 2.00	**17.66 ± 6.11** ****	-18.29 ± 10.80	-10.98 ± 6.44
Ejection Fraction (%)	62.73 ± 12.50	58.44 ± 7.02	89.98 ± 5.16	**67.08 ± 3.51** ****	27.25 ± 14.55	**8.65 ± 5.94** **
Stroke Volume (μL)	34.64 ± 4.34	38.93 ± 6.81	31.60 ± 3.86	35.06 ± 7.20	-3.04 ± 4.41	-3.87 ± 5.40
Cardiac Output (mL/min)	12.78 ± 3.62	**17.11 ± 3.24** *	16.16 ± 2.99	17.50 ± 3.73	3.38 ± 4.52	0.39 ± 2.52
Cardiac Index (μL/g)	0.32 ± 0.12	**0.43 ± 0.10** *	0.40 ± 0.09	0.44 ± 0.09	0.08 ± 0.11	0.01 ± 0.06
**Long Axis View**						
LV diastolic length	7.52 ± 0.36	7.85 ± 0.38	7.05 ± 0.40	7.37 ± 0.55	-0.48 ± 0.39	-0.48 ± 0.37
LV systolic length	6.56 ± 0.52	6.86 ± 0.59	5.67 ± 0.34	**6.31 ± 0.58** **	-0.90 ± 0.50	-0.54 ± 0.37
**Short Axis B-mode**						
LV Mass (mg)	148.09 ± 15.06	152.07 ± 27.65	137.19 ± 9.77	142.43 ± 26.43	-10.9 ± 18.21	-9.64 ± 24.79
Average Wall Thickness (mm)	1.14 ± 0.1	1.11 ± 0.11	1.26 ± 0.07	**1.13 ± 0.12** **	0.11 ± 0.11	0.03 ± 0.12
**Short Axis View M-mode**						
Heart Rate (bpm)	366.99 ± 78.25	**440.57 ± 47.26** *	510.19 ± 59.87	498.88 ± 18.40	143.20 ± 93.36	**58.31 ± 40.23** *
LV anterior wall diastolic dimension (mm)	0.96 ± 0.18	0.91 ± 0.20	1.16 ± 0.13	**0.92 ± 0.11** ***	0.20 ± 0.24	0.01 ± 0.21
LV posterior wall diastolic dimension (mm)	0.84 ± 0.10	0.82 ± 0.19	0.97 ± 0.10	**0.83 ± 0.09** **	0.12 ± 0.11	0.015 ± 0.18
LV end-diastolic dimension (mm)	3.83 ± 0.40	**4.21 ± 0.26** *	3.27 ± 0.27	**3.97 ± 0.39** ***	-0.56 ± 0.46	-0.24 ± 0.26
LV end-systolic dimension (mm)	2.45 ± 0.61	**2.94 ± 0.36** *	1.19 ± 0.27	**2.51 ± 0.40** ****	-1.26 ± 0.74	**-0.43 ± 0.23** **
Fractional Shortening (%)	36.79 ± 11.12	30.32 ± 5.15	63.71 ± 5.94	**37.03 ± 6.12** ****	26.92 ± 13.94	**6.71 ± 3.88** ***
Ejection Fraction (%)	65.79 ± 12.57	57.74 ± 7.89	92.03 ± 4.01	**67.08 ± 8.25** ****	26.25 ± 14.50	**9.34 ± 5.43** **
LV diastolic volume (μL)	64.02 ± 15.63	**79.42 ± 11.63** *	43.61 ± 8.93	**69.87 ± 16.20** ***	-20.41 ± 17.23	-9.55 ± 10.50
LV systolic volume (μL)	23.32 ± 11.66	**34.08 ± 10.56** *	3.66 ± 2.50	**23.40 ± 10.11** ****	-19.66 ± 12.78	-10.68 ± 5.85
Relative Wall Thickness	0.48 ± 0.11	0.41 ± 0.07	0.65 ± 0.08	**0.44 ± 0.06** ****	0.17 ± 0.14	**0.03 ± 0.10** *

Table S3: List of differentially expressed genes (DEG), Gene Ontology biological processes (GO) and Kyoto Encyclopedia of Genes and Genome (KEGG) from RNA-seq data performed on 10- and 28-week-old CM-*Pcsk9*$^{+/+}$ and CM-*Pcsk9*$^{-/-}$ mouse heart

Table S4: Oligonucleotides used

OLIGONUCLEOTIDES	SOURCE	REFERENCE
Pcsk9 (Rat) siRNA-1#: GGGUAUAGCCGGAUCCUUA siGENOME SMARTpool siRNA D-092892-01	Dharmacon	M-092892-01-0010
Pcsk9 (Rat) siRNA-2#: UCAUAGGCCUGGAGUUUAU siGENOME SMARTpool siRNA D-092892-02		
Pcsk9 (Rat) siRNA-3#: GGUGGAGGUGUAUCUCUUA siGENOME SMARTpool siRNA D-092892-03		
Pcsk9 (Rat) siRNA-4#: GCUCAACUGUCAAGGGAAG siGENOME SMARTpool siRNA D-092892-04		
Non-Targeting siRNA (Rat) siRNA-1#: UAAGGCUAUGAAGAGAUAC siGENOME control pool	Dharmacon	D-001206-14-20
Non-Targeting siRNA (Rat) siRNA-2#: AUGUAUUGGCCUGUAUUAG siGENOME control pool		
Non-Targeting siRNA (Rat) siRNA-3#: AUGAACGUGAAUUGCUCAA siGENOME control pool		
Non-Targeting siRNA (Rat) siRNA-4#: UGGUUUACAUGUCGACUAA siGENOME control pool		

Table S5: Designed primers used

GENE (Rat)	FORWARD (5´-3´)	REVERSE (5´-3´)
Pcsk9	TCAACTGTCAAGGGAAGGGC	GCTCTTCCGAATAAACTCCAGG
Tnnt2	GCTCTGTCCAACATGATGCATT	CTTCCCACTCTTCCGCTCTG
Ndufv1	TGAAGGCAAGCAGGGAAAGC	AGCCACCGTCTCCACATTG
Acsl3	TTTGTGAGACCAGGGCTGAG	CCCAGAGTCGCATACAGTGT
Acaa2	TGTCATCGTGGGCAATGTCA	CGTAAACCCACATGCCTTGC
Acadl	AAAGGTCTGGGAGTGATTGGA	ACGAGATCACTTAACCAGCCA
Pdha1	TGGAGCTACAGACTTACCGC	TCCTGGATTTCTTCTCGAGTGC
Atp2a2	TGGAACTTGTGATCGAGCAGT	ACCTTCTTCGAACCAAGCCA
Pln	TCACACAACTAAACAGTCTGCA	GCCAGGAAGACAAAAGTAGGA
Hadh	GCCGCCAAGAAGATCCTGAT	AGGATGTCTTCTGTTTGGTCCA
Pdk4	CGTCGCCAGAATTAAAGCTCA	CACGATGTGGATTGGTTGGC
Rplpo	GTGATGCCCAGGGAAGACAG	CTGCTCCCACAATGAAGCAT

Table S6: Taqman primers used

Taqman	SOURCE	IDENTIFIER
TaqMan assay ANP (*Nppa*)	Thermo Scientific™	Mm1255747_g1
TaqMan assay *Hprt*	Thermo Scientific™	Mm01545399_m1
TaqMan assay BNP (*Nppb*)	Thermo Scientific™	Mm00435304_g1
TaqMan assay *Myh6*	Thermo Scientific™	Mm00440359_m1
TaqMan assay *Myh7*	Thermo Scientific™	Mm01319006g1
TaqMan assay *Actc1*	Thermo Scientific™	Mm01333821m1
TaqMan assay *Csrp3*	Thermo Scientific™	Mm00443379_m1
TaqMan assay *Ctnna3*	Thermo Scientific™	Mm01173713_m1
TaqMan assay *Dmd*	Thermo Scientific™	Mm01216951_m1
TaqMan assay *Scn1b*	Thermo Scientific™	Mm00441208_m1
TaqMan assay *Trdn*	Thermo Scientific™	Mm00661493_m1
TaqMan assay *Col3a1*	Thermo Scientific™	Mm01254476_m1
TaqMan assay *Tgfb1*	Thermo Scientific™	Mm01178820_m1
TaqMan assay *Ucp3*	Thermo Scientific™	Mm00494077_m1
TaqMan assay *Ucp2*	Thermo Scientific™	Mm00627599_m1
TaqMan assay *Cd36*	Thermo Scientific™	Mm01135198_m1
TaqMan assay *Cpt1a*	Thermo Scientific™	Mm00550438_m1
TaqMan assay *Acadl*	Thermo Scientific™	Mm01323360_g1
TaqMan assay *Hadh*	Thermo Scientific™	Mm00492535_m1
TaqMan assay *Slc2a1*	Thermo Scientific™	Mm00441480_m1
TaqMan assay *Hk2*	Thermo Scientific™	Mm00443385_m1
TaqMan assay *Pfkm*	Thermo Scientific™	Mm01309576_m1
TaqMan assay *Pfkfb3*	Thermo Scientific™	Mm00504650_m1
TaqMan assay *Pgk1*	Thermo Scientific™	Mm00435617_m1
TaqMan assay *Pkm2*	Thermo Scientific™	Mm00834102_gH
TaqMan assay *Ldha*	Thermo Scientific™	Mm01612132_g1
TaqMan assay *Ldhb*	Thermo Scientific™	Mm01267402_m1
TaqMan assay *Pdk2*	Thermo Scientific™	Mm00446681_m1
TaqMan assay *Pdk4*	Thermo Scientific™	Mm01166879_m1
TaqMan assay *Pdha1*	Thermo Scientific™	Mm00468675_m1
TaqMan assay *G6pdx*	Thermo Scientific™	Mm00656735_g1
TaqMan assay *Gfm2*	Thermo Scientific™	Mm00623824_m1
TaqMan assay *Ndufv1*	Thermo Scientific™	Mm00504941_m1
TaqMan assay *Nubpl*	Thermo Scientific™	Mm01284240_g1
TaqMan assay *Tmlhe*	Thermo Scientific™	Mm00454748_m1
TaqMan assay *Myh6*	Thermo Scientific™	Rn00691721_g1
TaqMan assay *Myh7*	Thermo Scientific™	Rn01488777_g1
TaqMan assay *Rplpo*	Thermo Scientific™	Rn03302271_gH
TaqMan assay *Nppa*	Thermo Scientific™	Rn00664637_g1
TaqMan assay *Nppb*	Thermo Scientific™	Rn00580641_g1
TaqMan assay *PCSK9*	Thermo Scientific™	Hs00545399_m1
TaqMan assay *HPRT1*	Thermo Scientific™	Hs02800695_m1

Table S7: Antibodies used for western blotting

ANTIBODY	SOURCE	IDENTIFIER
PCSK9	R&D Systems, Inc.	AF3985; RRID:AB_2044717
GAPDH	Abcam	ab8245 RRID:AB_2107448
NDUFB8	Abcam	ab110242; RRID:AB_10859122
SDHA	Abcam	ab14715; RRID:AB_301433
UQCRC2	Abcam	ab14745; RRID:AB_2213640
COXIV	Abcam	ab202554; RRID:AB_2861351
ATP5A	Abcam	ab14748; RRID:AB_301447
UCP3	Abcam	ab3477; RRID:AB_2304253
UCP2	Cell Signaling Technology	89326S; RRID:AB_2721818
Phospho-ACC	Cell Signaling Technology	3661S; RRID:AB_330337
ACC	Cell Signaling Technology	3662S; RRID:AB_2219400
Phospho-AMPK	Cell Signaling Technology	2535; RRID:AB_331250
AMPK	Cell Signaling Technology	2603; RRID:AB_490795
ACADL	Abcam	ab82853; RRID:AB_1859818
GLUT1	Abcam	ab115730; RRID:AB_10903230
GLUT4	Abcam	ab35826; RRID:AB_732611
Hexokinase II	Cell Signaling Technology	2867; RRID:AB_2232946
LDHA	Cell Signaling Technology	2012S; RRID:AB_2137173
PFKFB3	Cell Signaling Technology	13123S; RRID:AB_2617178
Phospho-PDH	Cell Signaling Technology	31866S; RRID: AB_2799014
PDH	Cell Signaling Technology	3205S; RRID: AB_2162926
PGC1α	Abcam	ab54481; RRID:AB_2137173
MFN2	Abcam	ab124773; RRID:AB_10999860
DRP1	Cell Signaling Technology	8570; RRID:AB_10950498
ACSL3	Abcam	ab80675; RRID:AB_1658567
CTnT	Abcam	ab8295; RRID:AB_306445
SERCA2	Cell Signaling Technology	9580; RRID:AB_10827913
PLB	Cell Signaling Technology	14562; RRID:AB_2798511
β-actin	Abcam	ab8226; RRID:AB_306371
GFP	Abcam	ab290; RRID:AB_303395
PCSK9 Human	Abcam	ab181142
PCSK9 Mouse	Abcam	ab125251; RRID:AB_11130454
Vinculin	Abcam	ab18058 ; RRID:AB_444215

SUPPLEMENTARY FIGURES

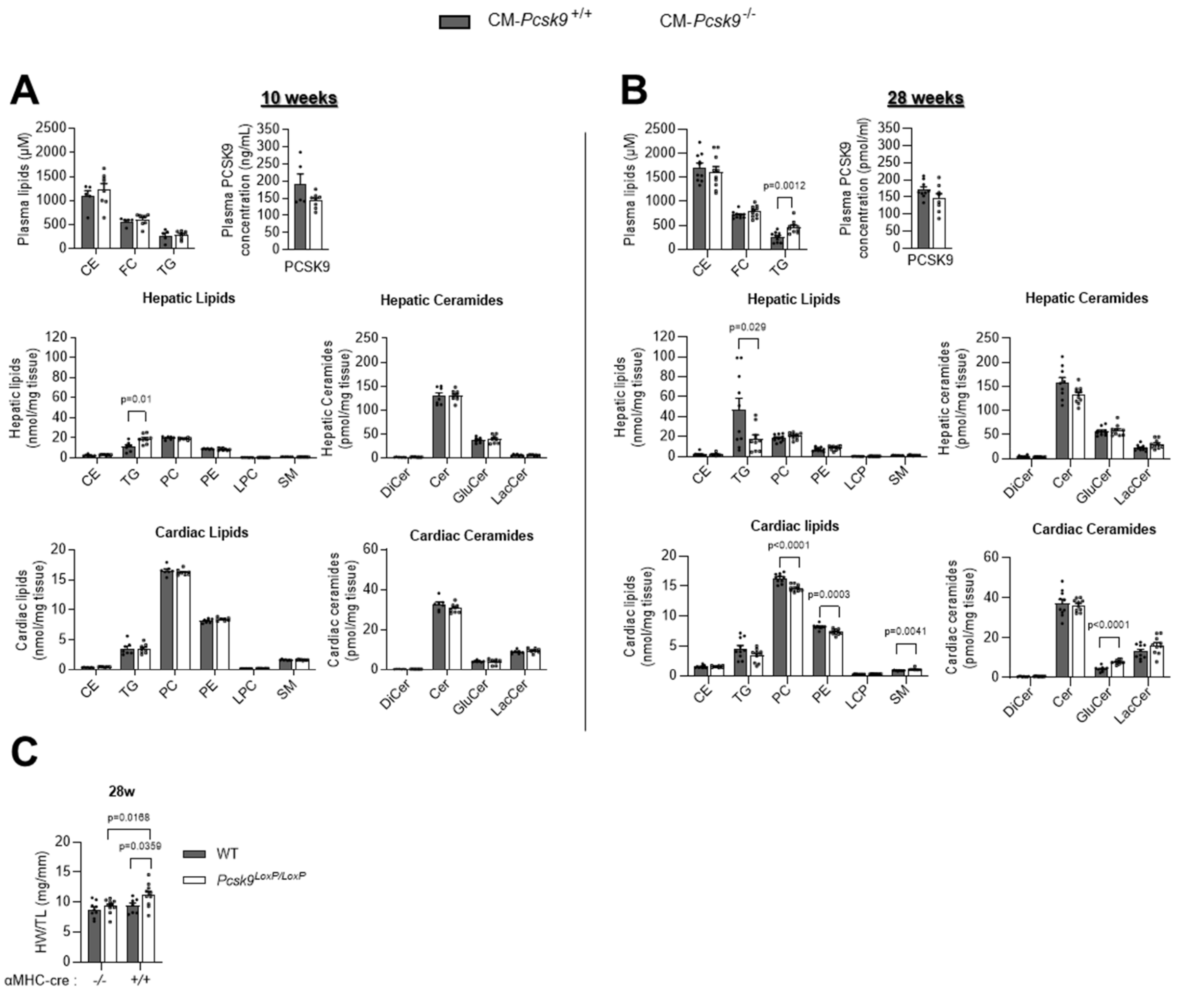

Figure S1. Characterization of CM-*Pcsk9*$^{-/-}$ mice (related to Figure 2) (A and B) Levels of plasma lipids, plasma PCSK9 and neutral lipids, phospholipids, and ceramide species in hepatic and cardiac tissues from (**A**) 10-week-old (n=7–8) and (**B**) 28-week-old (n=10) CM-*Pcsk9*$^{+/+}$ and CM-*Pcsk9*$^{-/-}$ mice. **(C)** Heart weight/tibia length (HW/TL) of 28-week-old WT and *Pcsk9*$^{\mathrm{LoxP/LoxP}}$ without or with expression of the αMHC-Cre transgene (n=7–10). Values are mean ± SEM. P values are shown in the figure vs CM-*Pcsk9*$^{+/+}$ by two-tailed t test (A-B) or two-way ANOVA (C). CE, cholesteryl ester; FC, free cholesterol; TG, triglyceride; PC, phosphatidylcholine; PE, phosphoethanolamine; LPC, lysophosphatidylcholine; SM, sphingomyelin; DiCer, dihydroceramide; Cer, ceramide; GlcCer, glucosylceramide; LacCer, lactosylceramide.

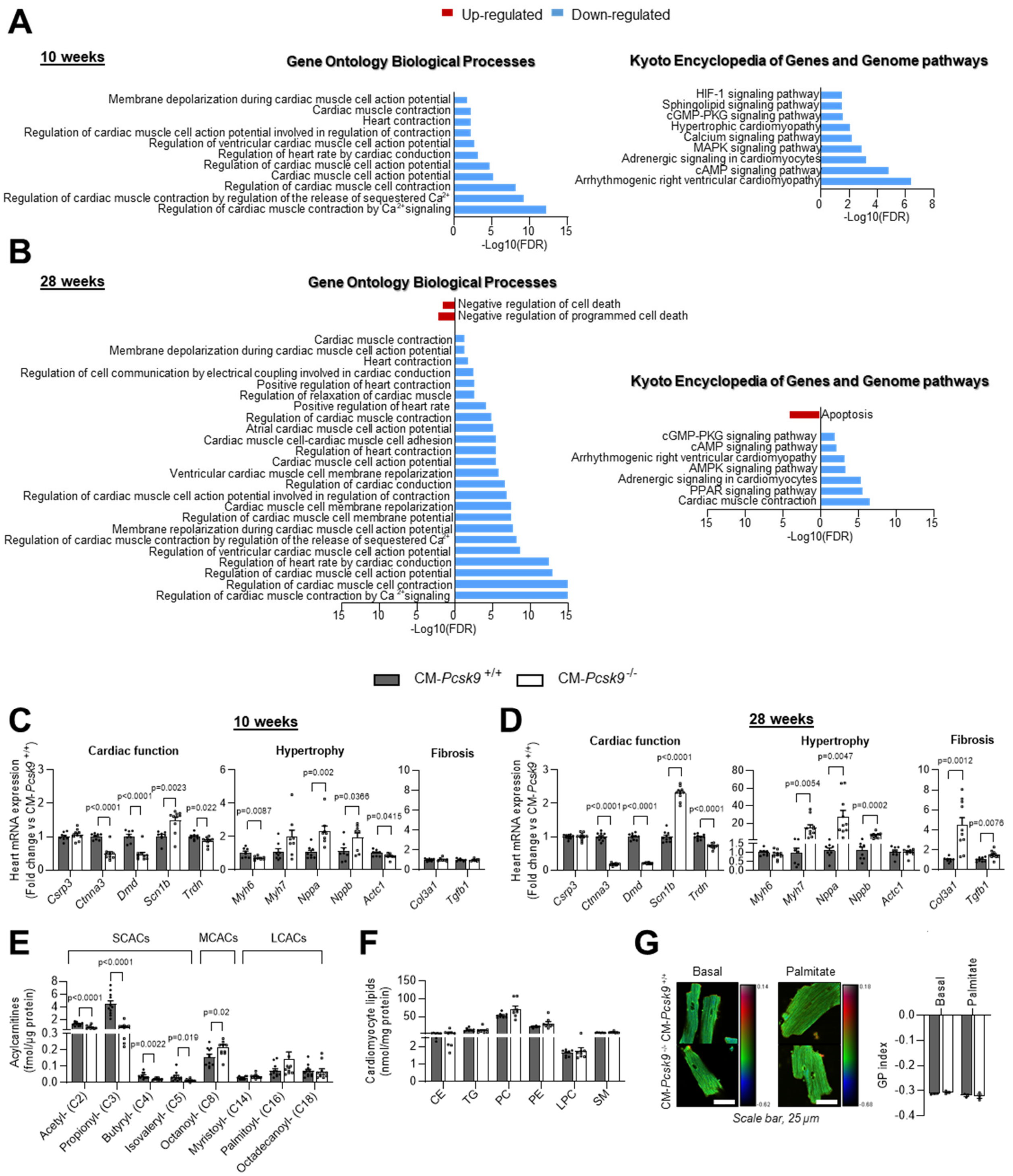

Figure S2. Cardiac mitochondrial metabolism is impaired in CM-*Pcsk9*$^{-/-}$ mice (related to Figure 3) (**A and B**) Significantly up- and down-regulated Gene Ontology biological processes and Kyoto Encyclopedia of Genes and Genome pathways related to cardiomyopathy and cardiac function in RNA-seq data from cardiac tissues from (**A**) 10-week-old and (**B**) 28-week-old CM-*Pcsk9*$^{+/+}$ and CM-*Pcsk9*$^{-/-}$ mice (n=5). Statistics retrieved from gene set statistics from PIANO analysis. FDR, false-discovery rate. (**C and D**) Cardiac mRNA expression of genes related to cardiac function, hypertrophy, and fibrosis in (**C**) 10-week-old (n=8) and (**D**) 28-week-old CM-*Pcsk9*$^{+/+}$ and CM-*Pcsk9*$^{-/-}$ mice (n=8–10). (**E**) Acylcarnitine species levels in cardiac mitochondria from 28-week-old CM-*Pcsk9*$^{+/+}$ and CM-*Pcsk9*$^{-/-}$ mice (n=11). SCAC, short-chain acylcarnitine; MCAC, medium-chain acylcarnitine; LCAC, long-chain acylcarnitine. (**F**) Lipid levels in isolated cardiomyocytes

from 28-week-old CM-*Pcsk9*$^{+/+}$ and CM-*Pcsk9*$^{-/-}$ mice (n=8). CE, cholesteryl ester; TG, triglyceride; PC, phosphatidylcholine; PE, phosphoethanolamine; LPC, lysophosphatidylcholine; SM, sphingomyelin. (**G**) Pseudocolor images showing the Laurdan dye generalized polarization (GP) index at each pixel position and average GP index from several images (10–15) in isolated cardiomyocytes (in the absence or presence of 200 μM palmitate) from 28-week-old CM-*Pcsk9*$^{+/+}$ and CM-*Pcsk9*$^{-/-}$ mice (n=3). Red: rigid; blue: fluid. Scale, 25 μm. Values are mean ± SEM. P values are shown vs CM-*Pcsk9*$^{+/+}$ by two-tailed t test.

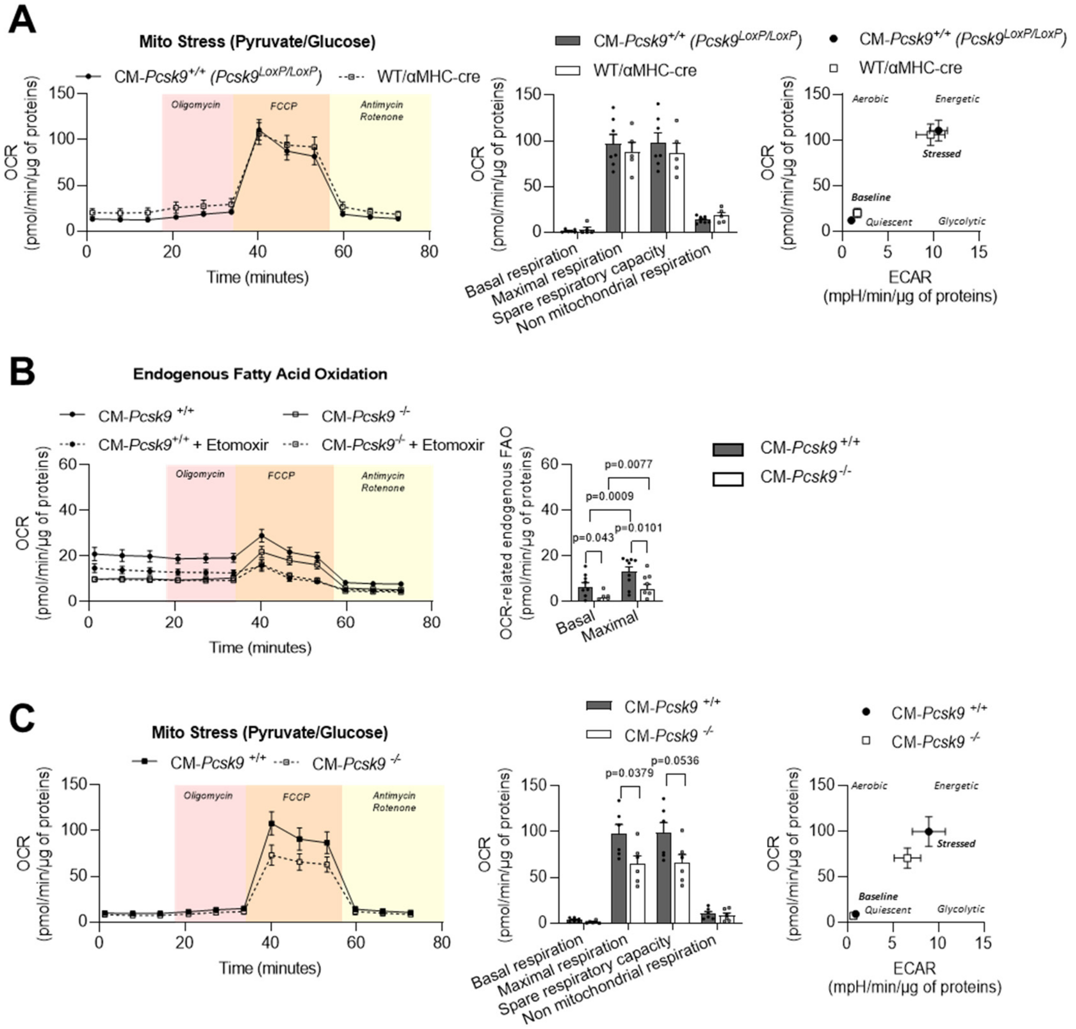

Figure S3. Impairment of mitochondrial but not glycolytic function in CM-*Pcsk9*$^{-/-}$ cardiomyocytes (related to Figure 4) (A) OCR and ECAR were determined using the Seahorse flux analyser in primary cardiomyocytes isolated from 28-week-old wild-type mice expressing αMHC-Cre (WT/αMHC-Cre) and CM-*Pcsk9*$^{+/+}$ mice and normalized to total cellular protein content for each well (12 wells from n=6). OCR (left), quantification of respiratory parameters (middle), and energetic maps obtained by plotting ECAR and OCR values at baseline and under FCCP-induced stress (right). OCR was measured under basal conditions and after addition of oligomycin (1 µM) to inhibit ATP synthase, FCCP (1 µM) to uncouple oxidative phosphorylation, and antimycin A (2 µM) and rotenone (2 µM) to gauge non-mitochondrial respiration. **(B)** OCR was determined using the Seahorse flux analyser in cardiomyocytes from 28-week-old CM-*Pcsk9*$^{+/+}$ and CM-*Pcsk9*$^{-/-}$ mice and normalized to total cellular protein content for each well (12 wells from n=9). Left: Measurement of OCR-related fatty acid oxidation (FAO) in isolated cardiomyocytes pretreated with etomoxir (CPT1B inhibitor, 100 µM, 15 min). Right: Quantification of endogenous fatty acid oxidation at baseline and under FCCP-induced stress (maximal). **(C)** OCR and ECAR were determined using the Seahorse flux analyser in primary cardiomyocytes isolated from 10-week-old CM-*Pcsk9*$^{+/+}$ and CM-*Pcsk9*$^{-/-}$ mice and normalized to total cellular protein content for each well (12 wells from n=6). OCR (left), quantification of respiratory parameters (middle), and energetic maps obtained by plotting ECAR and OCR values at baseline and under FCCP-induced stress (right) using pyruvate/glucose as the substrate. Values are mean ± SEM. P values are shown in the figure vs CM-*Pcsk9*$^{+/+}$ by two-way ANOVA (B) or by two-tailed t test (A-C).

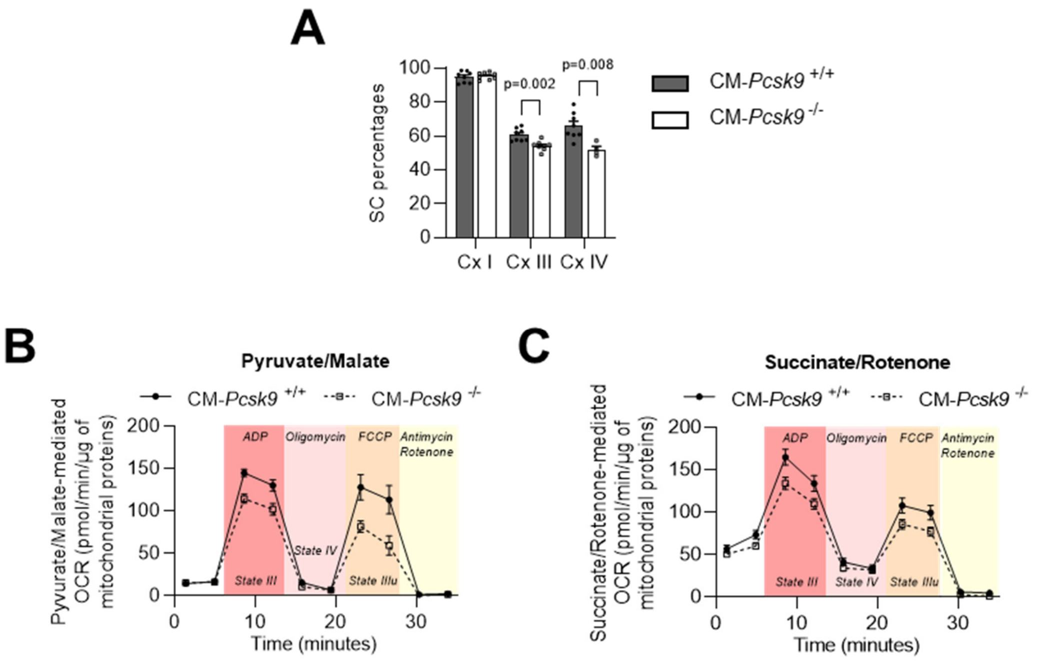

Figure S4. Mitochondrial OXPHOS complexes are altered in CM-***Pcsk9***$^{-/-}$ **mice (related to Figure 5)** (**A**) Percentages of supercomplexes (SCs) containing individual ETC complexes (complex (Cx) I, III and IV) in cardiac mitochondria from 28-week-old CM-*Pcsk9*$^{+/+}$ and CM-*Pcsk9*$^{-/-}$ mice (n=9). (B-C) Measurement of OCR in a coupling assay in cardiac mitochondria from 28-week-old CM-*Pcsk9*$^{+/+}$ and CM-*Pcsk9*$^{-/-}$ mice (n=9). (**B**) Pyruvate/malate (10 mM/5 mM) and (**C**) succinate/rotenone (5 mM/2 µM) were used to drive complex I- and complex II-dependent respiration, respectively. Respiration was initiated by adding ADP (5 mM, State III) and stopped by adding oligomycin (2.5 µM, State IV). FCCP (4 µM) dissipated mitochondrial membrane potential and initiated respiration (State IIIu). Values are mean ± SEM. P values are shown vs CM-*Pcsk9*$^{+/+}$ by t test.

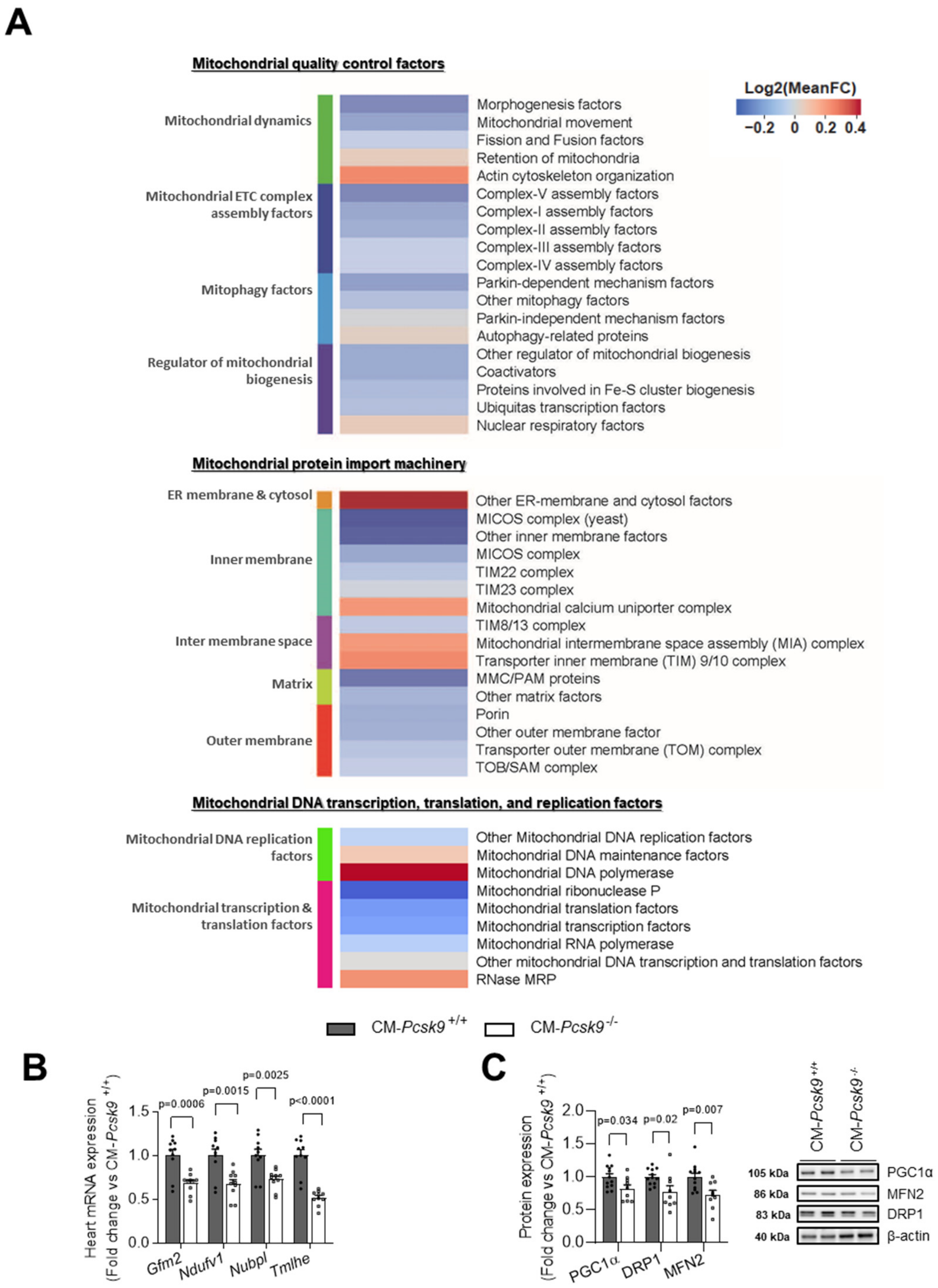

Figure S5. Lipid composition, proximity with ER and morphology of mitochondria are altered in CM-***Pcsk9***$^{-/-}$ **mice (related to Figure 6)** (**A**) List of regulated Gene Ontology biological processes involved in mitochondrial processes in cardiac tissues from 28-week-old CM-*Pcsk9*$^{-/-}$ vs CM-*Pcsk9*$^{+/+}$ mice (n=5). Statistics retrieved from gene set statistics from PIANO analysis. FC, fold change. (**B**) Cardiac mRNA expression of *Gfm2*, *Ndufv1, Nubpl*, and *Tmlhe* in CM-*Pcsk9*$^{-/-}$ and CM-*Pcsk9*$^{+/+}$ mice (n=10). (**C**) Representative immunoblot and quantification of protein levels of PGC1α, MFN2, and DRP1 (markers of mitochondrial biogenesis, fission, and fusion respectively) in isolated cardiomyocytes from CM-*Pcsk9*$^{-/-}$ and CM-*Pcsk9*$^{+/+}$ mice (n=12). β-actin was the loading control. Values are mean ± SEM. P values are shown vs CM-*Pcsk9*$^{+/+}$ by t test.

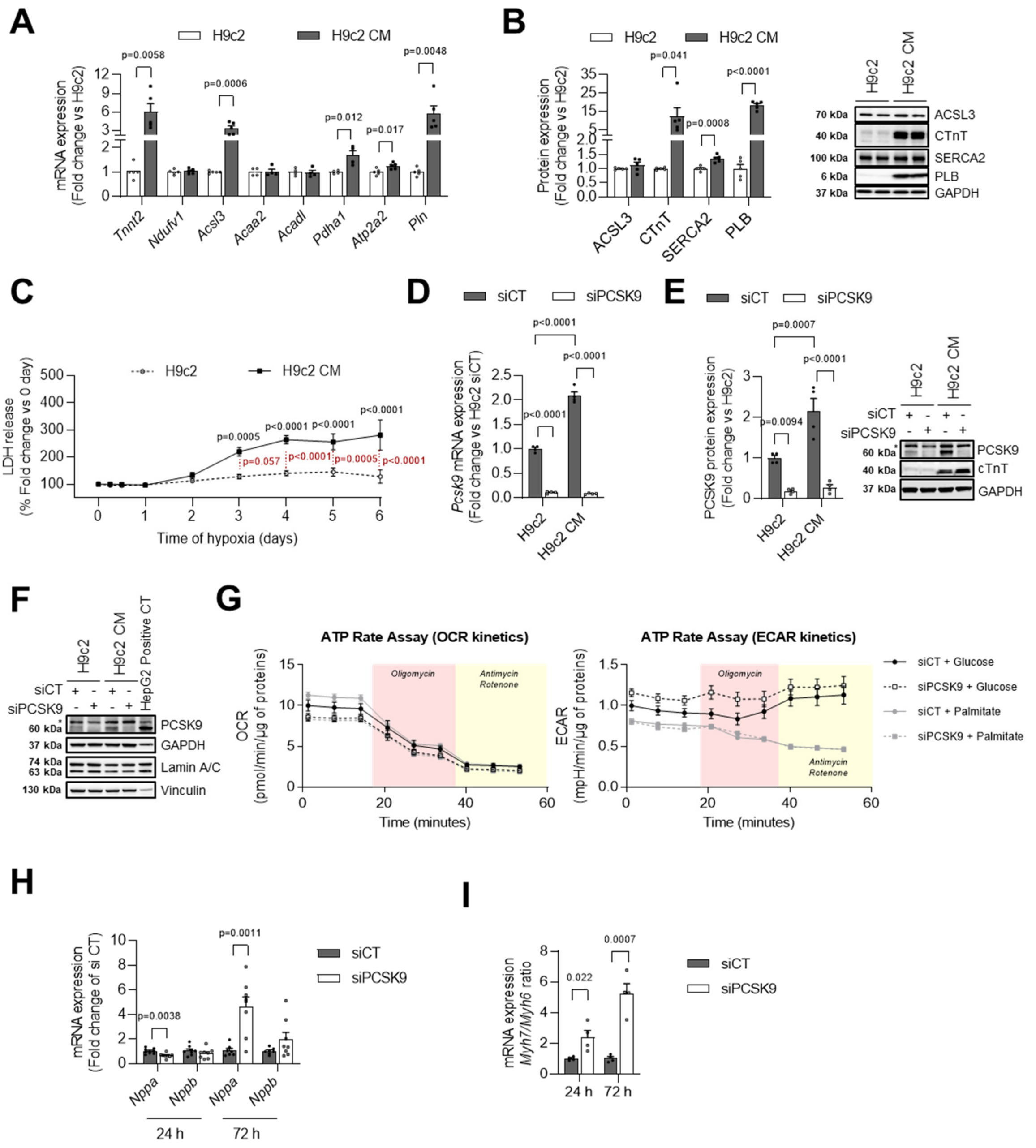

Figure S6. Acute *Pcsk9* silencing causes a shift to glycolytic metabolism in cardiomyocytes and compromises ability to meet increased energy demand (related to Figure 7) (**A**) mRNA expression of *Tnnt2*, *Ndufv1*, *Acsl*, *Acaa2*, *Acadl*, *Pdha1*, *Atp2a2,* and *Pln* in H9c2 cardiomyoblasts (H9c2) and in H9c2 differentiated into adult cardiomyocyte-like cells by treatment with retinoic acid for 5 days (H9c2 cardiomyocytes, CM) (n=5). (**B**) Representative immunoblots and quantification of ACSL3, CtnT, SERCA2 and PLB in H9c2 cardiomyoblasts (H9c2) and H9c2 cardiomyocytes. GAPDH was the loading control (n=5). (**C**) Kinetics of lactate dehydrogenase (LDH) release in H9c2 vs H9c2 cardiomyocytes exposed to prolonged hypoxia (n=4). **(D and E)** *Pcsk9* mRNA **(D)** and protein **(E)** levels in H9c2 cardiomyoblasts (H9c2) and H9c2 cardiomyocytes (H9c2 CM) treated with siRNA against *Pcsk9* (siPCSK9) or scrambled control (siCT) for 24 h (n=4). GAPDH was the loading control and CTnT was a control of cardiac differentiation. **(F)** Representative immunoblots of PCSK9 expression in H9c2 cardiomyoblasts (H9c2) and H9c2 cardiomyocytes (H9c2 CM) compared to cultivated human liver cells HepG2, a positive control of PCSK9 expression (n=4). GAPDH, lamin A/C and vinculin were used as loading control. **(G)** Whole-cell OCR and ECAR measured with Seahorse using glucose or palmitate as

substrate in H9c2 cardiomyocytes (treated with siPCSK9 or scrambled control) after addition of oligomycin (1 μM), antimycin A (1 μM) and rotenone (1 μM). OCR and ECAR were normalized to total cellular protein content for each well (6 wells from 4 independent experiments). **(H and I)** mRNA expression of genes related to cardiac hypertrophy (*Nppa, Nppb*) **(H)** and the ratio *Myh7/Myh6* mRNA expression **(I)** in H9c2 cardiomyocytes treated with siRNA against *Pcsk9* (siPCSK9) or scrambled control (siCT) (n=4-8) for 24 h or 72 h. An increase in this ratio indicates a hypertrophic phenotype. Values are mean ± SEM. P values are shown vs siCT by t test. Values are mean ± SEM. P values are shown vs indicated value by t test (A-C) or by two-way ANOVA (D-E). * The asterisk in E and F indicates nonspecific binding.

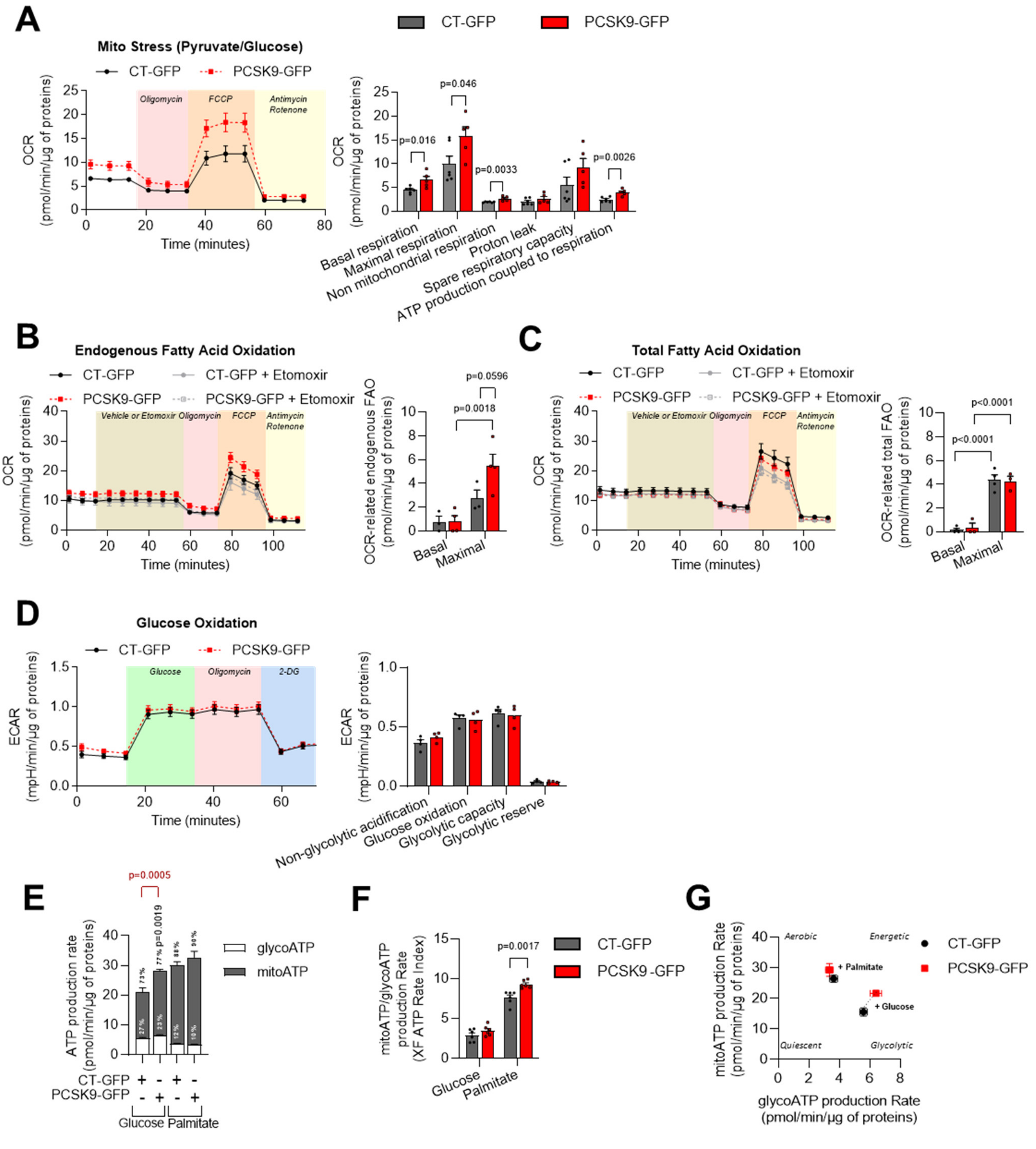

Figure S7. *Pcsk9* overexpression promotes mitochondrial oxidative function. H9c2 cardiomyocytes treated with PCSK9-GFP cDNA (1 µg) or empty control CT-GFP for 24 h were used for all experiments. OCRs and ECARs were determined using the Seahorse flux analyser and ATP production was determined using the Seahorse XF real-time ATP rate assay in H9c2 cardiomyocytes treated with PCSK9-GFP cDNA or empty CT-GFP; values were normalized to total cellular protein content for each well (12 wells from 4–6 independent experiments). **(A)** OCRs and quantification of respiratory and ATP production parameters in H9c2 cardiomyocytes using pyruvate/glucose as substrate, oligomycin (1 µM) to inhibit ATP synthase, FCCP (5 µM) to uncouple oxidative phosphorylation, and antimycin A (1 µM) and rotenone (1 µM) to gauge non-mitochondrial respiration. **(B-C)** Left: OCR-related fatty acid oxidation (FAO) in H9c2 cardiomyocytes pretreated

with etomoxir (40 µM, 15 min) before addition of palmitate (150 µM) at time 0. Right: Quantification of **(B)** endogenous and **(C)** total (endogenous + exogenous) fatty acid oxidation at baseline and under FCCP-induced stress. **(D)** ECARs and quantification of glycolytic parameters in glucose-deprived H9c2 cardiomyocytes after addition of glucose (10 mM) to fuel glycolysis and OXPHOS, oligomycin (2 µM) to inhibit ATP synthase, and 2-deoxyglucose (2-DG) (50 mM) to inhibit glucose catabolism. **(E)** Metabolic flux analysis showing quantification of mitochondrial (mitoATP) and glycolytic (glycoATP) ATP production in H9c2 cardiomyocytes. **(F)** ATP rate index indicating the changes in metabolic phenotype, calculated from data in Panel E. An increase in this index indicates a more oxidative/less glycolytic phenotype. **(G)** Energetic map of mitoATP vs glycoATP of H9c2 cardiomyocytes using carbohydrates (glucose) or fatty acids (palmitate) as substrate (from Panel E). Values are mean ± SEM. P values are shown vs CT-GFP by t test (A, D-F) and two-way ANOVA (B and C).

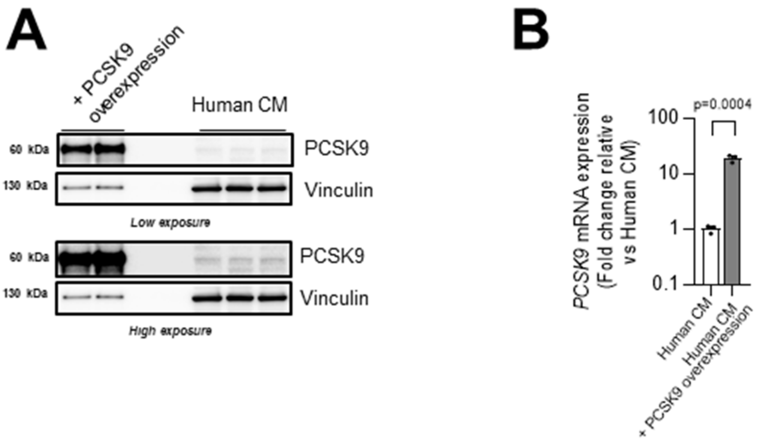

Figure S8. PCSK9 expression in human cardiomyocytes (CM). (A) Representative immunoblots (using human PCSK9 antibody ab181142) of PCSK9 protein expression in lysates from primary human cardiomyocytes (30 µg) isolated from normal ventricle tissue of the adult heart (PromoCell®, C-12810, Sigma-Aldrich). Cell lysate (5 µg) of primary human cardiomyocytes overexpressing human PCSK9 using adeno-associated virus expression (gain of function mutant (D374Y) of human PCSK9, AAV9-HRC-hAAT-PCSK9DY, Vigene Biosciences) was used as a positive control for PCSK9 detection. Vinculin was used as loading control. **(B)** Quantification of A (n=3). Values are mean ± SEM.

SUPPLEMENTARY METHODS

Animals and animal care—All procedures in mice were approved by the local Animal Ethics Committee in Gothenburg (breeding ethics approval 1124-2017) and conform to the guidelines in Directive 2010/63/EU of the European Parliament on the protection of animals used for scientific purposes. Mice were killed by cervical dislocation after an overdose of isoflurane (Forene, AbbVie) (dose 5%).

Mice were kept under temperature-controlled conditions with free access to water and standard rodent chow (12% of calories from protein, 12% from fat, and 66% from carbohydrates) and housed in a pathogen-free barrier facility with a 12 h light, 12 h dark cycle.

Pcsk9$^{LoxP/LoxP}$ mice, in which exons 2 and 3 were flanked with loxP sites, were generated and characterized as described[1] and crossbred with α-myosin heavy chain (α-MHC)-Cre mice (B6.FVB-Tg(Myh6-cre)2182Mds/J, Jackson Laboratory) to generate cardiomyocyte-specific *Pcsk9* knockdown mice (CM-*Pcsk9*$^{-/-}$; *Pcsk9*$^{LoxP/LoxP}$/α-MHC-Cre) and nontransgenic littermate controls (CM-*Pcsk9*$^{+/+}$; *Pcsk9*$^{LoxP/LoxP}$). After a 4-h fast, hearts and other tissues were removed, briefly rinsed in PBS, snap-frozen in liquid N_2, and stored at –80°C. Plasma was also taken after a 4-h fast.

Echocardiography in mice—At baseline and after an intraperitoneal injection of low-dose dobutamine (2 µg/g body weight), 10- or 28-week-old male CM-*Pcsk9*$^{-/-}$ and CM-*Pcsk9*$^{+/+}$ mice were anesthetized with continuous artificially ventilated isoflurane (Forene, AbbVie) (dose 1.2 %) through nose inhalation for the complete period of the intervention; chest hair was removed with gel, the mouse was placed on a heating pad, and extremities were connected to an ECG electrode. Echocardiography was done with a VisualSonics VEVO 2100 system and an ultra-high frequency linear array transducer (MS550D, VisualSonics). An optimal parasternal long-axis cine loop was acquired at >1000 frames/s with the ECG-gated kilohertz visualization technique. Parasternal short axis cine-loops were acquired at 1, 3, and 5 mm below the mitral annulus. End-diastolic and end-systolic LV volumes and EF were calculated by biplane Simpson’s formula using the 3 parasternal short-axis views and the parasternal long-axis view. M-mode measurements at the 3-mm level were done with the leading-edge method. End-diastole was defined at the onset of the QRS complex, and end-systole as the time of peak inward motion of the interventricular septum. At least three beats were averaged for each measurement. The examination was done by an experienced echocardiographer and completed within 30 min after induction of anaesthesia. Data were analysed offline in blinded fashion with VevoLab software (VisualSonics). Mice were excluded from the study if their heart rate did not increase by >100 beats/min after injection of dobutamine.

Isolation and culture of adult primary cardiomyocytes and cardiac fibroblasts—Mice were anesthetized with N-isoflurane, and the heart was rapidly excised. The aorta was cannulated and the heart was perfused with a Langendorff system (PanLab), first with perfusion buffer (120.4 mM NaCl, 14.7 mM KCl, 0.6 mM KH_2PO_4, 0.6 mM Na_2HPO_4, 1.2 mM $MgSO_4$, 10 mM Na-Hepes, 5.5 mM glucose, 4.6 mM $NaHCO_3$, 30 mM taurine, 10 mM BDM, pH 7) for 4 min and then with perfusion buffer containing collagenase type 2 (Worthington) for 3 min and with digestion buffer and 100 mM $CaCl_2$ for 8 min. After collagenase inhibition with fetal calf serum, the heart was torn apart and cardiomyocytes were isolated by centrifugation at 20*g* for 3 min; the supernatant containing non-cardiomyocytes was used for fibroblast isolation. Ca^{2+} was added stepwise to the cardiomyocytes (final concentration, 900 µM), and the cells were seeded in plating medium consisting of minimal essential medial with Hanks balanced salt solution (Lonza), 10% calf serum, 10 mM BDM (Sigma Aldrich), 100 U/ml penicillin (Hyclone), 2 mM L-glutamine (Hyclone), which was equilibrated at 37°C in 18% O_2 and 2% CO_2 to reach an optimal pH, on plates coated with laminin (10 µg/well) (Thermofisher Scientific).

Cardiac fibroblasts were isolated from the non-cardiomyocyte-containing supernatant by centrifugation for 5 min at 2000 rpm and resuspended in fibroblast medium (minimal essential medium with Hanks balanced salt solution, 10% calf serum, 100 U/ml penicillin, 2 mM L-glutamine). After a second centrifugation, cells were seeded and incubated at 37°C in 18% O_2 and 5% CO_2 as described.[2]

We previously assessed the purity of the cardiomyocytes isolated by this protocol and showed that the cardiomyocyte fraction contains 66% cardiomyocytes (cTnT-positive cells), 28% endothelial cells (CD31-positive cells) and 6% other cells (cTnT- and CD31-negative cells).[3]

Lactate dehydrogenase (LDH) release—LDH release in the cell culture medium was measured with the LDH-Cytotoxicity Assay Kit II (Abcam) according to the manufacturer's instructions.

Laurdan dye measurement of membrane fluidity—Freshly isolated cardiomyocytes were seeded in glass-bottom μ-dishes (Ibidi), washed with long-term medium without BSA, stained with 10 μM Laurdan dye (6-dodecanoyl-2-dimethylaminonaphthalene) (Thermo Fisher Scientific) for 45 min and imaged. A second set of cardiomyocytes was treated with 200 μM palmitic acid (previously conjugated to fatty acid–free BSA, 0.5%) in long-term medium supplemented with 20 mM HEPES for 6 h before staining and imaging. Freshly isolated mitochondria were washed with BSA-free MAS buffer, stained with 10 μM Laurdan in BSA-free MAS buffer supplemented with 5 mM malate and 10 mM pyruvate for 45 min. Images were acquired with a 40× water-immersion objective on a Zeiss LSM880 confocal microscope equipped with a live cell chamber (37°, 5% CO_2) and ZEN software. Cells were excited with a 405 nm laser, and emission was recorded between 410 and 461 nm (ordered phase) and between 470 and 530 nm (disordered phase). Pictures were acquired with 16-bit image depth, 1024 × 1024 resolution, and a pixel dwell time of ~1.02 μs, as described.[4] The generalized polarization (GP) index was calculated with ImageJ version 1.47 as described.[5]

Culture and differentiation of H9c2 cells—H9c2 cells (CRL-1446, America Tissue Type Collection) were cultured in high-glucose Dulbecco's modified Eagle's medium (DMEM) supplemented with 10% fetal bovine serum, 100 U/mL penicillin and 100 μg/mL streptomycin at 37°C in a humidified atmosphere of 95% air/5% CO_2. Cells were fed every 2–3 days, and subcultured at 70–80% confluency to prevent the loss of differentiation potential. H9c2 cells in medium containing 1% fetal calf serum were differentiated by daily addition of 1 μM retinoic acid for 5 days.[6] Retinoic acid was prepared in DMSO and stored at –20°C in the dark.

siRNA experiments— SMARTpool siRNA reagent, which is a mixture of four different siRNAs targeting rat Pcsk9 gene (siPCSK9) or control (siCT) were resuspended at 20 µmol/l (Horizon Discovery) in distilled water. The day of experiment, H9c2 cardiomyocytes were transfected using 30 pmol/L of siRNA per well and Lipofectamine RNAiMax transfection reagent (Invitrogen) according to the manufacturer's instructions. Sequences of oligonucleotides and references are listed in **Table S4**. Twenty-four or 72 h after transfection, cells were harvested for western blot/RNA analysis or were detached and seeded in Agilent Seahorse XFe96 microplates for at least 4 h before Seahorse analyses.

PCSK9 overexpression experiments—H9c2 cardiomyocytes were transfected with PCSK9-GFP cDNA (1 μg) or empty control CT-GFP (Sinobiological) in Optimem Reduced Serum and Lipofectamine LTX and Plus Reagent (Invitrogen) according to the manufacturer's instructions. Twenty-four or 72 h after transfection, cells were harvested for western blot/RNA analysis or immunofluorescence staining.

Culture and transduction of human cardiomyocytes —Primary human cardiomyocytes isolated from normal human ventricle tissue of the adult heart were purchased from PromoCell® (C-12810, Sigma-Aldrich) and cultured according to the manufacturer's instructions. As a positive control for PCSK9 detection, primary human cardiomyocytes were transduced with an adeno-associated virus overexpressing a gain of function mutant (D374Y) of human PCSK9 (AAV9-HRC-hAAT-PCSK9DY, Vigene Biosciences) for 24 h.

PCSK9 analysis in plasma—Plasma PCSK9 levels were measured with a mouse Proprotein Convertase 9/PCSK9 Quantikine ELISA Kit (R&D Systems).

Lipidomics—Samples were homogenized in methanol with a Precellys 24 homogenizer (Bertin Technologies) and Mixer Mill equipment (Retsch). Lipids were extracted using the Folch procedure.[7] Internal standards containing heptadecanoyl (C17:0) were added during the extraction. The extracts were evaporated with nitrogen, reconstituted in chloroform:methanol [2:1], and stored at –20ºC. Phospholipids and sphingomyelin were quantified by direct infusion/mass spectrometry as described.[8] Ceramides, dihydroceramides, glucosylceramides, and lactosylceramides were quantified by ultra-performance liquid chromatography tandem mass-spectrometry.[9] The data were evaluated using the LipidView software (Sciex). Free and ester cholesterol from cells was quantified using straight phase HPLC coupled to ELS detection as described previously.[10]

Immunohistochemistry and immunofluorescence—Mouse hearts were embedded in OCT Cryomount (Histolab Products), frozen in liquid nitrogen, cooled in isopentane, and cut into 10-µm thick cross sections. For staining with wheat germ agglutinin (WGA, Alexa Fluor® 594 wheat germ agglutinin, Life Technology, W11262) and phalloidin (Alexa Fluor® 488 phalloidin, Life Technology, A12379), the sections were fixed in 10% formaldehyde for 5 min, rinsed in PBS, incubated for 10 min at 37°C with WGA conjugated with Alexa Fluor 594 (Invitrogen) (10 µg/ml), and rinsed three times in PBS. The sections were then stained with DAPI (4′,6-diamidino-2-phenylindole), washed in water, and mounted with Prolong Gold. Myocyte cross-sectional area was quantified with ImageJ.

For fibrosis assessment, heart sections were fixed in 10% formaldehyde without methanol for 5 min, rinsed in running tap water, stained in 0.1% Sirius Red solution for 1 h, and washed twice in acidified water. The sections were dehydrated in ethanol (70% and 95% for 30 s each, and 100% for 2 min), cleared in TissueClear solution, and mounted in a resinous medium (Eukitt, Sigma). Fibrosis was measured with ImageJ as the Sirius Red–stained area and expressed as percent of total area. High-resolution images covering the entire heart were obtained with a Mirax digital slide scanner (Carl Zeiss) for analysis.

Mouse RNA-seq— Total RNA in snap-frozen mouse tissue was extracted with the RNeasy Fibrous Tissue Kit or RNeasy Lipid Tissue Mini Kit (Qiagen). The purity and integrity of RNA samples were measured with an Agilent 2100 Bioanalyzer and the RNA 6000 Nano LabChip kit (Agilent Technologies). All samples had a 260/280 nm ratio ≥2.0, and RNA integrity numbers were ≥8. RNA-seq libraries were prepared with Illumina RNA-Seq with Poly-A selections and sequenced on NovaSeq6000 (NovaSeq Control Software 1.6.0/RNA v3.4.4) with a 2x51 setup using 'NovaSeqXp' workflow in 'S1' mode flow cell. The Bcl was converted to FastQ with bcl2fastq_v2.19.1.403 in the CASAVA software suite (Sanger/phred33/Illumina 1.8+ quality scale).

Analysis of RNA-seq data—Raw RNA-seq results were quantified with Kallisto [11]. A mapping file retrieved from Ensembl Biomart [12] was used to remove the non–protein coding genes and transcripts from the estimate count and transcripts per million reads files. The estimated count table was used as the input for DESeq2[13] package in R. A false-discovery rate (FDR) <0.05 was used to define differentially expressed genes. The fold change and p values from DESeq2 were

used for functional analysis with PIANO [14] with gene-set collections (Gene Ontology and KEGG Pathways) downloaded from Enrichr[15, 16] and Mouse Metabolic Reaction database.[17] An FDR <0.05 in distinct directionality columns in PIANO was used to define statistically significant processes or pathways. To visualize genes in specific functions or pathways, transcripts per million reads values were normalized with *zscore* in SciPy[18] and visualized with *clustermap* in the Seaborn package in Python 3.7.

Gene expression analysis—Total RNA in snap-frozen mouse tissue was extracted with the RNeasy Fibrous Tissue Kit or RNeasy Lipid Tissue Mini Kit (Qiagen). Total RNA from cultured isolated mouse cardiomyocytes, H9c2 cells and H9c2 cardiomyocytes was extracted with the RNeasy Mini Kit (Qiagen). RNA was quantified with a NanoDrop2000 spectrophotometer (Thermo Fisher Scientific). cDNA was synthesized with the high-capacity cDNA Reverse Transcription Kit (Applied Biosystems) and random primers. cDNA was amplified by quantitative real-time PCR with the SsoAdvanced Universal SYBR Green (Biorad) or TaqMan Fast Advanced Master Mix (Thermo Fisher Scientific). mRNA expression was normalized to HPRT mRNA expression with the ΔΔCT method and expressed as fold change versus controls. Sequences of primers and Taqman references for real-time PCR are listed in **Tables S5 and S6**.

Immunoblotting—Frozen heart tissue was homogenized, and the proteins were extracted with the Qprotome Mammalian Protein Prep kit (Qiagen). Cells were lysed with RIPA Cell Lysis Buffer (10X) (Cell Signaling) supplemented with phenylmethylsulfonyl fluoride. Once denatured, equal amounts of total protein lysate were loaded and separated on a NuPAGE 4–12 % Bis-Tris gel or a NuPAGE 4-20% Tris-Glycine gel (Invitrogen) and transferred to a PVDF membrane (Bio-Rad), previously activated in 100% methanol for 5 min. The membrane was then blocked in a solution of TBS, Tween 20, and bovine serum albumin (BSA, 3% minimum) for 1 h at room temperature (RT). Blots were probed with specific antibodies overnight at 4°C and then with the corresponding horseradish peroxidase–conjugated secondary antibody for 90 min at RT, washed with TBS-Tween, and visualized with Immobilon Western Chemiluminescent Horseradish Peroxidase Substrate (Millipore) and a ChemiDoc Touch Imaging System (BioRad). Bands were quantified with Image Lab Software (Bio-Rad) and normalized to vinculin, GAPDH or β-actin. All antibodies used for western blot are listed in **Table S7**.

Of note, we tested numerous commercially available antibodies generated against PCSK9 using tissue from wild-type (*Pcsk9*$^{+/+}$) and total *Pcsk9* knockout (*Pcsk9*$^{-/-}$) mice to test the specificity of the antibodies. Only one PCSK9 antibody (Abcam ab125251) was specific for mouse PCSK9 (**Figure 1F**). Thus, caution and appropriate controls are required in studies of PCSK9 expression in mice.

Mitochondria isolation—Hearts were excised, washed with ice-cold 0.9% NaCl, and crushed with a tissue grinder in mitochondrial isolation buffer (0.3 M sucrose, 5 mM TES, and 200 µM EGTA, pH 7.2) supplemented with proteinase bacterial (Sigma-Aldrich). Mitochondrial isolation buffer supplemented with BSA (1 mg/ml) was added, and the homogenates were centrifuged at 500*g* for 10 min at 4°C. The supernatant was collected and centrifuged at 3000*g* for 10 min at 4°C. The pellet containing the mitochondrial fraction was suspended in mitochondrial isolation buffer without BSA, and protein content was estimated with the BCA assay. Mitochondria were used immediately for Seahorse and membrane fluidity analysis or pelleted and kept at −80 °C for lipidomics, analysis of mitochondrial supercomplexes, and in-gel activity.

Cardiolipin analysis—Mitochondrial lipids were extracted using the Folch procedure and evaporated under a stream of nitrogen. The dry samples were reconstituted in heptane:isopropanol [9:1] and injected onto a 4.6 x 100 mm silica column (Spherisorb, Waters, Milford, MA). Separation was performed as described previously.[10] The cardiolipin fraction was

then evaporated together with an internal standard (Cardiolipin 14:1, Avanti Polar Lipids, Alabama, USA) and reconstituted in chloroform:methanol [1:2] with 5 mM ammonium acetate. A QTRAP 5500 mass spectrometer (Sciex, Concord, Canada) equipped with a robotic nanoflow ion source (TriVersa NanoMate, Advion BioSciences, Ithaca, NJ) was used for determination of cardiolipin species composition. Cardiolipin species (as [M-2H]2- ions) were detected by multiple precursor ion scanning as described previously.[19]

Acylcarnitine analysis—Mitochondrial acylcarnitines were extracted using a mixture of methanol and acetonitrile (1:3, v:v) containing eight isotopically labeled reference standards (NSK-B, Cambridge Isotopes Laboratories). After 10 minutes of vortex and 10 minutes of centrifugation at 4000*g*, the supernatant was diluted three times in methanol and acetonitrile (1:3, v:v) and then analysed using ultra-performance liquid chromatography-tandem mass spectrometry (UPLC-MS/MS). Briefly, the acylcarnitines were separated on a BEH Amide column (1.7 µm, 2.1 x 100 mm; Waters, USA) using acetonitrile and water with 10 mM ammonium acetate and 0.2% formic acid (95:5, v:v) and water with 10 mM ammonium acetate and 0.2% formic acid (v:v) as mobile phases. The acylcarnitines were detected using MRM in positive mode on a QTRAP 5500 mass spectrometer (Sciex, Concord, Canada). Quantification was made using the internal standards.

Transmission electron microscopy (TEM) of mouse hearts—Hearts were removed and fixed by vascular perfusion using a mixture of electron microscopy grade 2% paraformaldehyde, 2.5% glutaraldehyde, and 0.02% sodium azide in 0.05 M sodium cacodylate buffer, pH 7.2, for 5 min. Whole hearts were kept in the same fixative overnight. Tissue blocks (~1 mm^3) from the LV were collected, incubated overnight with 0.5 % uranyl acetate in water at 4°C, and postfixed for TEM (2 h in 1% osmium tetroxide (OsO_4)/1% potassium hexacyanoferrate in 0.1 M cacodylate, and 1 h in 1% tannic acid) or scanning electron microscopy (1 h in OsO_4, 20 min in thiocarbohydrazide, and 1 h in OsO_4). After routine dehydration and infiltration with Durcupan resin (Sigma) using an EM AMW microwave processor (Leica Microsystems), specimens were cured for 48 h at 60°C and cut with Leica UC6 ultramicrotome (Leica Microsystems, Vienna, Austria) fitted with diamond knives (Diatome). Ultrathin sections were collected on copper grids for imaging with a transmission electron microscope (FEI Talos120, Thermo Fisher) or on silica wafers for imaging with a scanning electron microscope (Gemini 450, Carl Zeiss NTS).

Mitochondria and cristae morphology analysis—TEM images were processed with RIJI/ImageJ to remove uneven illumination during tile scanning. Mitochondria in the TEM images were then segmented manually. Two-dimensional (2D) morphometric analysis was done by importing the segmented masks to MATLAB (2020a, MathWorks) for further shape analysis. Different descriptors of mitochondria morphology (area, perimeter, minimum/maximum Feret diameters, circularity, eccentricity, solidity and aspect ratio) were assessed. The 2D maximum and minimum Feret diameters were calculated along the region's convex hull, and are the longest and shortest distance respectively between two lines tangential to the mitochondrion measured. The aspect ratio is the value given by (maximum Feret)/(minimum Feret) of the mitochondrion; a higher aspect ratio implies a more elongated region. Circularity [4π·(surface area/perimeter2)] has values between 0 and 1, where 1 indicates a perfect circle. Eccentricity (0=circular, 1=line-segment) is a measure of the elongation of a 2D object compared to an ellipse that has the same second-moments as the object being analysed. Solidity has values between 0 and 1 (1=convex region such as a rectangle; <1=nonconvex region, such as an "L" shape) and is defined as the fraction of the pixels in the convex hull of a region that are also in the region. Cristae width was determined from ≥300 cristae; cristae density was defined as the total cristae length per mitochondrial area calculated from 30 mitochondria. FIJI software was used for both measurements.

Metabolic experiments on isolated primary cardiomyocytes—Metabolic profiling of primary cardiomyocytes was done with a Seahorse XF96 Extracellular Flux Analyzer (Seahorse Bioscience, Agilent). Cardiomyocytes were isolated from 28-week-old CM-*Pcsk9*$^{+/+}$ and CM-*Pcsk9*$^{-/-}$ mice, seeded at a density of 2000 cells/well on laminin-coated Seahorse XF-96 plates, and analysed as described below. OCR and ECAR values were normalized to total cellular protein content for each well (12 wells from 9 mice per group). Data were analysed with Seahorse Wave software.

Mitochondrial respiration (Mito Stress test)—After 2 h of cell attachment, the medium was replaced with XF basal medium supplemented with 10 mM glucose, 4 mM glutamine, and 1 mM sodium pyruvate (pH 7.4) and incubated for 1 h at 37°C in a CO_2-free incubator (for experiments with pyruvate/glucose as substrate) or with low-glucose (1 g/l) DMEM and 0.5 mM carnitine overnight (for experiments with palmitate as substrate) to induce glucose deprivation. The next day, the medium was replaced with XF basal medium supplemented with 2 mM glucose, 4 mM glutamine, 0.5 mM carnitine and incubated for 1 h at 37°C in a CO_2-free incubator; palmitate-BSA or BSA control (150 µM) was added to appropriate wells before running. The hydrated wells of the sensor cartridge were loaded with oligomycin (final concentration 1 µM, port A), carbonyl cyanide 4-(trifluoromethoxy) phenylhydrazone (FCCP) (1 µM, port B), and antimycin A + rotenone (2 µM port C). OCR was measured during sequential incubations to determine (1) basal respiration, (2) maximal respiration (FCCP), and (3) nonmitochondrial respiration (antimycin A/rotenone). Parameters were calculated following the instructions of Seahorse XF Cell Mito Stress Test Report Generator.

Fatty acid oxidation (FAO)—To assess FAO, endogenous substrates were depleted by replacing the culture medium with low-glucose (1 g/l) DMEM supplemented with 0.5 mM carnitine (for CPT-1 function) (pH 7.4) and incubating the cells overnight. One hour before the assay, the culture medium was replaced with XF basal medium supplemented with 2 mM glucose, 4 mM glutamine, and 0.5 mM carnitine (pH 7.4). The hydrated wells of the sensor cartridge were then loaded with oligomycin, FCCP, and antimycin A + rotenone as described above. Before measurements, cells were treated with 100 µM of etomoxir, a CPT-1 inhibitor for 15 min. At time 0, a saturating amount of palmitate-BSA (XF palmitate–BSA FAO substrate, Seahorse Bioscience, Agilent Technology) was added. This test reveals the portion of the OCR signal generated by endogenous and total FAO (from endogenous and exogenous FA). These parameters were calculated with normalised ECAR values using the equations given below:

- Basal respiration due to utilisation of exogenous FAs (Last OCR measurement before addition of oligomycin) = Palmitate/ Vehicle – Palmitate/Etomoxir
- Maximal respiration due to utilisation of exogenous FAs (Maximum OCR measurement after addition of FCCP) = Palmitate/ Vehicle – Palmitate/Etomoxir
- Basal respiration due to utilisation of endogenous FAs (Last OCR measurement before addition of oligomycin) = BSA/ Vehicle – BSA/Etomoxir
- Maximal respiration due to utilisation of endogenous FAs (Maximum OCR measurement after addition of FCCP) = BSA/Vehicle – BSA/Etomoxir

Glucose oxidation—Glucose catabolism was assessed by quantifying ECAR after first starving the cells of glucose and then adding saturating amounts of glucose to fuel both glycolysis and OXPHOS, followed by oligomycin (ATP synthesis inhibitor) and 2-deoxyglucose (glucose catabolism inhibitor). After cell adhesion, the medium was replaced with low-glucose (1 g/l) DMEM and plates were incubated overnight. The next day, the medium was replaced with XF basal medium supplemented with 2 mM glucose and 4 mM glutamine (pH 7.4), and the cells were incubated for 1 h at 37 °C in a CO_2-free incubator. The hydrated wells of the sensor cartridge were loaded with D-glucose (final concentration 10 mM, port A), oligomycin (2 µM , port B),

and 2-deoxyglucose (100 mM , port C). Parameters were calculated with normalized ECAR values using the equations given below:

- Basal acidification = Last ECAR measurement before addition of glucose – Minimum ECAR measurement after 2-DG treatment (meaning No glycolytic acidification parameter)
- Glucose oxidation = Maximum ECAR measurement after addition of glucose − Minimum ECAR measurement after 2-DG treatment (meaning No glycolytic acidification parameter)
- Glycolytic capacity = Maximum ECAR measurement after oligomycin treatment − Minimum ECAR measurement after 2-DG treatment (meaning No glycolytic acidification parameter)
- Glycolytic reserve = Glycolytic capacity parameter – Glucose oxidation parameter
- No glycolytic acidification = Minimum ECAR measurement after 2-DG treatment

Glycolytic rate—After 2 h of cell attachment, the medium was changed to Seahorse XF Base medium, supplemented with 10 mM glucose, 4 mM glutamine, and 1 mM sodium pyruvate, pH 7.4. ECAR and OCR were recorded with the Seahorse XF96e analyser at baseline, and after injection of rotenone/antimycin A (0.5 µM, port A) and then 2-deoxyglucose (100 mM, port B) as final concentrations. This test provides a precise measure of glycolytic rate by calculating and subtracting mitochondrial-produced acidification. Parameters were calculated following the instructions of Seahorse XF Glycolytic Rate Assay Report Generator.

Intracellular and extracellular lactate levels—Intracellular and extracellular lactate concentrations were measured in cardiomyocytes isolated from 28-week-old CM-*Pcsk9*$^{+/+}$ and CM-*Pcsk9*$^{-/-}$ mice after addition of mitochondrial inhibitors (2 µM rotenone + 2 µM antimycin; 15 min), glycolysis inhibitor (100 mM 2-deoxyglucose; 1 h) or no compound (1 h) with a bioluminescent-based assay according to the manufacturer's protocols (Lactate-Glo™, Promega). After treatment, cells were washed and then treated with an inactivation solution (0.6 M HCl) and neutralization solution (1 M Tris base) to inactivate the endogenous lactate dehydrogenase and to prevent NADH degradation. The culture media and cell lysates were then transferred to a 96-well plate and incubated with the assay reagent for 60 min. Luminescence signal was detected by a microplate reader. Lactate concentration was normalized to total protein content.

Blue native PAGE (BN-PAGE) analysis of mitochondrial supercomplexes and in-gel activity—BN-PAGE was done with the NativePAGE system (Invitrogen). Briefly, 100 µg of isolated cardiac mitochondria was solubilized with digitonin (8 g/g protein) for 20 min on ice and centrifuged at 20,000*g* for 10 min at 4°C. Coomassie blue G-250 (Invitrogen) was added to the supernatant to obtain a dye/detergent concentration ratio of ¼, and the protein was loaded into a 4–12% nondenaturing polyacrylamide gel (Invitrogen). After BN-PAGE, the activity of the complexes and supercomplexes was assessed in-gel and normalized to total mitochondria protein content determined by Coomassie staining.

For in-gel analysis of complex I activity, Tris-HCl (2 mM, pH 7.4), NADH (0.1 mg/ml), and nitro blue tetrazolium chloride (NTB, 2.5 mg/ml) in water were added to the gel after electrophoresis and incubated for 15–20 min at RT. The reaction was stopped with 10% acetic acid. Activity was determined by analysing the violet bands with Image J.

For in-gel analysis of complex II activity, 10 ml of substrate solution consisting of 1 M sodium succinate (1 M, 200 µl), NTB (25 mg), phenazine methosulphate (250 mM, 8 µl), and 5 mM Tris-HCl (5 mM) in water was added to the gel after electrophoresis and incubated for 40 min at RT. The reaction was stopped with 10% acetic acid. Activity was determined by analysing the violet bands with Image J.

For in-gel analysis of complex IV activity, 100 ml of substrate solution consisting of diaminobenzidine (50 mg), cytochrome C (100 mg), and phosphate buffer (50 mM, pH 7.4, 90 ml) in 10 ml of water was added to the gel after electrophoresis and incubated for 40 min at RT. The reaction was stopped with 10% acetic acid. Activity was determined by analysing the brown bands with Image J.

For in-gel analysis of complex V activity, Tris (35 mM), glycine (270 mM), $MgSO_4$ (14 mM), ATP (10 mM), and $Pb(NO_3)_2$ (0.2%) in water were added to the gel after electrophoresis and incubated overnight at RT. The reaction was stopped with 50% methanol. Silver bands indicate complex V activity. Since the bands are transparent, the gel was inverted (to black background) after scanning, and activity was determined with Image J.

Metabolic experiments on isolated mitochondria—After isolation, cardiac mitochondria (2 µg) were plated in each well of the Agilent Seahorse XFe96 microplate in 25 µl of ice-cold 1x mitochondrial assay (MAS) buffer consisting of 70 mM sucrose, 220 mM mannitol, 10 mM KH_2PO_4, 5 mM $MgCl_2$, 2 mM HEPES, 1 mM EGTA, and 0.2% fatty acid–free BSA, pH 7.2 at RT. The XF plate was centrifuged at 4°C for 20 min at 2000 rpm in a swinging bucket rotor and experiments were performed as described below. OCR values were normalized to total mitochondrial protein content for each well (8–12 wells from 7–9 mice per group).

Electron flow assay—The mitochondrial respiratory complex activity was assessed by measuring uncoupler-stimulated respiration in the presence of various substrates. The assay measures each complex of the ETC sequentially: (1) respiration driven by complex I in an uncoupled state (pyruvate + malate + FCCP), (2) inhibition of complex I (rotenone), (3) restart respiration by complex II (succinate), (4) inhibition of complex III (antimycin A), and (5) restart respiration by complex IV (ascorbate and TMPD, which act as electron donors to cytochrome C/complex IV). While the plate was centrifuged, the XF cartridge was prepared by injecting 20 µM rotenone (20 µl, Port A), 50 mM succinate (22 µl, Port B), 40 µM antimycin A (25 µl. Port C), and 100 mM ascorbate + 1 mM TMPD (27 µl, port D). The final concentrations were 2 µM rotenone, 5 mM succinate, 4 µM antimycin A, and 10 mM ascorbate + 0.1 mM TMPD. After centrifugation, 155 µl of 1x MAS supplemented with 10 mM pyruvate, 5 mM malate, and 4 µM FCCP was added, and the plate was introduced into the machine to monitor mitochondrial OCR after the sequential addition of drugs.

Coupling assay—This assay measures coupling of the ETC and OXPHOS machinery. Respiration was initiated by adding ADP (5 mM, State III) and stopped by adding oligomycin (2.5 µM, State IV). FCCP dissipated mitochondrial membrane potential and increased respiratory rates (4 µM, State IIIu). Finally, mitochondrial respiration was definitively stopped by adding antimycin A and rotenone (4 µM). The respiratory control ratio (State III/State IV) served as an indicator of mitochondrial coupling. While the plate was centrifuged, the XF cartridge was prepared by injecting 50 mM ADP (20 µl, Port A), 25 µM oligomycin (22 µl, Port B), 40 µM FCCP (25 µl, Port C), and 40 µM rotenone and 40 µM antimycin A (27 µl, port D). After centrifugation, 155 µl of 1x MAS supplemented with 10 mM pyruvate and 5 mM malate for Cx I-driven respiration or with 5 mM succinate and 2 µM rotenone or 40 µM palmitoylcarnitine/1 mM malate for Cx II-driven respiration; was added, and the plate was introduced into the machine to monitor mitochondrial OCR after the sequential addition of drugs.

Mitochondrial ROS measurement—Cells were incubated with 5 µM MitoSOX Red probe (Thermo Scientific) in the dark at 37°C for 10 min and washed with PBS. This probe penetrates living cells and selectively targets mitochondria to measure mitochondrial ROS production. The probe is oxidized by superoxide ions, resulting in red fluorescence, which was measured using a fluorimeter at 580 nm after excitation at 510 nm.

Mitochondrial H_2O_2 production measurement—H_2O_2 production in isolated mitochondria was measured using the Amplex® Red hydrogen peroxide kit (Molecular Probes). Briefly, isolated mitochondria were incubated in MAS buffer alone (basal conditions) or supplemented with 4 µM FCCP (to increase energy demand) or 4 µM antimycin (a positive control of massive ROS production) for 15 min. The isolated mitochondria were then incubated with 50 µM Amplex® Red reagent and 0.1 U/mL horseradish peroxidase in each well of a 96-well plate. The increase in Amplex red fluorescence was followed for 45 min at room temperature with excitation at 530 nm and emission at >590 nm in a 96-well microplate reader. H_2O_2 production was indexed by the increase of Amplex red fluorescence per min, per mg of mitochondrial protein.

Assessment of mitochondrial membrane potential—To monitor myocardial membrane potential, cells were incubated with 5 µM 5′,6,6′-tetrachloro-1,1′,3,3′-tetraethyl-benzamidazolocarbocyanin iodide (JC-1, Enzo Life Sciences) at 37°C for 15 min and washed with PBS. The dye was excited at 496 nm and the emission signal ratio (590/530 nm) was normalized to that measured under basal conditions. As a positive control, cells were treated with FCCP (10 min; 1 µM) to dissipate the mitochondrial membrane potential.

Metabolic experiments on mature H9c2 cardiomyocytes —After 5 days of differentiation in retinoic acid followed by transfection with siRNA or plasmid for 24 h, H9c2 cardiomyocytes were detached, seeded on Agilent Seahorse XFe96 microplates at a density of 25,000 cells/well, and incubated with high- or low-glucose DMEM supplemented with 10% fetal bovine serum, 100 U/ml penicillin. and 100 µg/ml streptomycin for at least 4 h before the Seahorse experiments described below. For experiments studying the effect of secreted PCSK9, *Pcsk9*-deficient H9c2 cardiomyocytes were treated with conditioned media from H9c2 cardiomyocytes overexpressing PCSK9-GFP cDNA (containing a high concentration of secreted PCSK9) or empty control CT-GFP (containing a low concentration of secreted PCSK9) for an additional 24 h before seeding on Agilent Seahorse XFe96 microplates.

OCR and ECAR values were normalized to total cellular protein content for each well (12 wells from 4–6 independent experiments). Data were analysed with Seahorse Wave software.

Mitochondrial respiration (Mito Stress test)—One hour before the assay, cells were incubated with XF basal medium supplemented with 10 mM glucose and 4 mM glutamine (pH 7.4) and incubated at 37°C in a non-CO_2 incubator. OCR was measured during sequential incubations to determine (1) basal respiration, (2) ATP production coupled to respiration (1 µM oligomycin), (3) maximal respiration (5 µM FCCP), and (4) nonmitochondrial respiration (5 µM antimycin A + 5 µM rotenone). Parameters were calculated following the instructions of Seahorse XF Cell Mito Stress Test Report Generator.

Fatty acid oxidation (FAO)—One hour before the assay, cells were incubated with XF basal medium supplemented with 1 mM glucose and 0.5 mM carnitine (pH 7.4). OCRs were measured after sequential addition of oligomycin, FCCP, antimycin A + rotenone as for mitochondrial respiration. Fifteen minutes before measurements, cells were treated with 40 µM etomoxir, a CPT-1 inhibitor. At time 0, 150 µM palmitate was added. Parameters were calculated using equations as described above for primary cardiomyocytes.

Glucose oxidation—One hour before the assay, cells were incubated with XF basal medium supplemented with 4 mM glutamine (pH 7.4). Extracellular acidification rate (ECAR) was measured at baseline and after the injection of glucose (10 mM), oligomycin (2 µM), and 2-deoxyglucose (2-DG, 50 mM). Parameters were calculated using equations as described above for primary cardiomyocytes.

ATP rate assay—This assay simultaneously measures the rates of ATP production from glycolysis (GlycoATP) and mitochondrial respiration (MitoATP) in live cells. One hour before the assay, cells were incubated with XF basal medium supplemented with 10 mM glucose and 4 mM glutamine (pH 7.4) to measure ATP produced from carbohydrates and with 1 mM glucose/0.5 mM carnitine (pH 7.4) to measure ATP produced from fatty acids (150 µM palmitate added just before running plate). OCRs and ECARs were measured after sequential addition of oligomycin and antimycin A with rotenone (all at 1 µM). Parameters were calculated following the instructions of Seahorse XF ATP Rate Assay Report Generator.

PCSK9 subcellular localisation—H9c2 cardiomyocytes were plated on glass coverslips. After 5 days of differentiation in retinoic acid followed by transfection with plasmid for 24 h, cells were fixed in 4% paraformaldehyde for 15 min. After permeabilisation in 0.1% Triton X-100 in PBS for 5 min and blocking, the cells were probed with chicken anti-GFP antibody (ab13970 Abcam) and mouse anti-KDEL (ER marker) antibody (ab1223 Abcam) or rabbit anti-GM130 (Cis-Golgi marker) antibody (ab52649 Abcam) or mouse anti-ATP5A (mitochondrial marker) (ab14748 Abcam) for 16 h at 4°C. Cells were then probed with anti-mouse or rabbit Alexa-Fluor 647–conjugated and anti-chicken Alexa-Fluor 488-conjugated secondary antibodies and mounted in ProLongTM Glass Antifade Mountant (P36984-Invitrogen). Confocal images of H9c2 cardiomyocytes expressing GFP-tagged PCSK9 were acquired with 63.0x objective lens with oil immersion using Leica TCS-SP5 with Leica LAS AF software (Leica Microsystems) using a pinhole of 1 Airy unit. Images were sampled according to Nyquist criteria and deconvoluted by using Huygens essential software; thereby optimizing images for accurate colocalization of fluorescent signals. For colocalisation analysis, the threshold of each channel used to quantify colocalisation was automatically determined using the method of Costes. Pearson's coefficient, calculated with Huygens essential software, was used to analyse colocalisation between GFP-PCSK9 and KDEL or GM130 or ATP5A. Analysis included combined measurement of z series stacks acquired at ~0.125 µm intervals through the total thickness of the cells.

Quantification and statistical analysis—Values are reported as mean ± SEM unless otherwise indicated. Details of the statistical analysis, including numbers of mice, are indicated in the figure legends. Unpaired two-tailed *t*-tests were used to compare two groups; two-way ANOVA followed by Sidak's multiple comparisons test was used to compare more than two groups. Survival was assessed with the log-rank test. $P < 0.05$ was considered statistically significant. GraphPad Prism was used for all statistical analyses and graphics.

REFERENCES

1. Haas ME, Levenson AE, Sun X, Liao WH, Rutkowski JM, de Ferranti SD, Schumacher VA, Scherer PE, Salant DJ, Biddinger SB. The role of proprotein convertase subtilisin/kexin type 9 in nephrotic syndrome-associated hypercholesterolemia. *Circulation* 2016;**134**:61-72.
2. O'Connell TD, Rodrigo MC, Simpson PC. Isolation and culture of adult mouse cardiac myocytes. *Methods Mol Biol* 2007;**357**:271-296.
3. Andersson L, Cinato M, Mardani I, Miljanovic A, Arif M, Koh A, Lindbom M, Laudette M, Bollano E, Omerovic E, Klevstig M, Henricsson M, Fogelstrand P, Sward K, Ekstrand M, Levin M, Wikstrom J, Doran S, Hyotylainen T, Sinisalu L, Oresic M, Tivesten A, Adiels M, Bergo MO, Proia R, Mardinoglu A, Jeppsson A, Boren J, Levin MC. Glucosylceramide synthase deficiency in the heart compromises beta1-adrenergic receptor trafficking. *Eur Heart J* 2021;**42**:4481-4492.
4. Ruiz M, Stahlman M, Boren J, Pilon M. AdipoR1 and AdipoR2 maintain membrane fluidity in most human cell types and independently of adiponectin. *J Lipid Res* 2019;**60**:995-1004.
5. Owen DM, Rentero C, Magenau A, Abu-Siniyeh A, Gaus K. Quantitative imaging of membrane lipid order in cells and organisms. *Nat Protoc* 2011;**7**:24-35.
6. Kankeu C, Clarke K, Van Haver D, Gevaert K, Impens F, Dittrich A, Roderick HL, Passante E, Huber HJ. Quantitative proteomics and systems analysis of cultured H9C2 cardiomyoblasts during differentiation over time supports a 'function follows form' model of differentiation. *Mol Omics* 2018;**14**:181-196.
7. Folch J, Lees M, Sloane Stanley GH. A simple method for the isolation and purification of total lipides from animal tissues. *J Biol Chem* 1957;**226**:497-509.
8. Stahlman M, Fagerberg B, Adiels M, Ekroos K, Chapman JM, Kontush A, Boren J. Dyslipidemia, but not hyperglycemia and insulin resistance, is associated with marked alterations in the HDL lipidome in type 2 diabetic subjects in the DIWA cohort: impact on small HDL particles. *Biochim Biophys Acta* 2013;**1831**:1609-1617.
9. Amrutkar M, Cansby E, Nunez-Duran E, Pirazzi C, Stahlman M, Stenfeldt E, Smith U, Boren J, Mahlapuu M. Protein kinase STK25 regulates hepatic lipid partitioning and progression of liver steatosis and NASH. *FASEB J* 2015.
10. Homan R, Anderson MK. Rapid separation and quantitation of combined neutral and polar lipid classes by high-performance liquid chromatography and evaporative light-scattering mass detection. *J Chromatogr B Biomed Sci Appl* 1998;**708**:21-26.
11. Bray NL, Pimentel H, Melsted P, Pachter L. Near-optimal probabilistic RNA-seq quantification. *Nat Biotechnol* 2016;**34**:525-527.
12. Zerbino DR, Achuthan P, Akanni W, Amode MR, Barrell D, Bhai J, Billis K, Cummins C, Gall A, Giron CG, Gil L, Gordon L, Haggerty L, Haskell E, Hourlier T, Izuogu OG, Janacek SH, Juettemann T, To JK, Laird MR, Lavidas I, Liu Z, Loveland JE, Maurel T, McLaren W, Moore B, Mudge J, Murphy DN, Newman V, Nuhn M, Ogeh D, Ong CK, Parker A, Patricio M, Riat HS, Schuilenburg H, Sheppard D, Sparrow H, Taylor K, Thormann A, Vullo A, Walts B, Zadissa A, Frankish A, Hunt SE, Kostadima M, Langridge N, Martin FJ, Muffato M, Perry E, Ruffier M, Staines DM, Trevanion SJ, Aken BL, Cunningham F, Yates A, Flicek P. Ensembl 2018. *Nucleic Acids Res* 2018;**46**:D754-D761.
13. Love MI, Huber W, Anders S. Moderated estimation of fold change and dispersion for RNA-seq data with DESeq2. *Genome Biol* 2014;**15**:550.
14. Varemo L, Nielsen J, Nookaew I. Enriching the gene set analysis of genome-wide data by incorporating directionality of gene expression and combining statistical hypotheses and methods. *Nucleic Acids Res* 2013;**41**:4378-4391.

15. Chen EY, Tan CM, Kou Y, Duan Q, Wang Z, Meirelles GV, Clark NR, Ma'ayan A. Enrichr: interactive and collaborative HTML5 gene list enrichment analysis tool. *BMC Bioinformatics* 2013;**14**:128.
16. Kuleshov MV, Jones MR, Rouillard AD, Fernandez NF, Duan Q, Wang Z, Koplev S, Jenkins SL, Jagodnik KM, Lachmann A, McDermott MG, Monteiro CD, Gundersen GW, Ma'ayan A. Enrichr: a comprehensive gene set enrichment analysis web server 2016 update. *Nucleic Acids Res* 2016;**44**:W90-97.
17. Mardinoglu A, Shoaie S, Bergentall M, Ghaffari P, Zhang C, Larsson E, Backhed F, Nielsen J. The gut microbiota modulates host amino acid and glutathione metabolism in mice. *Mol Syst Biol* 2015;**11**:834.
18. Jones E, Oliphant T, Peterson P. SciPy: open source scientific tools for python. 2001.
19. Ejsing CS, Duchoslav E, Sampaio J, Simons K, Bonner R, Thiele C, Ekroos K, Shevchenko A. Automated identification and quantification of glycerophospholipid molecular species by multiple precursor ion scanning. *Anal Chem* 2006;**78**:6202-6214.

Unedited gel for each representative cropped gel within the manuscript:

Figure 1		Figure 1B	Figure 1C	Figure 1F
Figure 2	no gels			
Figure 3		Figure 3C		
Figure 4	no gels			
Figure 5		Figure 5A	Figure 5B	Figure 5C
Figure 6	no gels			
Figure 7		Figure 7A		
Figure 8		Figure 8B		
Supplementary Figure 1	no gels			
Supplementary Figure 2	no gels			
Supplementary Figure 3	no gels			
Supplementary Figure 4	no gels			
Supplementary Figure 5		Figure S5C		
Supplementary Figure 6		Figure S6B	Figure S6E	Figure S6F
Supplementary Figure 7	no gels			
Supplementary Figure 8		Figure S8A		

Figure 1B

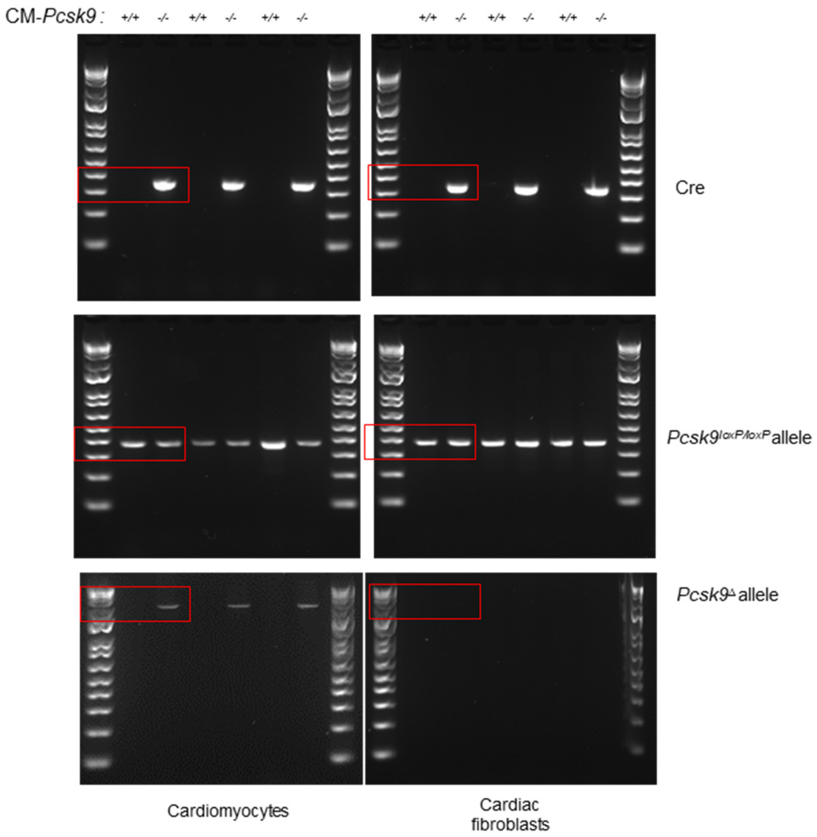

Figure 1C

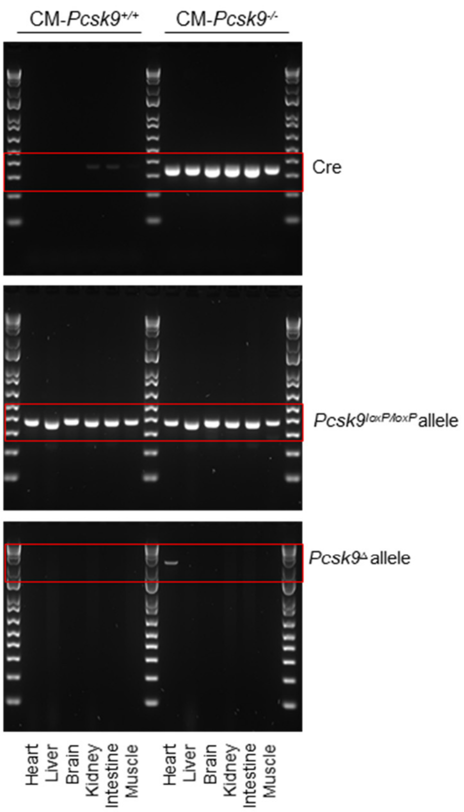

Figure 1F

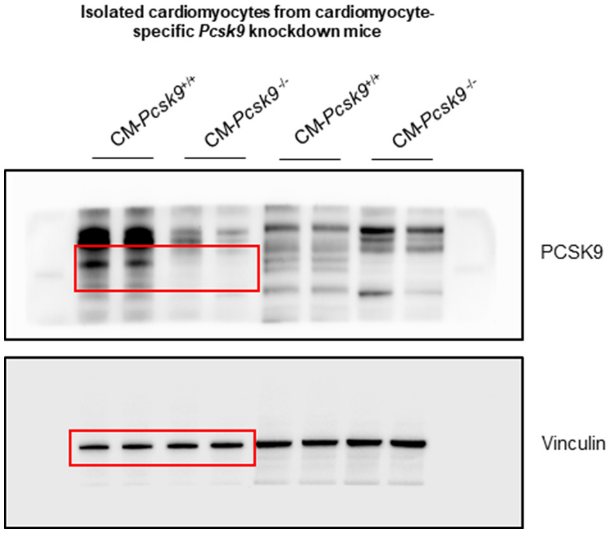

Isolated cardiomyocytes from cardiomyocyte-specific *Pcsk9* knockdown mice
CM-*Pcsk9*+/+
CM-*Pcsk9*-/-
CM-*Pcsk9*+/+
CM-*Pcsk9*-/-
PCSK9
Vinculin

Figure 3C

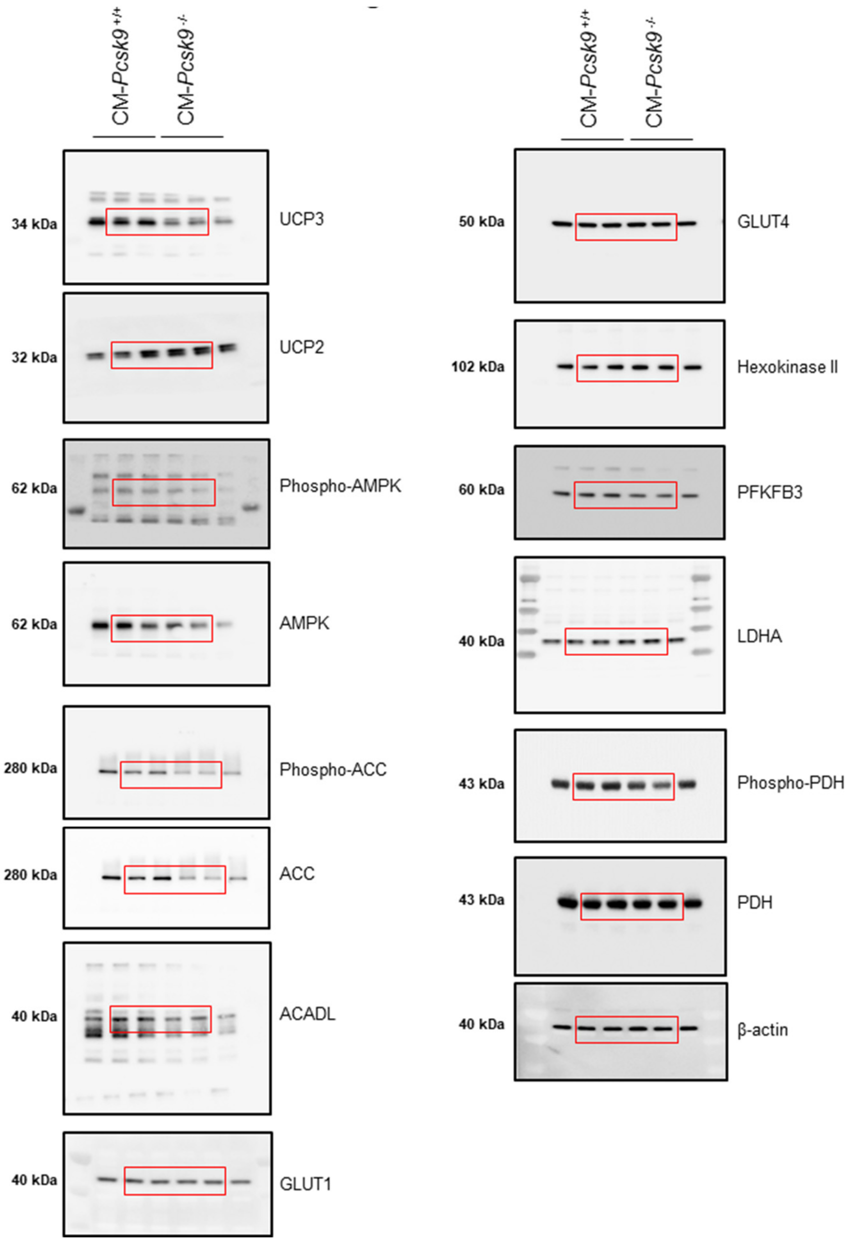
CM-Pcsk9 +/+
CM-Pcsk9 -/-
34 kDa
UCP3
32 kDa
UCP2
62 kDa
Phospho-AMPK
62 kDa
AMPK
280 kDa
Phospho-ACC
280 kDa
ACC
40 kDa
ACADL
40 kDa
GLUT1
50 kDa
GLUT4
102 kDa
Hexokinase II
60 kDa
PFKFB3
40 kDa
LDHA
43 kDa
Phospho-PDH
43 kDa
PDH
40 kDa
β-actin

Figure 5A

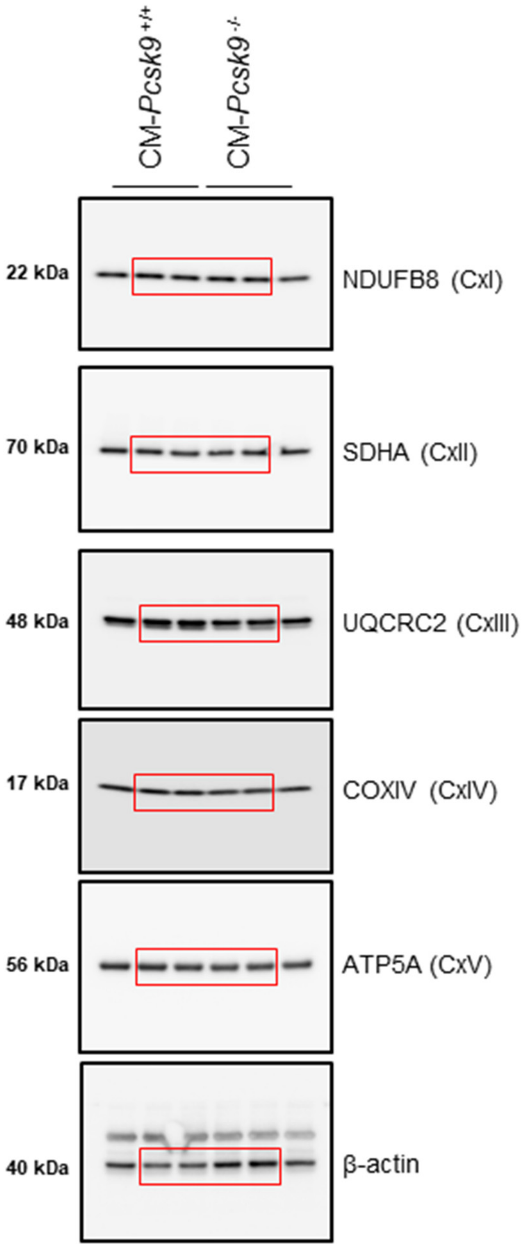

CM-Pcsk9 +/+
CM-Pcsk9 -/-
22 kDa
NDUFB8 (CxI)
70 kDa
SDHA (CxII)
48 kDa
UQCRC2 (CxIII)
17 kDa
COXIV (CxIV)
56 kDa
ATP5A (CxV)
40 kDa
β-actin

Figure 5B

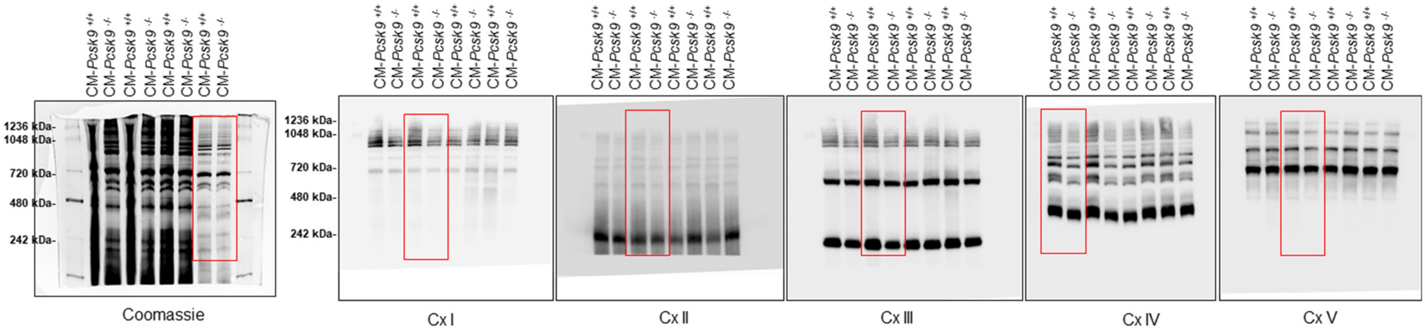

Figure 5C

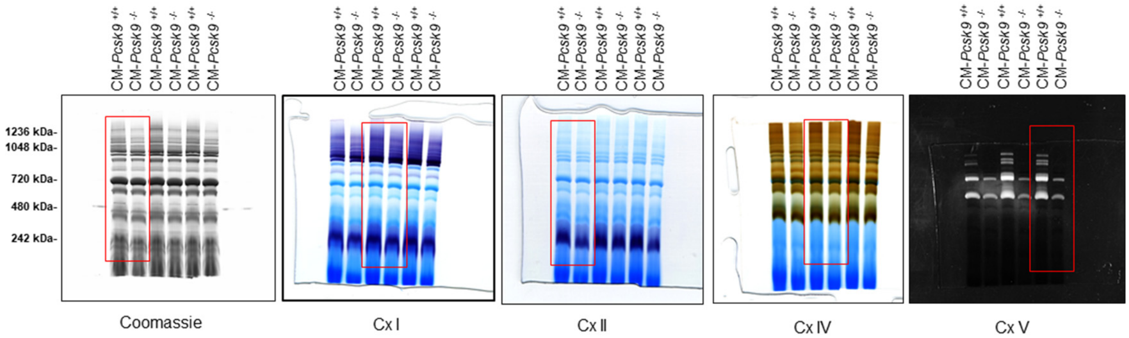

Figure 7A

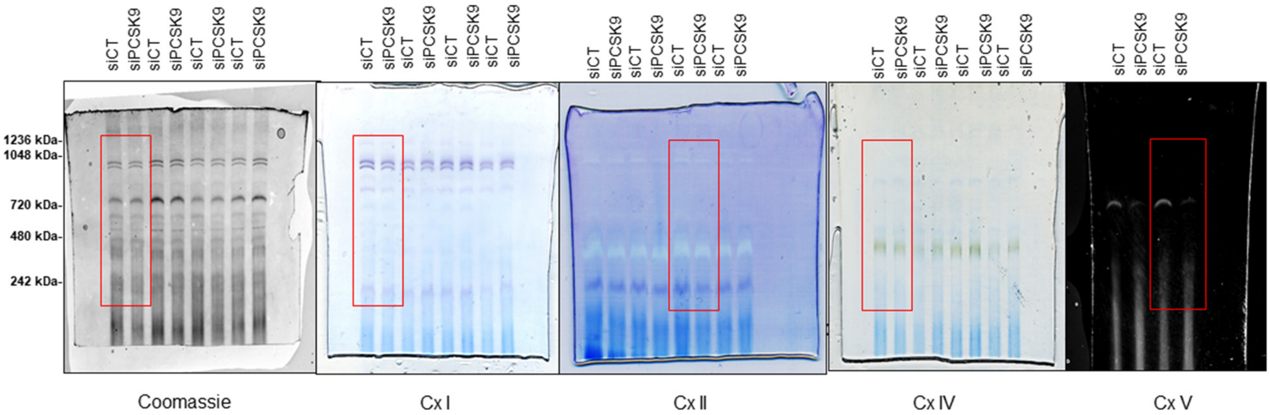

Figure 8B

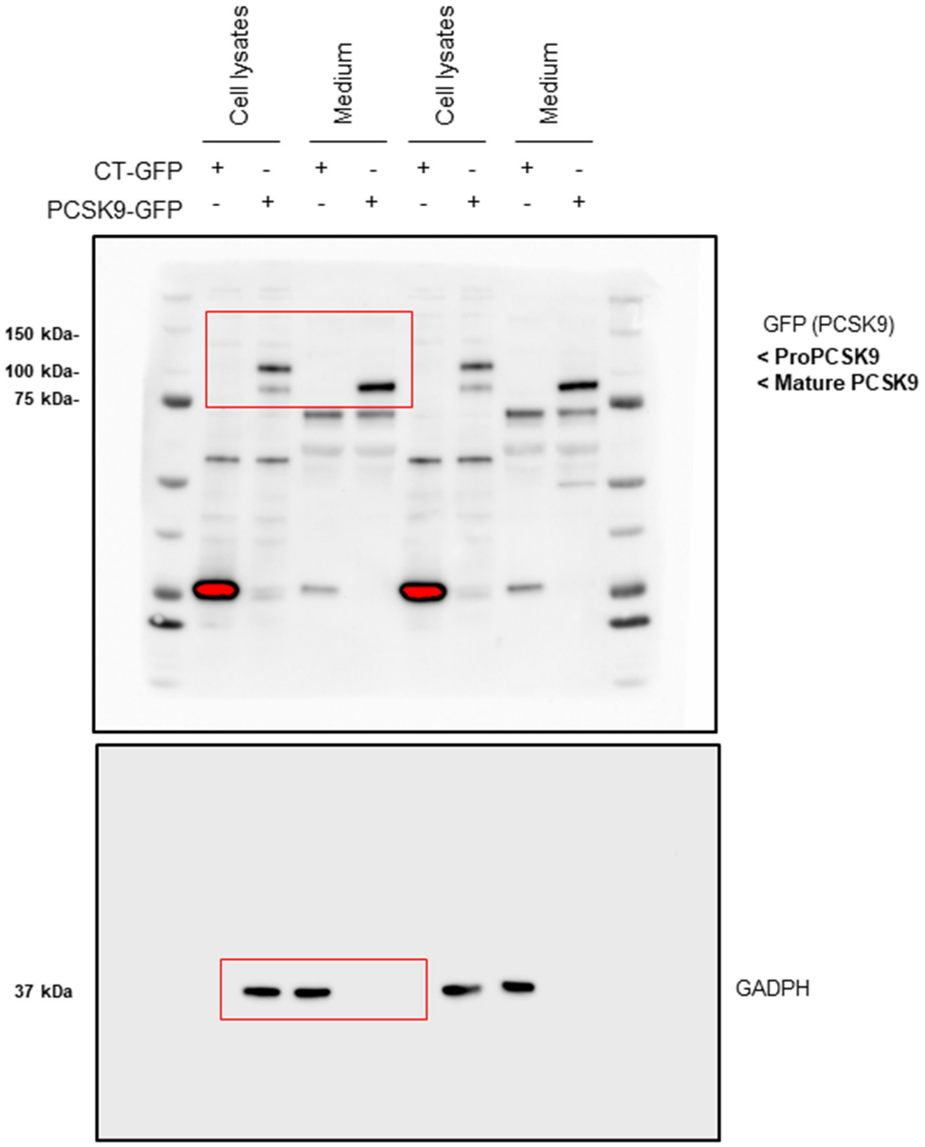
Cell lysates
Medium
Cell lysates
Medium
CT-GFP
PCSK9-GFP
150 kDa-
100 kDa-
75 kDa-
GFP (PCSK9)
< ProPCSK9
< Mature PCSK9
37 kDa
GADPH

Suppl Figure 5C

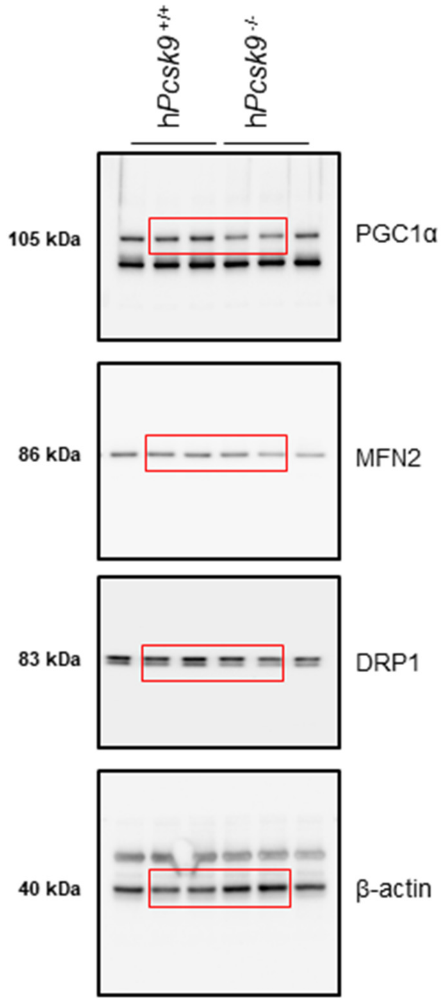

Suppl Figure 6B

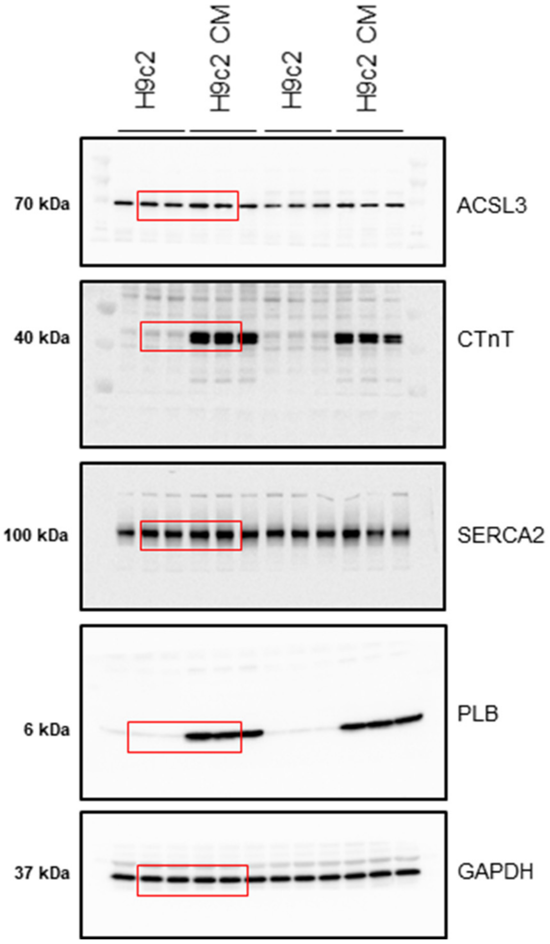

Suppl Figure 6E

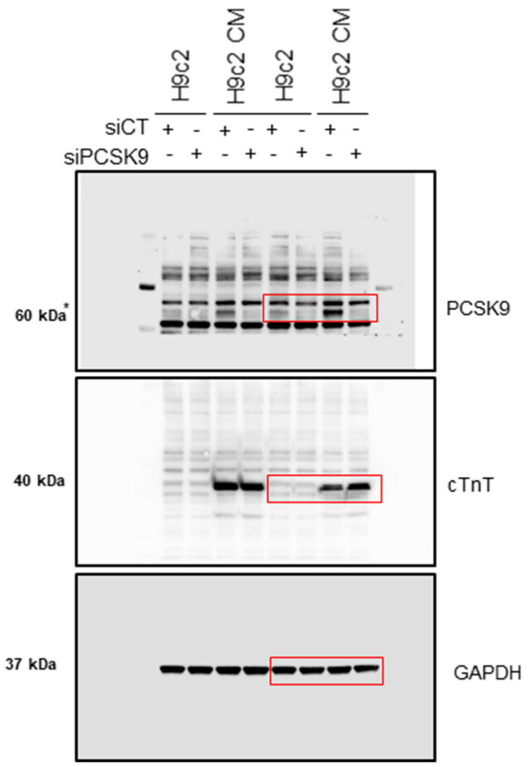

Suppl Figure 6F

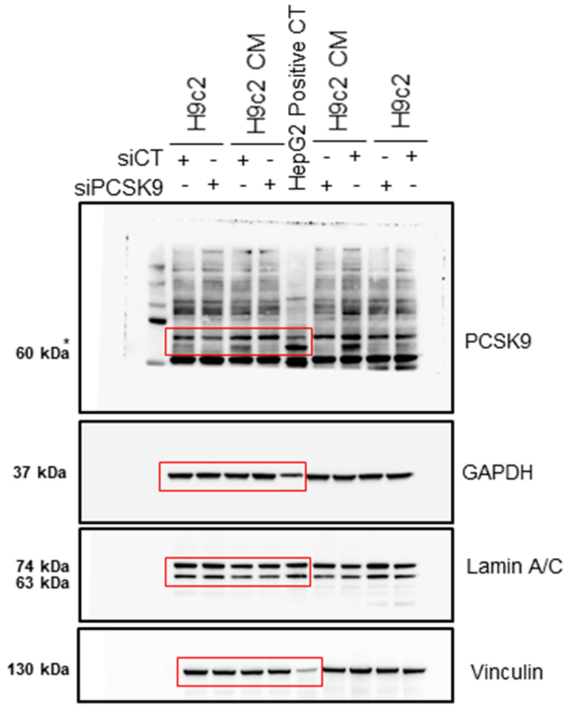

Suppl Figure 8A

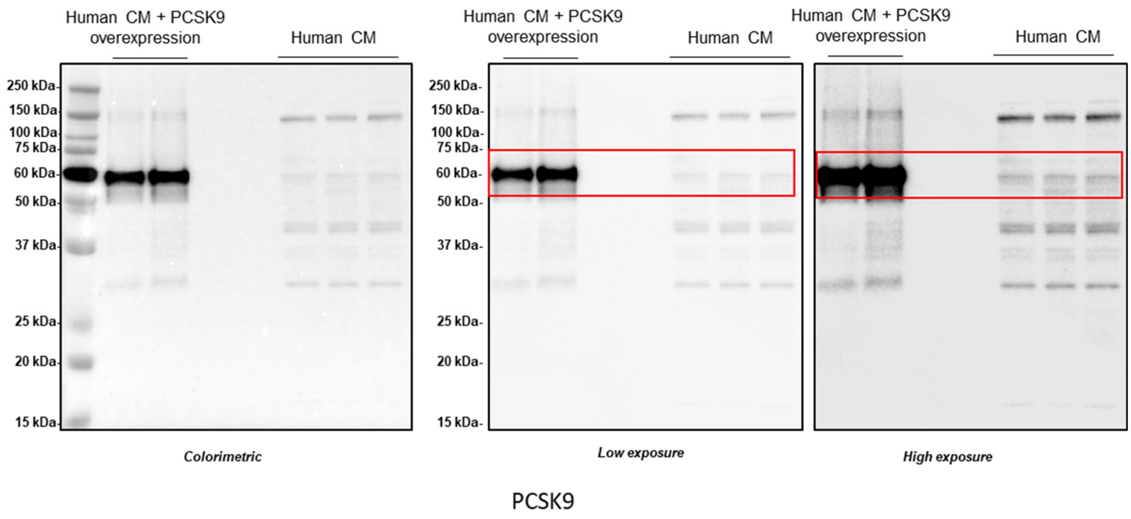

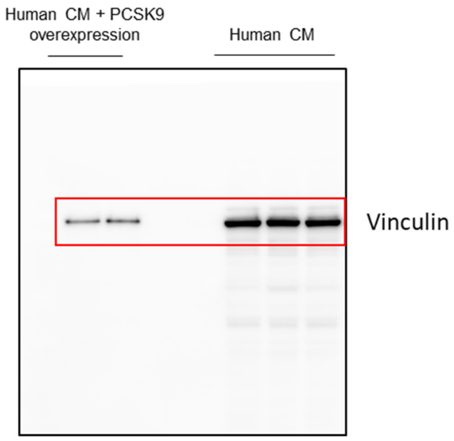

Supplement: cvad041_Supplementary_Data [file cvad041_supplementary_data.zip › Supplemental PCSK9 CLEAN R4 FINAL.pdf]
